# New Mammalian Target of Rapamycin (mTOR) Modulators Derived from Natural Product Databases and Marine Extracts by Using Molecular Docking Techniques

**DOI:** 10.3390/md16100385

**Published:** 2018-10-15

**Authors:** Verónica Ruiz-Torres, Maria Losada-Echeberría, Maria Herranz-López, Enrique Barrajón-Catalán, Vicente Galiano, Vicente Micol, José Antonio Encinar

**Affiliations:** 1 Institute of Research, Development and Innovation in Biotechnology of Elche (IDiBE) and Molecular and Cell Biology Institute (IBMC), Miguel Hernández University (UMH), Elche, 03202 Alicante, Spain; vruiz@umh.es (V.R.-T.); mlosada@umh.es (M.L.-E.); mherranz@umh.es (M.H.-L.); e.barrajon@umh.es (E.B.-C.); vmicol@umh.es (V.M.); 2 Department of Physics and Computer Architecture, Miguel Hernández University (UMH), Elche, 03202 Alicante, Spain; vgaliano@umh.es; 3 Centro de Investigación Biomédica en Red (CIBER) (CB12/03/30038), Fisiopatología de la Obesidad y la Nutrición, CIBERobn, Instituto de Salud Carlos III., 07122 Palma de Mallorca, Spain

**Keywords:** mTOR kinase, marine natural products, natural products, inhibitors, aging, obesity, cancer, virtual screening, molecular docking, calculated ADMET

## Abstract

Mammalian target of rapamycin (mTOR) is a PI3K-related serine/threonine protein kinase that functions as a master regulator of cellular growth and metabolism, in response to nutrient and hormonal stimuli. mTOR functions in two distinct complexes—mTORC1 is sensitive to rapamycin, while, mTORC2 is insensitive to this drug. Deregulation of mTOR’s enzymatic activity has roles in cancer, obesity, and aging. Rapamycin and its chemical derivatives are the only drugs that inhibit the hyperactivity of mTOR, but numerous side effects have been described due to its therapeutic use. The purpose of this study was to identify new compounds of natural origin that can lead to drugs with fewer side effects. We have used computational techniques (molecular docking and calculated ADMET (Absorption, Distribution, Metabolism, Excretion, and Toxicity) parameters) that have enabled the selection of candidate compounds, derived from marine natural products, SuperNatural II, and ZINC natural products, for inhibitors targeting, both, the ATP and the rapamycin binding sites of mTOR. We have shown experimental evidence of the inhibitory activity of eleven selected compounds against mTOR. We have also discovered the inhibitory activity of a new marine extract against this enzyme. The results have been discussed concerning the necessity to identify new molecules for therapeutic use, especially against aging, and with fewer side effects.

## 1. Introduction

**Figure 1:**
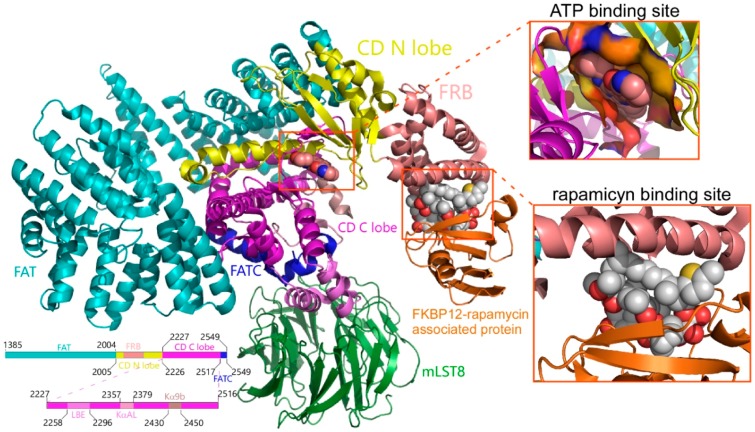
Secondary structure of the mTOR (residues 1376–2549)-SEC13 protein 8 (or mLST8)-FK506-binding protein 12 (or FKBP12)-ATP-rapamycin complex. mTOR presents different colors, indicating its structural domains [2]. mLST8 is colored green, KBP12 is colored orange, and ATP and rapamycin are shown as spheres. CD is the catalytic domain. The ATP and rapamycin binding sites were expanded in the boxes to the right. The figure was constructed using the structural information of the PDBs numbered as 4JSN, 4JSP, 4JSV, 4JSX, 4JT5, 4JT6, 5WBU, 5WBY, 1FAP, 1NSG, 2FAP, 3FAP, 4FAP, 5GPG, 4DRH, 4DRI, and 4DRJ, and PyMol 2.0 software (Schrödinger, Inc., New York, NY, USA) was used.

The mammalian target of rapamycin (mTOR) is a highly conserved phosphoinositide 3-kinase (PI3K-like) Ser/Thr protein kinase (UniProt P42345), which plays an important role in the center of numerous cellular signaling pathways that control the organization of the cell’s cytoskeleton, autophagy, metabolism, survival and proliferation, and integrates the growth factor-activated and nutrient-sensing pathways [1]. It is known that mTOR binds to different regulatory subunits and forms two types of protein complexes. First, mTORC1, which is sensitive to rapamycin and is a macrolide antibiotic produced by *Streptomyces hygroscopicus* that forms a complex with the immunophilin FK506 binding protein-12 (FKBP12) (Figure 1), which binds to the FKBP12-rapamycin binding (FRB) domain of mTOR [2] and inhibits its kinase activity [3]. Second, mTORC2, in which mTOR forms a protein complex insensitive to rapamycin [4]. In addition to mTOR, the mTORC1 complex contains the following proteins—regulatory-associated protein of mTOR (RAPTOR), proline-rich Akt substrate 40 kDa (PRAS40), mammalian lethal with Sec13 protein 8 (mLST8), and DEP domain containing mTOR-interacting protein (DEPTOR) [3].

In the plasma membrane, several receptors (GPCR, G protein-coupled receptor; IGF-R, insulin-like growth factor receptor; IR, insulin receptor) capture the signal transmitted by growth factors and chemokines, which act as positive inputs of the mTORC1 complex, directed mainly through two signaling pathways—PI3K/Akt [5] and Ras/Raf/MEK/ERK [6]. Additionally, nutrients such as glucose or amino acids and the cellular energy status (high ATP:AMP ratio) are inputs positive to the mTORC1 complex [7]. Low cellular energy levels are sensed by AMPK, which sequentially phosphorylates the tuberous sclerosis complex 2 (TSC2) and activates it, leading to the inhibition of the mTORC1 activity [8]. In poorly vascularized tumors, hypoxia conditions are predominant. Therefore, the complete oxidation of glucose to CO_2_, to achieve ATP and reduce power (NADH and FADH_2_), is impossible. Under these conditions of the lack of O_2_, glucose undergoes partial oxidation until pyruvate (glycolysis) in the cellular cytoplasm and the NADH generated is re-oxidized, giving its electrons to pyruvate that becomes lactate (Warburg effect) [9]. These conditions of acidity [10] and hypoxia [11] impede the activity of mTORC1. On the other hand, several extracellular signals [insulin, epidermal growth factor (EGF), vascular endothelial growth factor (VEGF), sphingosine 1-phosphate (S1P), and lysophosphatidic acid] stimulate phospholipase D, which converts phosphatidyl choline (PC) into phosphatidic acid (PA) [12]. PA species with unsaturated fatty acid chains can dissociate DEPTOR from mTORC1 and, thus, increase its activity [13].

An active mTORC1 complex controls protein biosynthesis because it directly phosphorylates two components of the biosynthetic machinery—p70 ribosomal S6 kinase 1 (S6K1-Thr^389^) and the translation inhibitor eukaryotic translation initiation factor 4E-binding protein 1 (4E-BP1) [14,15]. Only when E4-BP1 has been phosphorylated, can it be bound to eIF4E. As a result, this protein can be part of the eIF4F complex, which is required for the initiation of a cap-dependent mRNA translation [14]. mTORC1 also controls both membrane lipid biosynthesis, especially through two transcription factors of lipogenic genes, namely, SREBP 1/2 and PPAR-γ, and the genes of the glycolytic pathway [7]. By contrast, mTORC1 is a negative regulator of autophagy because it directly phosphorylates and suppresses some components of the ULK1/Atg13/FIP200 complex, which must remain active to initiate the process of autophagy [16]. In vascularized tumors, mTORC1 plays an important role as a central mediator of signal transducer and activation of transcription 3 (STAT3), hypoxia-inducible factor 1α (HIF-1α), vascular endothelial growth factor A (VEGF-A), and angiogenesis, under hypoxia, through several signaling mechanisms [17].

The biology of the mTORC2 complex (DEPTOR, SIN1, RICTOR, mLST8, and PROTOR) is less known but its activity is controlled by the PI3K/Akt and WNT receptors [18] signaling pathways. mTORC2 regulates the organization of the actin in the cytoskeleton, through various effectors, such as paxillin, Rho GTPases, or PKC-alpha. Additionally, mTORC2 controls diverse cellular processes, such as metabolism, survival, apoptosis, and growth, through the phosphorylation of various effectors; particularly, it directly phosphorylates two other protein kinases, Akt-Ser^473^ and serum- and glucocorticoid-induced protein kinase 1 (SGK1) [7].

It was described that the deregulation of the signaling pathways, both upstream and downstream of the mTORC1/mTORC2 is implicated in various diseases, such as aging, autoimmune disorders, neurodegenerative diseases, diabetes mellitus type II, obesity, and cancer [19]. Several epidemiological studies have related obesity to a high incidence of gastrointestinal cancers (esophageal, gastric, pancreatic, and colorectal) [20]. The mTOR signaling pathway plays an indispensable role in the regulation of adipose tissue functions, such as adipogenesis, thermogenesis, or lipid metabolism. Therefore, its modulation is important in the control of obesity, which is generated in circumstances of an abundance of nutrients [21].

Currently, different pharmacological options have been developed to inhibit mTOR, producing three generations of mTORC1 inhibitors, and their therapeutic efficacy and side effects are different in each case [22]. Both rapamycin and its chemical derivatives belong to the first generation of mTORC1 inhibitors. Despite being approved for the treatment of different types of cancer, they only cause stabilization of the disease, and not a tumor regression. That is, they behave as cytostatic and not as cytotoxic [22]. In addition, its continued clinical use causes numerous adverse effects, including suppression of the immune system, renal, dermatological, and hematological toxicity, and reduction of male fertility. Thus, in the current abundant clinical trials with rapalogs, their combination is sought, in lower doses, with other chemotherapy or radiotherapy agents to improve outcomes and overcome resistance [23]. The second generation of the mTOR inhibitors inhibits the catalytic kinase domain as ATP-competitive inhibitors and, therefore, controls the activity of both mTORC1 and mTORC2. Due to the structural similarities of the catalytic domain within the protein kinase superfamily [24], ATP-competitive inhibitors also block other kinases [25]. Additionally, various resistant mutations that interfere with drug-binding in the mTor, have been described in both rapalologs and kinase inhibitors [26]. A third, new-generation of mTor inhibitors is in experimental development to eliminate the resistance, and they consist of a bivalent drug that can bind simultaneously to the catalytic and regulator sites of the mTOR [26].

Considering the above-mentioned perspectives, the development of drugs targeted to mTOR, in gastrointestinal cancers associated with obesity or anti-aging, is an open field. Given the abundant structural information on the catalytic and regulator domains of mTOR, we have carried out an in silico study, to identify new potential inhibitors. We have used molecular docking techniques to select candidates among the compounds contained in the databases of marine natural products [27], Super Natural II [28], and ZINC natural products [29]. The compounds that showed the best binding scores were subsequently filtered using criteria from pharmacodynamics, pharmacokinetics, and toxicity properties. In a later step, we grouped the compounds with a structural similarity greater than or equal to 70%, to choose candidates to test in vitro that are representative of each cluster. We have tested the cytotoxic and inhibitory capacity of some compounds designed against the rapamycin binding site of the mTOR.

## 2. Results

### 2.1. Analysis of Compounds Docked to ATP and the Rapamycin Binding Sites of the mTOR Catalytic Domain

The molecular docking experiments aim to make a first selection of compounds with a low Gibbs free energy variation value (ΔG, kcal/mol), and then of potentially higher affinity, starting from a library of 484,527 compounds (including 14,442 from Marine Natural Products [27], 144,766 from ZINC natural products [29], and 325,319 from Super Natural II [28]). We have used all the structures available, thus far, to carry out the molecular docking at both of the ATP binding site (4JSN, 4JSP, 4JSV, 4JSX, 4JT5, 4JT6, 5WBU, 5WBY) and rapamycin (1FAP, 1NSG, 2FAP, 3FAP, 4FAP, 5GPG, 4DRH, 4DRI, 4DRJ) binding site. The data calculated after the molecular docking, presents, for each compound, up to twenty poses of that compound, bound to each of the explored binding site (ATP or rapamycin binding sites, see Figure 1) for all of the above-mentioned structures; likewise, ΔG was calculated. For each compound, we chose the pose with the lowest ΔG against each structure and expressed the values as the means and standard deviation. The calculated K_D_ (K_D_ = exp^ΔG/RT^) for compounds with ΔG ≤ −11 kcal/mol is in the subnanomolar range that was used as a threshold to filter the docking results [30,31,32]. When the results obtained for the rapamycin binding site were analyzed, 4.8% (692 compounds) of the marine natural product database had an average ΔG value less than or equal to −11 kcal/mol. More than 10% (33,635 compounds) of the Super Natural II had a ΔG ≤ −11 kcal/mol, and only 0.098% (142 compounds) of the ZINC natural product database met that condition. Only 0.25% (820 compounds) of the Super Natural II had a ΔG ≤ −13 kcal/mol. Analysis of the molecular docking data on the ATP binding site revealed a low number of compounds with a ΔG ≤ −11 kcal/mol, only twenty-three from the marine natural product database and a hundred and seven from the Super Natural II database. The average ΔG ± SD values for these compounds can be found in the supplementary material (Supplementary Tables S1–S5). Although we filtered the complete chemical library, considering the molecular docking data, we only discarded 89.4% of the compounds. Obviously, additional filters are needed until an approachable number of candidate compounds are obtained to be tested in vitro. The existence of a suitable ADMET (absorption, distribution, metabolism, excretion, and toxicity) profile for the initially selected compounds, will constitute the second filter, before proposing the compound candidates as the mTOR inhibitors.

**Figure 2:**
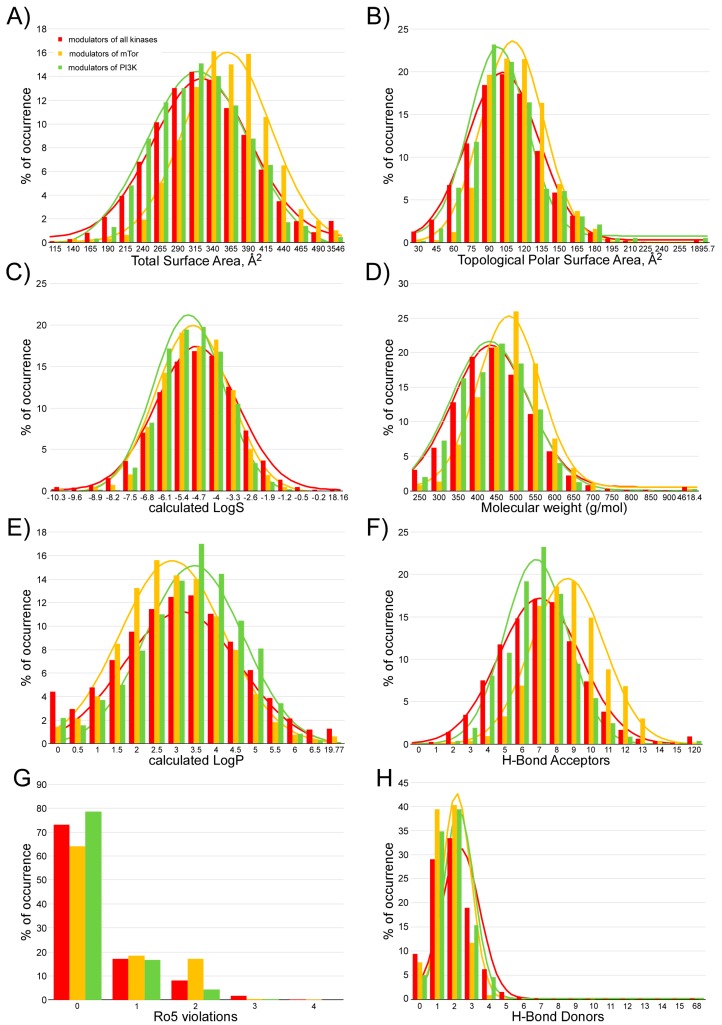

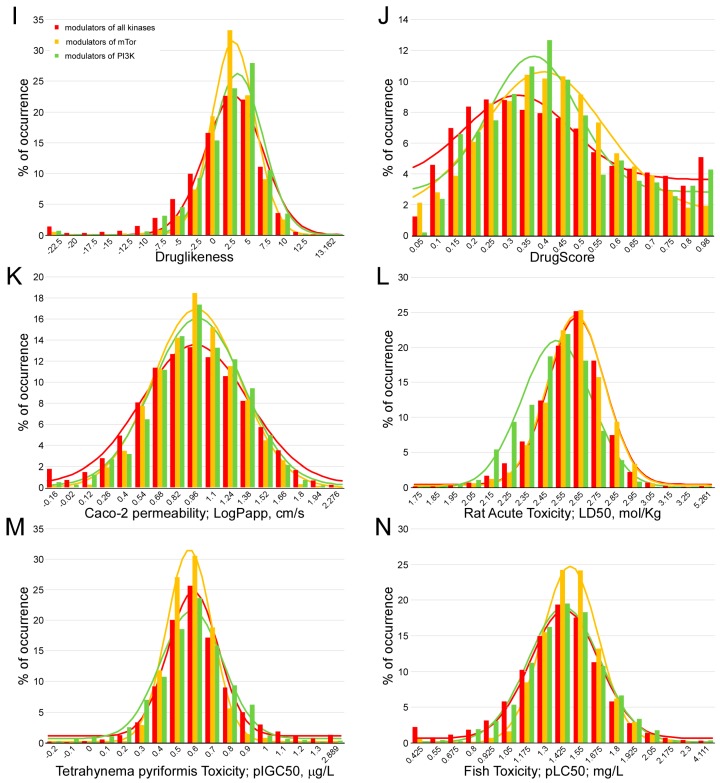
Analysis of the physicochemical and toxicological parameters of compounds that bind to all protein kinases, mTOR and PI3K from the bindingDB (https://www.bindingdb.org/) [33]. Gaussian distribution of the frequency of the calculated values for different parameters: Total surface area (**A**), topological polar surface area (**B**), calculated LogS (**C**), molecular weight (**D**), calculated logP (**E**), hydrogen-bond acceptors (**F**), violations of Lipinski’s rule of five (**G**), hydrogen-bond donors (**H**), drug-likeness (**I**), drug-score (**J**), Caco-2 permeability (**K**), rat acute toxicity (**L**), *Tetrahynema pyriformis* toxicity (**M**), and fish toxicity (**N**).

We analyzed different parameters of the ADMET profile of compounds that bind to different protein kinases—in many cases, drugs for clinical use—and for which experimental data were recorded in the bindingDB [33]. At the time of preparing Figure 2, the search terms ‘protein kinase’, ‘mTOR kinase’, and ‘PI3K’ generated 125,289, 4258, and 2883 results, respectively, in the bindingDB database (https://www.bindingdb.org/bind/as.jsp). The 3D chemical structure of these compounds was downloaded from the bindingDB; for each, the fourteen parameters represented in Figure 2, were calculated. As shown in Figure 2, thirteen of the fourteen analyzed parameters showed a Gaussian distribution at a frequency where 80–90% of the values of these parameters varied by the total surface area (A), topological polar surface area (B), calculated LogS (C), molecular weight (D), calculated logP (E), hydrogen-bond acceptors (F), violations of Lipinski’s rule of five (G), hydrogen-bond donors (H), drug likeness (I), drug score (J), calculated Caco-2 permeability (K), calculated rat acute toxicity (L), calculated *Tetrahynema pyriformis* toxicity (M), and calculated fish toxicity (N). The extreme values of these Gaussian distributions will be used as a screening filter for candidate compounds against the mTOR kinase activity. Only compounds that present the fourteen analyzed parameters, with values included between the extreme values of the Gaussian distributions, were selected after the application of this second filter. The minimum and maximum values of the fourteen parameters analyzed were as follows: 140 to 490 for the total surface area, 30 to 195 for the topological polar surface area, −13 to 0.2 for the calculated LogS, 250 to 750 for the molecular weight, −1 to 6.5 for the calculated logP, 0 to 13 for hydrogen-bond acceptors, 0 to 7 for hydrogen-bond donors, −10 to 10 for drug likeness, 0.05 to 0.8 for drug score, −0.16 to 1.8 for the calculated Caco-2 permeability (LogPapp, cm/s), 2 to 3.15 for the calculated rat acute toxicity (LD50, mol/kg), 0.1 to 1.1 for the calculated *Tetrahynema pyriformis* toxicity (pIGC50, µg/L), and 0.8 to 2 for the calculated fish toxicity (pLC50 mg/L). Up to two violations of Lipinski’s rules were admitted. After selecting only those compounds whose physicochemical and toxicological parameters mentioned above were within the limit values, the number of candidate compounds for mTOR inhibitors was considerably reduced. Only fifty-one compounds (6 from the marine natural product database [27] and 45 from the Super Natural II database [28]) were selected as candidates for inhibitors whose target was the ATP binding site of the mTOR (see Supplementary Table S6). The number of candidates, for inhibitors against the rapamycin binding site, was also significantly reduced, although their number was higher—445 (137 from the marine natural product database [27], 99 from ZINC natural products [29], and 209 from the Super Natural II database [28]) (see Supplementary Table S7).

**Figure 3:**
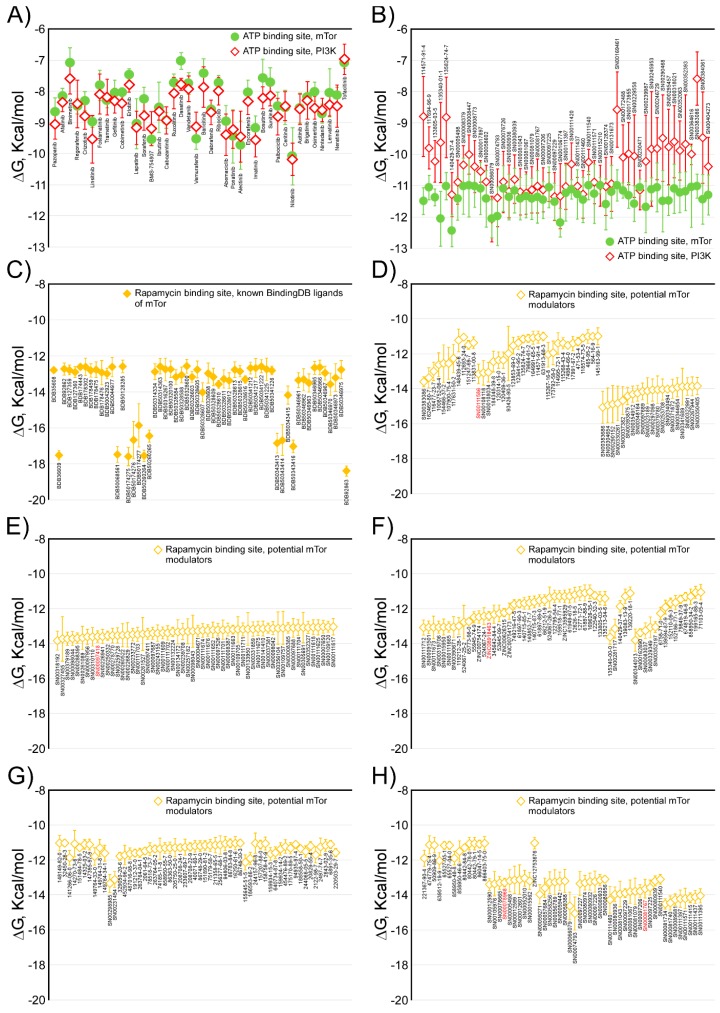

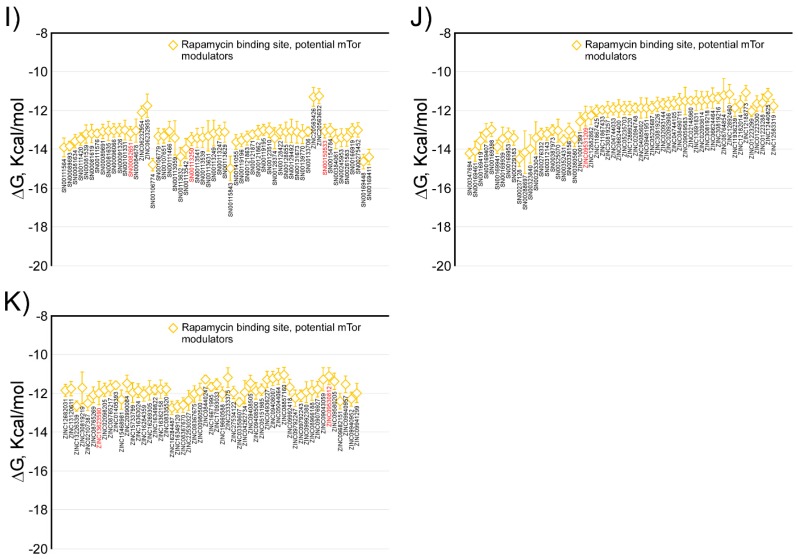
Comparison of the calculated Gibbs free energy variation (ΔG) for the selected inhibitor candidate compounds, against the ATP and rapamycin binding sites of the mTOR kinase. Panel (**A**) shows the calculated ΔG values for forty compounds approved by the FDA, for clinical use against various types of cancer against the ATP binding site. Panel (**B**) shows the ΔG values of the selected inhibitor candidate compounds against the ATP binding site of mTOR, and the same selected compounds against PI3K. Panel (**C**) shows the calculated ΔG values for fifty-six compounds registered in the bindingDB as mTOR kinase modulators. Panels (**D**–**K**) show the ΔG values of the selected inhibitor candidate compounds, against the rapamycin binding site of the mTOR. The common name (**A**), CAS number (for marine natural product compounds), Super Natural II, bindingDB (**C**), or ZINC names for all compounds, is indicated below each value, in black, except for the eleven compounds experimentally tested in this study (red color).

**Figure 4:**
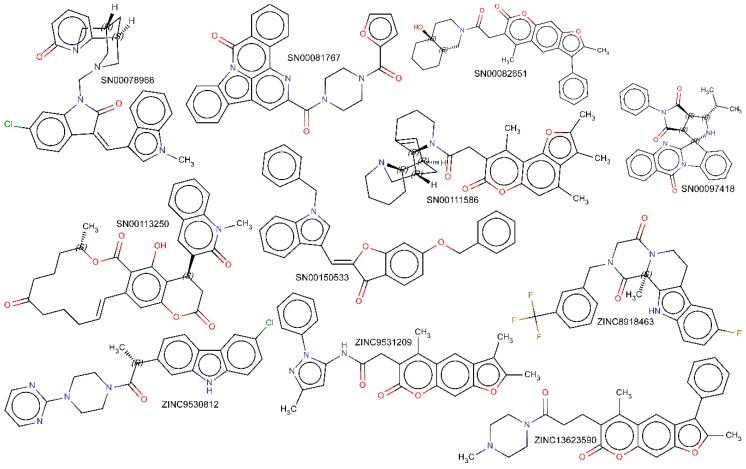
Molecular structure of compounds selected against the allosteric rapamycin binding site of mTOR, and tested experimentally. The name assigned to each compound by the Super Natural II [28] or ZINC database [29], in each case, is close to each structure.

Among the compounds proposed as candidates for mTOR inhibitors, high structural diversity was evident, judging by the high number of clusters in which they could be grouped—twenty-six compounds that targeted the ATP binding site and a hundred and six that targeted the rapamycin binding site (see Supplementary Tables S6 and S7). Figure 3 shows the Gibbs free energy variations calculated for the compounds docked to mTOR, against both the ATP and rapamycin binding sites, and are included in Supplementary Tables S6 and S7; in all cases, ΔG values were ≤ −11 kcal/mol. Figure 3A shows the calculated Gibbs free energy variations for the forty compounds approved by the Food and Drug Administration (FDA) as antineoplastic drugs, after molecular docking experiments against the ATP binding site of both mTOR and PI3K, which is a protein kinase highly related to the mTOR. From these data, two important conclusions were obtained. First, all of the compounds had an almost identical affinity to the ATP binding site of both protein kinases. Second, its affinity was relatively low (about −9 kcal/mol) compared with that of the candidate compounds included in Figure 3B, with ΔG values ≤ −11 kcal/mol. As shown in Figure 3B, among the compound candidates for mTOR inhibitors, we could distinguish both compounds whose ΔG were practically the same as that of the mTOR and PI3K, and compounds that differed by up to two kcal/mol, of which we would have expected high specificity in the inhibition of both protein kinases. In a recent article [34], it was shown that the compound “9m” showed low nanomolar activity against mTOR (IC_50_ = 7 nM) and greater selectivity over the related PIKK family kinases. The authors proposed a binding mode of compound “9m” with the ATP binding site of mTOR. On the other hand, Figure 3C shows the Gibbs free energy variations of compounds docked to the rapamycin binding site; these compounds have been described in the literature as modulators of mTOR and have been obtained from the bindingDB database [33] (4259 compounds, of which the 56 with minor ΔG are shown in the panel (C)). Here, we could distinguish two groups, a group of compounds with a ΔG over −12 kcal mol and another group with ΔG over −18 kcal/mol. The latter group included rapamycin and its chemical derivatives. The compounds selected as candidates for mTOR inhibitors (Figure 3D–K) have ΔG values that varied between −15 and −11 kcal/mol. Most of the compounds included in the databases, such as Super Natural II, ZINC natural products, and, especially, marine natural products, were not commercially available in adequate quantities and at an affordable price to carry out in vitro experiments. In fact, commercial availability was the “third selective filter” of compound candidates for mTOR inhibitors. The definition of clusters of compounds, with up to 70% identity in their structure, was a strategy that enabled the use of at least one representative compound of each cluster to test their inhibitory activity. We have not found examples in the literature of compounds, other than rapamycin or their chemical derivatives, that target the rapamycin binding site to inhibit the enzymatic activity of mTOR. Thus, it seemed conceptually innovative to target this regulatory site with non-rapamycin compounds. Additionally, the observation of its crystalline structure had revealed that its cavity was much larger than that of the ATP binding site. This would explain the greater number of candidate inhibitor compounds that we have found against the regulatory site of the mTOR. Consequently, we selected eleven commercially available compounds (see Figure 4) to test their in vitro inhibitory activity on mTOR kinase activity; each of these belonged to different clusters (except for cluster 7) with numerous compounds, of which we chose two compounds.

**Figure 5:**
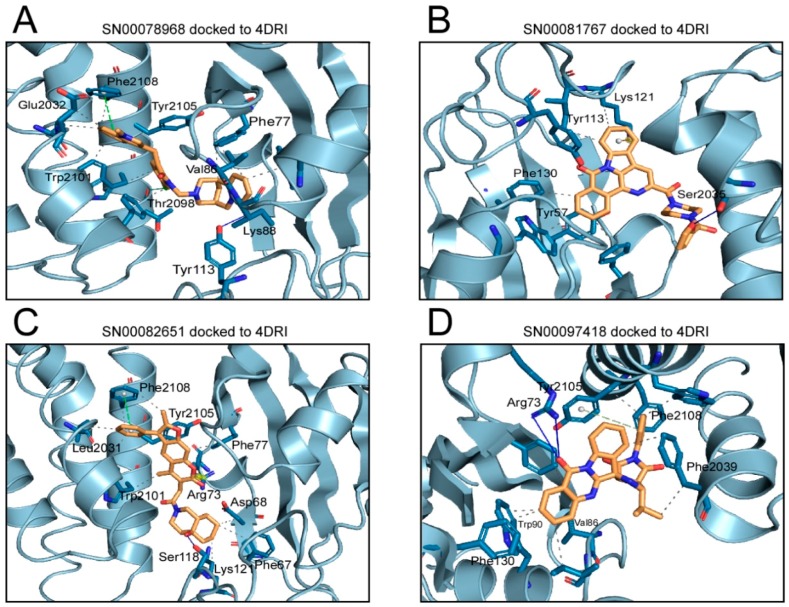

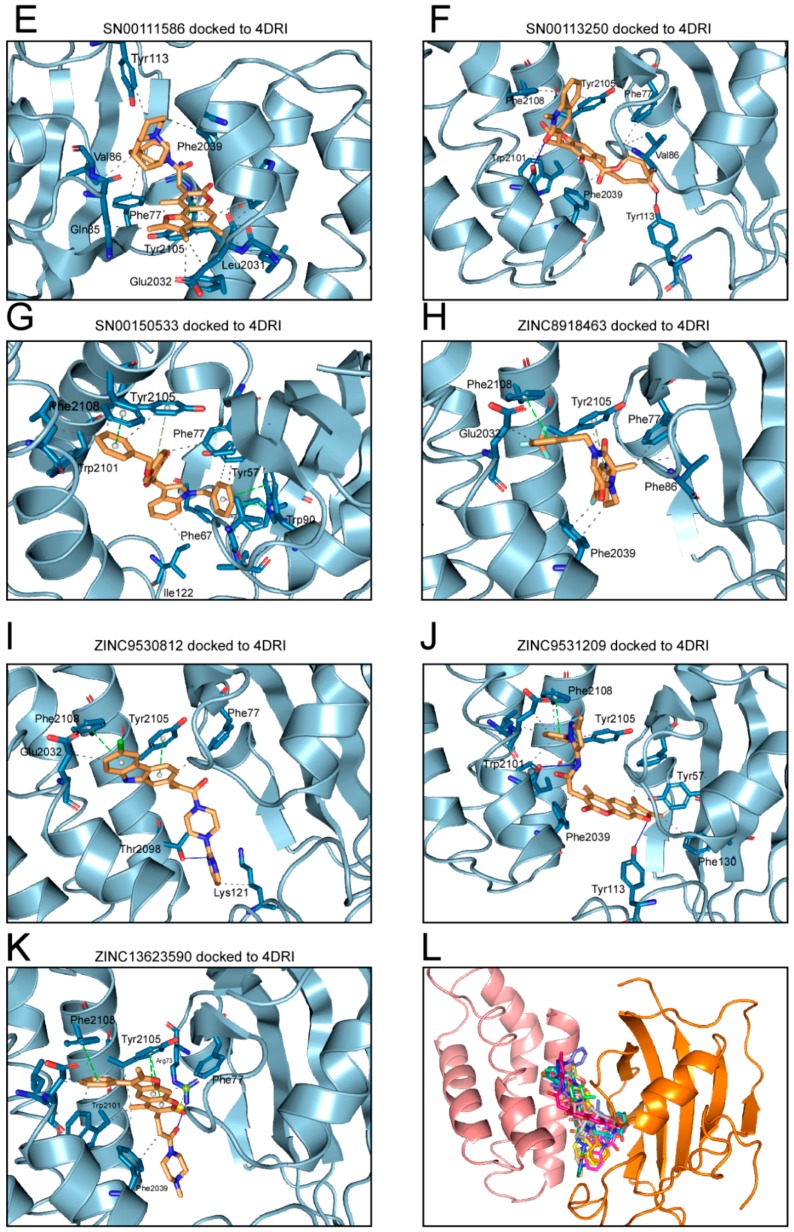
Molecular docking analysis for the 11 selected compounds against the allosteric rapamycin binding site of mTOR, showing the interacting residues of the binding site and each type of molecular interaction. Each panel of this figure has been prepared using the 4DRI structure of mTOR. For each compound docked to the protein, the pose with the lowest ΔG value has been shown. The interactions have been detected with the Protein–Ligand Interaction Profiler (FLIP) algorithm [35]. In each panel the compound analyzed is indicated. Panel L shows the best pose superimposed for each of the eleven inhibitor compounds. The full details of the molecular interactions are shown as supplementary information.

The Gibbs free energy variation (ΔG) is a representative value of the number and intensity of the atomic interactions, between the receptor (mTOR) and the docked compound [31]. The molecular docking of a protein target and the small ligand compounds predict the best interaction mode for a defined binding site. AutoDock/Vina, uses in its scoring function, the AMBER force field, which computes the terms of the contributions of van der Waals interactions, hydrogen bonding, electrostatic interactions, conformational entropy, and desolvation [30,31,32]. Figure 5 shows the molecular interactions between the amino acids of the rapamycin binding site and the inhibitor docked compounds. Details of the atoms involved in each type of interaction can be found in Supplementary Figure S1. In the interaction of each of the eleven compounds and the amino acids of the rapamycin binding site, hydrophobic interactions were predominant. In addition, most of the compounds that are able to establish hydrogen bonds and, in some cases, saline bridges, (SN00082651, ZINC13623590) were detected (Supplementary Figure S1).

### 2.2. Determination of the Cell Viability of HCT116 Cells after Treatment with Selected Inhibitor Candidate Compounds

**Figure 6:**
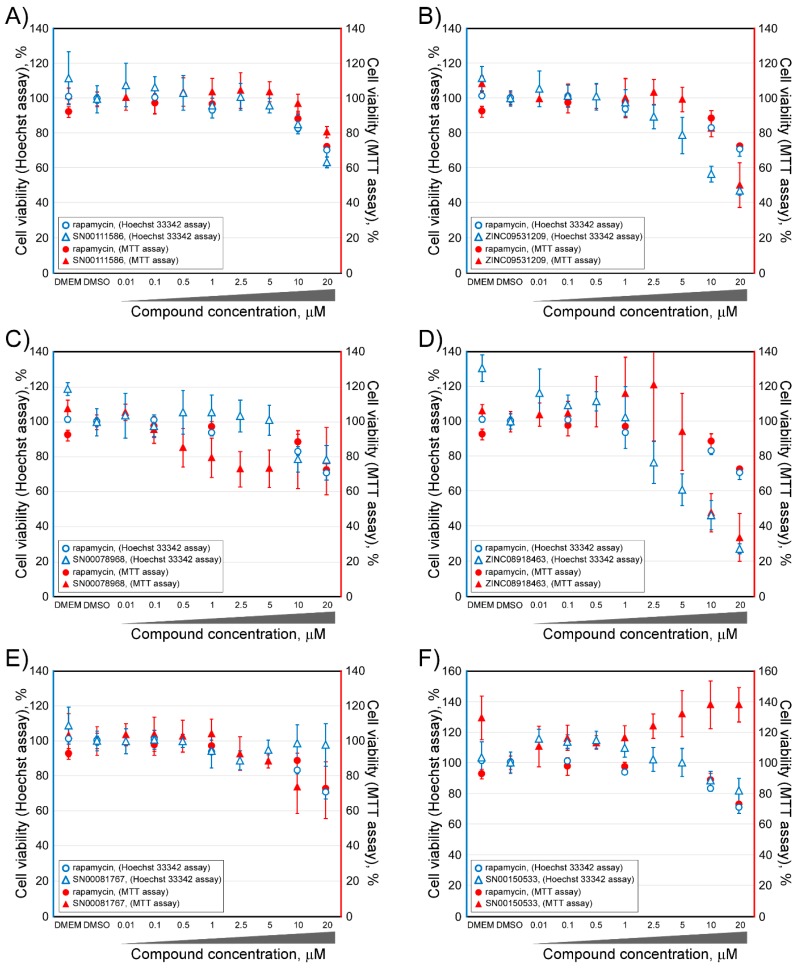

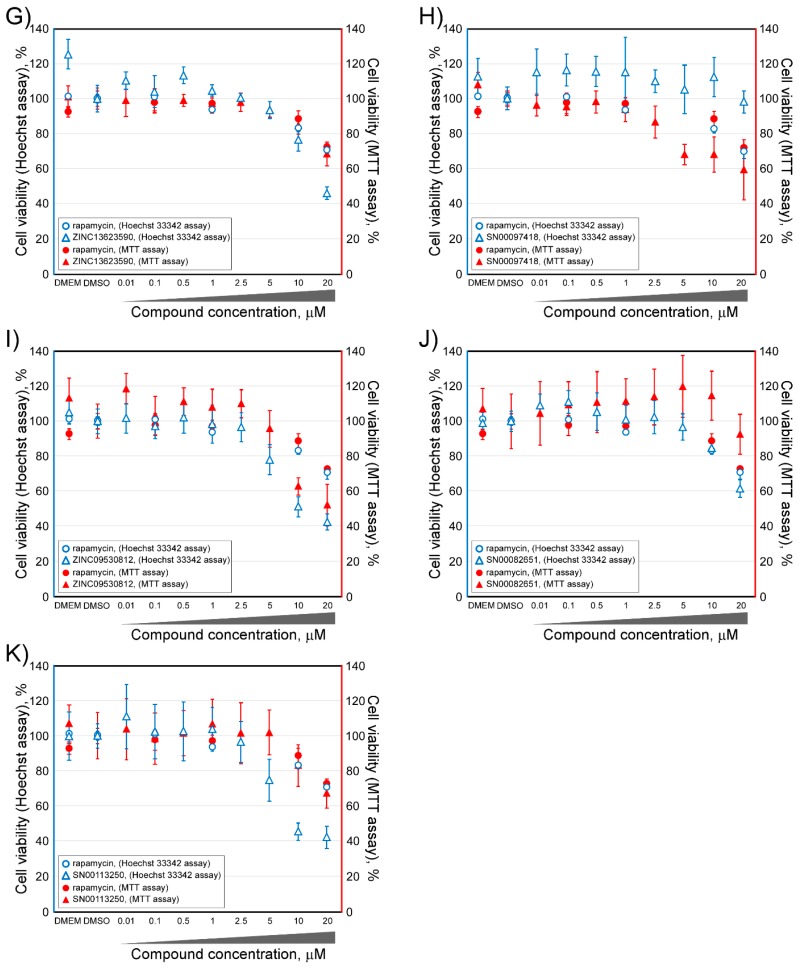
Viability of HCT116 cells after treatment with selected inhibitor candidate compounds, against the mTOR rapamycin binding site, as measured by both the MTT assay (symbols in red color) and counting Hoechst stained-nuclei (symbols in blue color). Each compound was always compared with rapamycin in parallel experiments. Each panel included a legend with the compounds analyzed and methodology followed (MTT or Hoechst stained-nuclei). The values were normalized with respect to media cultured only Dulbecco’s Modified Eagle Medium (DMEM).

Cellular cytotoxicity induced by the selected experimental compounds was evaluated by both the MTT cell viability assay and by counting the Hoechst-stained nuclei of the HCT116 human colon cancer cell line [36]. For the MTT cell viability assay (Figure 6), HCT116 cells were treated with a range of concentrations of each compound (0–20 μM). In parallel, treatments with equivalent amounts of the corresponding compound solvent Dimethyl sulfoxide (DMSO) were performed up to a maximum final concentration of 0.5% *v*/*v*. After 24 h, cell viability was determined by MTT as described in the “Materials and Methods” section [30]. Additionally, and to confirm that the compounds were not cytotoxic, a cell count was performed by taking microphotographs of the Hoechst-stained cells at 4x, using the 4′,6-Diamidino-2-phenylindole (DAPI) imaging filter cube [37] (see also Figure 6). As shown in Figure 6, HCT116 cells were sensitive to all compounds, including rapamycin, at a concentration of 20 µM. At this concentration, cell viability, followed by both techniques, was decreased by 20% to 80%, depending on each tested compound. If we observed the cell viability data in the presence of concentrations no greater than 10 µM, we can establish four groups of compounds. For the compound SN00078968, the maximum non-toxic concentration was 0.1 µM, that for compound SN00097418 was 1 µM, that for the compounds ZINC09531209, ZINC08918463, SN00081767, ZINC13623590, and ZINC09530812 was 5 µM, and, finally, that for the compounds SN00111586, SN00150533, SN00082651, and SN00113250, concentrations higher than 10 µM could not be used. Keeping in mind these maximum values of concentrations, for each compound that showed no cytotoxicity, they were then tested as potential mTOR inhibitors.

### 2.3. Determination of the Inhibitory Activity of the Selected Compounds against mTOR

**Figure 7:**
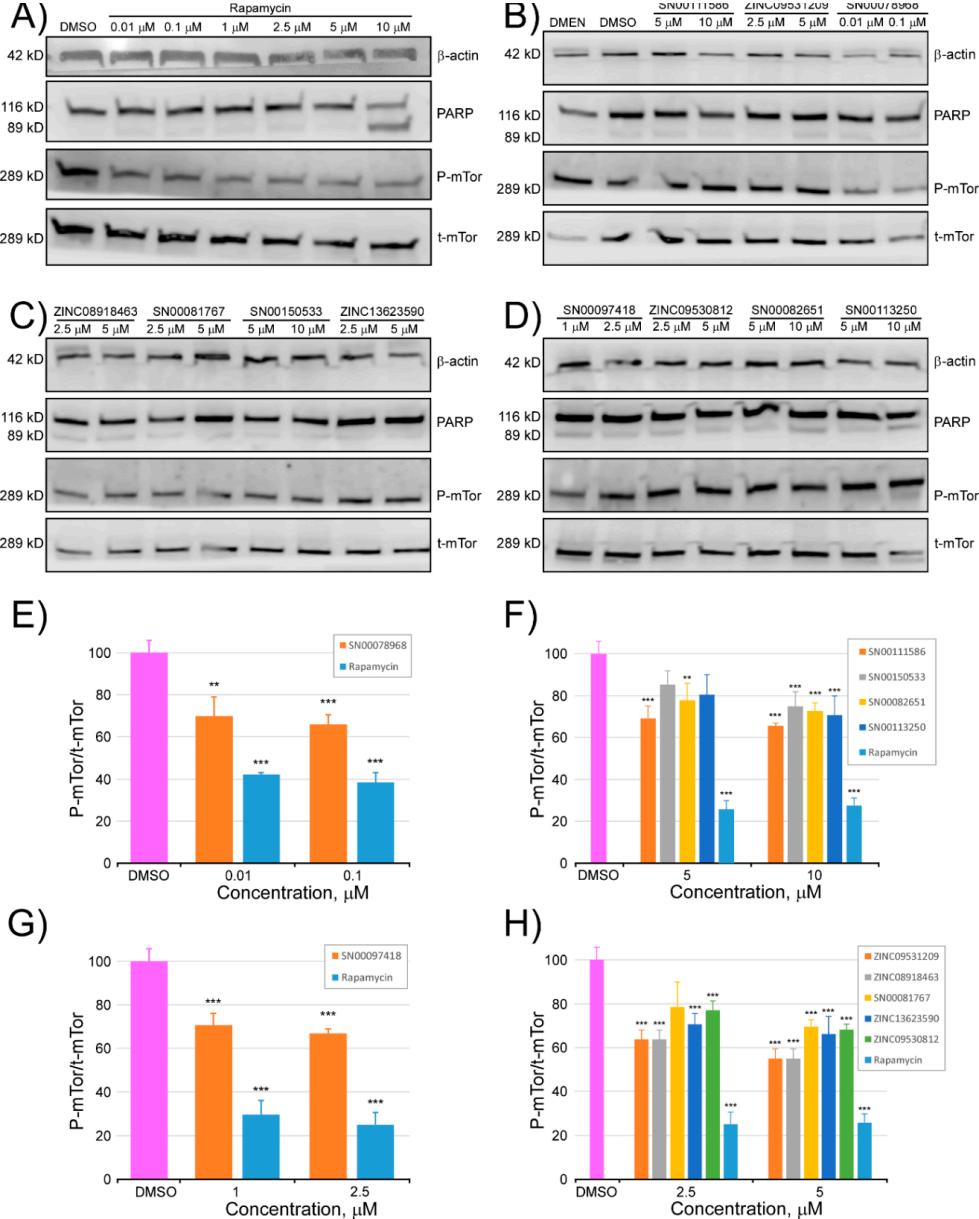
Inhibitory activity of selected compounds against the rapamycin binding site of mTOR. After incubation with different doses of all tested compounds and rapamycin or control medium (DMEM) and the compound vehicle (DMSO), for 24 h, HCT116 cells were lysed and subjected to Western blot analysis of total mTOR (t-mTor) and phosphorylated mTOR (P-mTor) antibodies (Panels (**A**–**D**)). Image quantification analysis of the Western blots presented as the ratio of P-mTor/t-mTor, in percentage, with respect to the control (DMSO) for the (**E**–**H**) panels. Levels of PARP (116 KDa) and cleaved-PARP (89 KDa) were detected by Western blotting. β-Actin was used as a loading control. The experiment was repeated four times with similar results. ** *p* < 0.01, *** *p* < 0.001 indicate significant differences compared with the control (DMSO).

Anti Phospho-mTOR (Ser2448) (D9C2) Rabbit mAb detects P-Ser2848 in mTOR. mTOR was phosphorylated at Ser2448 via the PI3K/Akt signaling pathway [38], and was autophosphorylated at Ser2481 [39]. Therefore, P-Ser2481 could be considered a marker of the autokinase activity of mTOR. Proteolysis of poly (ADP-ribose) polymerase (PARP), by caspase, into an 89-kD fragment was utilized as a marker for apoptosis [40,41]. As shown in Figure 7, while PARP cleavage was clearly detected in HCT116 cells treated with 10 µM rapamycin, which induced caspase-mediated apoptosis, only slight PARP cleavage was seen in the same cells treated with lower concentrations (see Figure 7A). As shown in Figure 7, the eleven compounds evaluated showed an inhibitory activity of mTOR, at the doses tested. The inhibition varied between 20% and 40%, with respect to the control, depending on each tested compound and dosage. All the compounds showed a percentage of inhibition, at the tested dose, representing approximately half of the percentage of inhibition that was achieved with the same dose of rapamycin.

### 2.4. Modulating Effect of Three Marine Extracts on the Activity of mTOR

**Figure 8:**
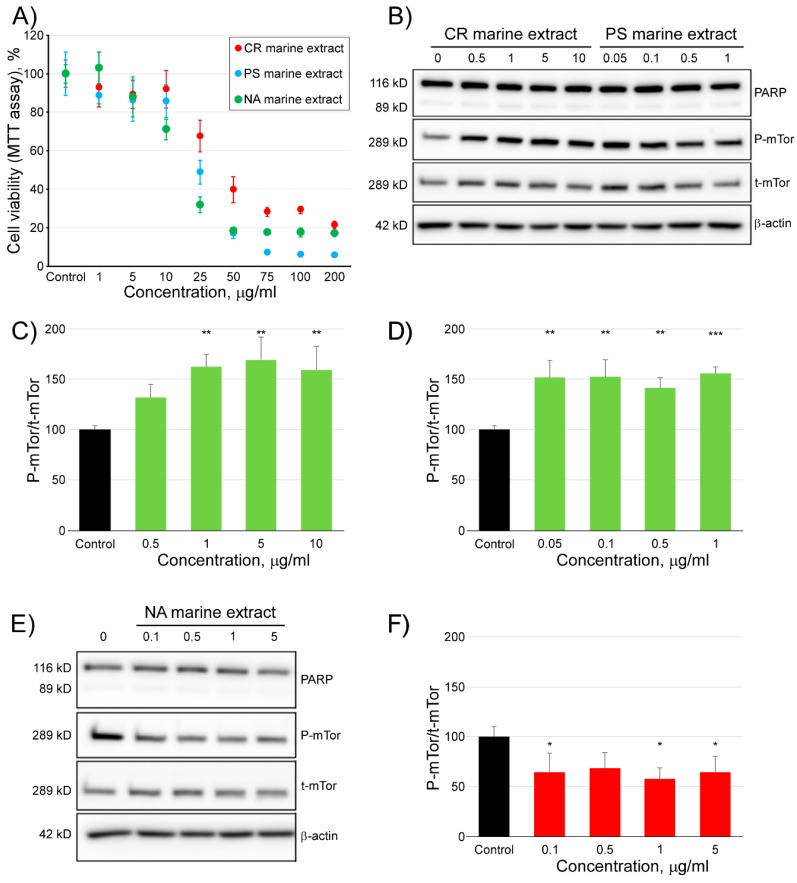
Analysis of the modulatory activity of three marine extracts on the activity of mTOR. Panel (**A**) shows the viability of HCT116 cells, after treatment with marine extracts against mTOR, followed by the MTT assay. After incubation with different doses of all the tested marine extracts or control [compound vehicle (DMSO)] for 24 h, HCT116 cells were lysed and subjected to the Western blot analysis of total mTOR (t-mTor) and phosphorylated mTOR (P-mTor) antibodies (Panels **B**,**E**). Image quantification analysis of the Western blots presented as the ratio P-mTor/t-mTor, in percentage, with respect to the control for the (Panels **C**,**D**,**F**). The levels of PARP (116 KDa) and cleaved-PARP (89 KDa) were also detected by Western blotting. β-actin was used as a loading control. The experiment was repeated four times with similar results. * *p* < 0.05, ** *p* < 0.01, and *** *p* < 0.001 indicate significant differences compared with the control.

In addition to the strategy presented thus far in our study, based on the use of mTOR structural information to target to its ATP or rapamycin binding sites with small molecules of known structure, we also used three marine extracts to check the modulatory activity of mTOR (Figure 8). As shown in Figure 8A, the viability in HCT116 cells did not decrease more than 10%, at concentrations of the extracts that did not exceed 10 µg/mL (CR extract), 1 µg/mL (PS extract), and 5 µg/mL (NA extract). Therefore, those maximum concentrations were used to determine their effect on the activity of mTOR. Considering the absence of the 89-kDa fragment of PARP in the blots presented in panel B (CR and PS extracts) and panel E (NA extract), none of the three marine extracts, used at the indicated doses, induced apoptosis. Surprisingly, two of the marine extracts (CR and PS extracts, panels 8C and 8D, respectively) dramatically activated the mTOR activity, increasing the P-mTor/t-mTor ratio by more than 50%, with respect to the control. By contrast, the NA extract (Figure 8F) showed an inhibitory effect on the mTOR activity of a similar magnitude (40% decrease with respect to the control), to that described above, for the compounds selected based on molecular docking and ADMET profiling experiments.

## 3. Discussion

Kinase proteins play a predominant regulatory role in cell biology because they can reversibly modify the activity of a protein, by increasing or decreasing its activity, which may involve the alteration of other biological activities. Additionally, the same kinases can be phosphorylated at different amino acids, and, in some cases, can be stimulators, while others can behave as inhibitors [24]. In the cell, protein kinases exist in the basal state and are only activated, when necessary, by very varied stimuli. Given their involvement in multiple functions of cell biology, the deregulation of their activity leads to important pathologies, such as cancer, aging, and inflammatory disorders. Thus, kinase proteins have become important pharmacological targets, and the development of inhibitory drugs is an important scientific and medical activity against human disease. Additionally, the constant appearance of mutations generates resistance to inhibitory drugs, representing a scientific challenge of the first order because of the health implications that this entails.

The resolved structure from the X-ray diffraction data of the catalytic domain of the protein kinases showed the existence of an N-ter lobe and another C-ter that formed a cleft which served as a binding site for ATP and Mg^2+^. Most kinase inhibitor drugs have this cavity as a target, and this supposes a problem of specificity when it is necessary to selectively modify the enzymatic activity of a particular protein kinase and probably contributes to the development of side effects. To support this assertion, we have carried out molecular docking experiments against the ATP binding site of the catalytic domain of 12 protein kinase and have found the best docking for forty drugs approved by the FDA, for use as antineoplastic drugs. Supplementary Figure S6 show the calculated ΔG (affinity) of the binding of these drugs to the ATP binding site. These values show that there are no significant differences for any of the molecules tested, with respect to their affinity for the ATP binding site in the twelve analyzed kinases. Thus, in our opinion, it would be more effective, from a therapeutic point of view, to develop inhibitors that target unique binding sites of each protein kinase. These regulatory sites, in many cases, have not yet been described. However, in the case of mTOR, the rapamycin binding site is known and allows specific inhibition of the mTORC1 complex, not the mTORC2 complex, which is insensitive to this drug.

Aiming to identify protein kinase targets that are different from the ATP binding site, we had, in our favor, that many kinases present modular domains (SH2, SH3) of temporary binding to other proteins or to themselves, especially, different sites of interaction with other proteins, that are involved in the formation of active complexes (this is the case of mTORC1 and mTORC2) or in the transmission of information in signaling cascades. In principle, the breakdown of any of these protein-protein interactions might be of therapeutic interest [42,43]. Although, in this work, we presented candidate compounds for mTOR inhibitors, targeted on the ATP binding site (Figure 3B and Supplementary Table S6), consistent with these ideas, we have only tested, in vitro, the inhibitory activity of those designed against the rapamycin binding allosteric site (Figure 3D–K and Supplementary Table S7).

In this study, we have presented up to a hundred and thirty-seven compounds of marine origin as candidates for inhibitors targeted to the rapamycin binding site. However, we could not test them all experimentally because of their lack of commercial availability. Although our group is making efforts to offer the scientific community information on the structures of marine natural compounds [27] (with more than 14,000 compounds) and there are also commercial databases available, such as MarinLit (http://pubs.rsc.org/marinlit) with more than 28,000 compounds in 2016, experimentation with pure compounds or enriched extracts is not easy. In our case, collaboration with the private sector allows access to the marine extracts of various species, for which we must characterize their molecular composition and determine their possible biological effects. This is the case of the three marine extracts presented in this study that has enabled the discovery of two extracts with activating capacity and one with inhibitory capacity on the mTOR protein kinase. Marine environments are largely unexplored in their biodiversity, however, undoubtedly, they harbor a huge source of potentially bioactive compounds that will allow the development of new products of interest in the pharmaceutical, food, cosmeceutical, and chemical industries. Despite these difficulties, eight marine natural products have been approved by the FDA as drugs, five of them against cancer, and twelve currently in different phases of clinical trials [27,44].

The computer-aided drug design methods accumulate a long experience in the development of small molecules with therapeutic action and reduce costs compared with high-throughput experimental screening approaches alone [45]. The experimental data that we showed in this study were consistent with this statement. In our case, we have explored a chemical library of 484,527 compounds and have proposed 491 compounds (51 against the ATP binding site and 445 against the rapamycin binding site) as candidate compounds inhibitors of mTOR. Among these candidates, we chose eleven to test their in vitro activity, and all of them showed the inhibitory capacity of mTOR. In our opinion, much of this success was due to the strict definition of the suitable filters to select the candidate compounds. In particular, developing a filter based on the statistical behavior of the calculated physico-chemical and toxicological parameters (ADMET profile, see Figure 2), for compounds registered in the bindingDB database [33] as protein kinase modulators, has made the difference between success and failure.

In our study we presented compounds capable of reducing in vitro the activity of mTOR by 20% and 40% (Figure 7), while rapamycin reached 60%, at similar concentrations and in the same cellular system tested. We wondered about the possibility of these compounds being potential candidates for further preclinical studies. mTORC1 is rapamycin sensitive and acts as a major checkpoint that coordinates the balance between cell growth and autophagy [46]. The control of mTOR activity has interest in cancer [1], obesity [21], and aging [46,47]. At the time of writing this manuscript the search term ‘rapamycin’ generated 1972 results in the National Institutes of Health clinical trials database (http://clinicaltrials.gov). These data are indicative of the clinical interest in this drug and anticipates abundant scientific information on its desirable and adverse effects. However, available clinical trials indicate that the blocking of mTORC1 with rapalogs shows considerable adverse effects. In addition, mTORC1 is ubiquitously expressed, reducing its effectiveness to focused therapies [22]. The use of rapalogs has demonstrated the existence of abundant side effects—dermatological, metabolic, renal, hematological, and respiratory toxicities, among others [48,49]. Trials in preclinical cancer models are promising but their clinical use does not generate the expected results [50]. Several reasons may explain the limitations of targeting mTORC1 in cancer therapy—resistant mutations of mTOR, activation of alternate proliferative signaling pathways, and intratumoral heterogeneity of mTOR activity, among others [22].

Given this scenario, the answer to the previous question is necessarily affirmative. In the same sense and in relation to the association between the hyperactivation of mTOR and aging, the development of new molecules that partially inhibit this kinase has an interest not only in basic science but also as possible therapeutic applications. The exhaustion of stem cells in their tissues of origin is considered the primary cause of aging. This is understood as the functional decline of cells/organs or the accumulation of damage, and is induced by various mechanisms, including the increased expression of inhibitory factors of the cell cycle or DNA damage, among others [51]. There is still much to learn about the molecular mechanisms that lead to aging, but the implication of changes in different signaling pathways with aging, is known and includes TGF-β, p38 MAPK, JAK/STAT, Delta/Notch, PI3K, and, of course mTOR, which represents a point of interconnection between these ways [47]. Various evidence has maintained that like caloric restriction, without reaching malnutrition, inhibition of mTORC1 can have similar beneficial effects on several pathologies related to age in rodents, and in some cases, humans [47]. The existing scientific evidence shows that inhibition of mTORC1 with rapamycin is currently the only pharmacological treatment that increases lifespan in all model organisms studied—invertebrates, yeasts or rodents [52]. Progress on this matter allows us to be optimistic about the possibilities of finding the mechanisms to delay human aging. Nevertheless, precautions must be taken because many side effects are associated with the use of rapalogs, thus, research on this direction should be promoted in the discovery of new mTOR inhibitors.

Finally, although the main purpose of our study was focused on finding mTOR inhibitors, we have also found two marine extracts with in vitro activating activity of this kinase (Figure 8B–D). In the literature, few chemical activators of mTOR have been described [53,54]. Oxidized low-density lipoprotein inhibits mTOR and induces autophagy and apoptosis in vascular endothelial cells, which play very critical roles in cardiovascular homeostasis [54]. CR and PS extracts increase the activity of mTOR in a proportion similar to compound 3BDO [54] and do not induce apoptosis. Our laboratory studies the characterization of its chemical composition and its effects on different cell types (manuscript in preparation).

## 4. Materials and Methods

### 4.1. Protein Structures of Human mTOR and PI3K Proteins and Chemical Libraries for Molecular Docking

By now, various crystal structures of the mTOR protein (UniProt P42345) have been resolved and deposited in the Protein Data Bank. This has enabled the exploration of both the ATP binding site (4JSN, 4JSP, 4JSV, 4JSX, 4JT5, 4JT6, 5WBU, 5WBY) and rapamycin binding site (1FAP, 1NSG, 2FAP, 3FAP, 4FAP, 5GPG, 4DRH, 4DRI, 4DRJ), in molecular docking experiments, in which we use all available structures. The virtual screening of ligands is more reliable if the flexibility of the receptor protein is considered. Thus, we have used multiple crystallographic receptor conformations to perform the molecular docking experiments [55]. The molecular docking experiments on the catalytic site of PI3K (UniProt P48736) have been carried out on the twenty-four (1E8Y, 6AUD, 4ANV, 3L54, 4WWO, 4ANW, 4HVB, 1E8Z, 3ENE, 4WWP, 3LJ3, 4GB9, 5G55, 4FUL, 5JHB, 2CHX, 2V4L, 3R7Q, 3T8M, 3ZVV, 5JHA, 3APC, 3APD, and 3ML9) resolved structures from X-ray data available in the Protein Data Bank.

Molecular docking experiments allow virtual screening of potentially modulating ligands of a given target because they prioritize certain compounds to be experimentally tested. Therefore, in these experiments, they require mining of long collections of chemical compounds. We have used a library of marine natural products (14,442 compounds) [27], ZINC natural products (144,766 compounds) [29] (http://zinc.docking.org/browse/catalogs/natural-products), and the SuperNatural II database (325,319 compounds) [28]. The 3D structures in the sdf format of all compounds tested are available at http://docking.umh.es/downloaddb (Chemical libraries: 2 for Super Natural II, 7 for marine natural products, and 8 for ZINC natural products) [30,31,32]. Mol2 files were converted to the pdbqt format, using the Python script “prepare_ligand4.py”, included into the AutodockTools-1.5.7.rc1 [56].

### 4.2. Molecular Docking Procedure

Before starting the molecular docking procedure, all structures of both the mTOR and the PI3K (see above for the PDB code) were submitted to geometric optimization, using the repair function of the FoldX algorithm [57]. To perform docking with AutoDock/Vina software v1.1.2 [58], the receptor and ligand structures were transformed to the pdbqt file format, which included atomic charges, atom-type definitions, and for the ligands, the topological information (rotatable bonds) [31,32]. A grid with dimensions of 23 × 23 × 23 points was centered to the co-crystallized ligands to ensure the coverage of the binding site of the structure. AutoDock/Vina was set up on a Linux cluster at lusitania2.cenits.es Linux cluster (Research, Technological Innovation and Supercomputing Center of Extremadura [CenitS]). AutoDock/Vina generated a conformer docked to the binding site in the protein, for each tested ligand, and calculated the Gibbs free energy variation of the binding process. Compounds with lower ΔG (kcal/mol) outperformed a first screening filter, as potential candidates for inhibitors [31,32].

### 4.3. Calculation of the Pharmacokinetic Parameters and Potential Toxicity Properties of the Inhibitor Candidates

Physicochemical parameters for the best docked compounds were calculated, as described previously [31,32], using DataWarrior v4.7.2 software (Allschwil, Switzerland) [59]. The ADMET (absorption, distribution, metabolism, excretion, and toxicity) properties were calculated using the admetSAR [60] web application and the DataWarrior v4.7.2 [59].

### 4.4. Chemical Compounds against mTOR Kinase Activity

The compounds with the SuperNatural II IDs SN00097418, SN00078968, SN00082651, SN00111586, SN00081767, SN00150533, SN00113250; and ZINC database IDs ZINC09531209, ZINC13623590, ZINC08918463, ZINC09530812 were purchased from the chemical supplier MolPort (supplier references MolPort-005-910-351, MolPort-000-853-506, MolPort-006-316-888, MolPort-000-849-049, MolPort-002-535-101, MolPort-002-649-206, MolPort-028-854-639, MolPort-000-853-173, MolPort-002-672-346, MolPort-002-523-849, MolPort-005-913-128, respectively), at https://www.molport.com/.

### 4.5. Preparation of Marine Extracts

Based on observations of inter and intra-specific competition in experimental aquariums and searches on bibliographic bases, four species of marine invertebrates were chosen for their potential as producers of compounds with anticancer potential. The selected species were composed of one soft coral (CR from *Carotalcyon* sp.), one nudibranch (NA from *Phyllidia varicosa*), and one holothurian (PS from *Pseudocholochirus violaceus*), and were obtained from the distributor company of marine species, Todo Pez S.L., Alicante, Spain.

### 4.6. Cell Culture and Treatment

Rapamycin and MTT were purchased from Sigma-Aldrich, St. Louis, MO, USA. High-glucose Dulbecco’s Modified Eagle’s Medium, penicillin-streptomycin, fetal bovine serum and 0.05x trypsin/ethylene diamine tetra acetic acid was purchased from Invitrogen Life Technologies (Carlsbad, CA, USA).

The human colorectal carcinoma cell line HCT116 was purchased from the American Type Culture Collection (ATCC, Manassas, VA, USA) and was maintained in Dulbecco’s modified Eagle’s medium (DMEM), supplemented with 10% heat inactivated fetal bovine serum (FBS), 100 U/mL of penicillin and 100 g/mL of streptomycin. Cells were incubated at 37 °C, in a humidified atmosphere, containing 5%/95% of CO_2_/air.

### 4.7. Cytotoxicity Assays

Cells were plated in 96-multiwell culture plates, at a density of 5 × 10^3^ cells/well. After 24 h of incubation with the crude extract, MTT was added at a final concentration of 0.5 mg/mL and was incubated for 3 h. Next, the medium was removed, and the formazan crystals were dissolved in DMSO. Absorbance was measured at 490 nm, subtracted at 670 nm in a microplate reader (SPECTROstar Omega, BMG Labtech, Offenburg, Germany). OD values were expressed in percentages relative to the control group, consisting of untreated cells. Cell viability was calculated by the formula: 100× (treated-cell absorbance/control-cell absorbance). All experiments were performed in triplicate, and the results were shown as the means with SD, calculated from three different experiments.

### 4.8. Quantification of the Total mTOR and Phosphor-mTOR Levels

After treatment, the cells were lysed with a lysis buffer—radioimmunoprecipitation assay buffer (RIPA Buffer) (BioRad Laboratories Inc., Madrid, Spain), for 60 min at −20 °C. The lysate was centrifuged at 13,200 rpm for 20 min; the protein concentration in the supernatant was spectrophotometrically determined using the Thermo Scientific Pierce Kit (Waltham, MA, USA) (BCA Protein assay kit), at 562 nm. Protein samples were diluted with the loading buffer (0.5 M Tris HCl at pH 6.8, 10% glycerol, 10% w/v SDS, 5% β2-mercaptoethanol, 0.05% w/v bromophenol blue) and then were boiled for 5 min. Proteins (30 μg/lane) and prestained standards (Thermo Fisher Scientific, Waltham, MA, USA) were loaded onto 4%–15% SDS precast polyacrylamide gels (BioRad Laboratories Inc., Madrid, Spain).

After electrophoresis, the resolved proteins were transferred from the gel to nitrocellulose membranes. A blotting buffer (20 mM Tris/150 mM glycine, pH 8, 20% v/v methanol) was used for the gel and for membrane saturation and blotting. The transblotted membranes were washed thrice with Tris buffered saline (TBS), containing 0.05% Tween 20 (TBST). After blocking with TBS containing 5% non-fat milk, for 60 min, the membranes were incubated with the appropriate primary antibodies (PARP Antibody #9542, Phospho-mTOR (Ser2448) (D9C2) XP Rabbit mAb #5536 and mTOR Antibody #2972) (Cell Signaling Technology Inc. Beverly, MA, USA) at 1:1000 dilution (with the exception of anti-β-actin antibody (Sigma-Aldrich, St. Louis, MO, USA), at 1:4000) in TBST–5% low fat milk at 4 °C overnight. The membranes were washed three times with TBST (15 min each) and then were incubated with the secondary antibody anti-human PARP, Phospho-mTOR and mTOR 1:2000, horseradish peroxidase (HRP)-conjugate (Cell Signaling Technology Inc., Barcelona, Spain), for 3 h. The bands were visualized by enhanced chemiluminescence (Licor; Lincoln, NE, USA).

### 4.9. Statistical Analysis

Values are represented as the mean ± standard deviation (SD). The values were subjected to statistical analysis (one-way ANOVA, and Tukey’s test for multiple comparisons/non-parametric approaches). The differences were considered to be statistically significant at *p* < 0.05. All analyses were performed using the Graph Pad Prism 6 (GraphPad Software, Inc., La Jolla, CA, USA). * *p* < 0.05, ** *p* < 0.01, and *** *p* < 0.001 on the bars, indicate statistically significant differences, compared to the control, unless otherwise stated. All cellular measurements were performed in four-fold, unless otherwise specified.

## Supplementary Materials

The following are available online at http://www.mdpi.com/1660-3397/16/10/385/s1, Supplementary Table S1: Physicochemical and toxicological parameters calculated for the selected compounds against the ATP binding site of mTOR based on molecular docking analysis. Supplementary Table S2: Physicochemical and toxicological parameters calculated for the selected compounds against the rapamycin binding site of mTOR based on molecular docking analysis. Supplementary Table S3: Molecular docking analysis for potential inhibitor compounds (candidate molecules) at the rapamycin binding site of mTOR kinase selected among Marine Natural Products. Supplementary Table S4: Molecular docking analysis for potential inhibitor compounds (candidate molecules) at the rapamycin binding site of mTOR kinase selected among Super Natural II database. Supplementary Table S5: Molecular docking analysis for potential inhibitor compounds (candidate molecules) at the rapamycin binding site of mTOR kinase selected among ZINC database natural products. Supplementary Table S6: Molecular docking analysis for potential inhibitor compounds (candidate molecules) at the ATP binding site of mTOR kinase selected among Marine Natural Products. Supplementary Table S7: Molecular docking analysis for potential inhibitor compounds (candidate molecules) at the ATP binding site of mTOR kinase selected among Super Natural II database. Supplementary Figure S1: Detailed description of the molecular interactions between the inhibitor compound and the rapamycin binding site in mTOR. Supplementary Figure S2: Comparison of the Gibbs free energy variation (ΔG, kcal/mol) for 40 compounds approved by the FDA for clinical use against various types of cancer, obtained by molecular docking experiments against the ATP binding site of 12 protein kinases.
