# Supplementary material for: New Mammalian Target of Rapamycin (mTOR) Modulators Derived from Natural Product Databases and Marine Extracts by Using Molecular Docking Techniques
Source: Mar Drugs. 2018 Oct 15;16(10):385. doi: 10.3390/md16100385 (PMC6213183; doi:10.3390/md16100385)
Supplement: Supplementary file 1 [file marinedrugs-16-00385-s001.pdf]

Supplementary Table S1: Molecular docking analysis for potential inhibitor compounds (candidate molecules) at the **rapamycin binding site** of **mTor** kinase selected among **Marine Natural Products** [1].

Marine natural products	ΔG, mean±S.D.	Marine natural products	ΔG, mean±S.D.	Marine natural products	ΔG, mean±S.D.
40663-80-7	-13.89±0.65	184348-39-8	-12.46±0.59	115267-16-8	-12.06±0.71
681260-42-4	-13.74±0.53	55945-74-9	-12.45±0.31	114820-24-5	-12.04±0.60
139975-57-8	-13.72±0.60	116-30-3	-12.42±0.26	121038-34-4	-12.04±1.57
104196-68-1	-13.71±0.64	126622-64-8	-12.41±0.23	114820-28-9	-12.02±0.32
133625-26-0	-13.70±0.43	147362-37-6	-12.40±0.78	639512-19-9	-12.02±0.45
681260-09-3	-13.69±0.44	16891-85-3	-12.40±0.83	64687-85-0	-12.02±0.64
178115-90-7	-13.66±0.79	120314-15-0	-12.38±0.47	890041-67-5	-12.02±0.46
135340-00-0	-13.57±0.40	120409-36-1	-12.38±0.47	29953-50-2	-12.00±0.69
112663-92-0	-13.50±0.73	131204-29-0	-12.38±0.41	36011-19-5	-12.00±0.38
152845-74-4	-13.43±0.50	524067-24-1	-12.38±0.47	93426-90-5	-12.00±1.56
162465-80-7	-13.39±0.58	160796-24-7	-12.37±0.46	175673-58-2	-11.99±0.25
211686-51-0	-13.39±0.32	75598-52-6	-12.37±0.53	150079-95-1	-11.98±0.38
159736-39-7	-13.38±0.57	50335-03-0	-12.35±0.52	798557-98-9	-11.98±0.33
139975-58-9	-13.32±0.41	70022-71-8	-12.35±0.30	79858-77-8	-11.98±0.71
94354-98-0	-13.30±0.15	122780-90-9	-12.33±0.36	864685-94-9	-11.98±0.39
14729-29-4	-13.18±0.73	65745-47-3	-12.33±0.36	173792-58-0	-11.96±0.68
214899-21-5	-13.12±0.77	149064-34-6	-12.32±0.63	849215-95-8	-11.95±0.64
116477-23-7	-13.02±0.53	179733-14-3	-12.32±0.36	191-24-2	-11.94±0.64
133613-77-1	-13.01±0.60	212502-88-0	-12.31±0.46	156953-91-2	-11.93±0.91
79067-75-7	-13.01±0.69	117631-50-2	-12.28±0.81	72509-61-6	-11.93±0.73
135340-01-1	-13.00±0.32	184885-91-4	-12.28±0.47	76915-24-7	-11.93±0.42
119212-28-1	-12.94±0.47	188111-70-8	-12.28±0.69	81657-79-6	-11.93±0.39
149764-32-9	-12.92±0.86	3148-09-2	-12.28±0.60	120154-96-3	-11.92±0.32
134458-00-7	-12.90±0.48	345642-84-4	-12.28±0.52	78111-17-8	-11.92±0.26
179733-15-4	-12.90±0.49	74608-63-2	-12.28±0.42	325690-73-1	-11.90±0.94
214767-82-5	-12.89±0.48	81657-78-5	-12.28±0.37	6853-99-2	-11.90±0.52
156953-87-6	-12.88±0.95	848656-06-4	-12.27±0.63	119539-75-2	-11.89±0.49
50335-04-1	-12.88±0.23	120963-61-3	-12.26±0.53	12794-85-3	-11.88±0.38
55544-35-9	-12.88±0.34	862892-28-2	-12.25±0.54	139594-87-9	-11.88±0.39
105418-77-7	-12.84±0.34	221163-30-0	-12.23±0.34	191212-38-1	-11.88±0.27
125282-13-5	-12.82±0.52	52645-09-7	-12.23±0.47	221367-90-4	-11.88±0.46
125302-26-3	-12.82±0.52	126297-39-0	-12.22±1.16	863116-48-7	-11.88±0.41
133056-09-4	-12.79±0.90	6758-71-0	-12.22±0.35	186593-86-2	-11.87±0.35
144587-58-6	-12.77±0.39	114820-30-3	-12.21±0.31	28097-03-2	-11.87±0.50
155944-26-6	-12.74±0.35	114820-25-6	-12.19±0.40	67463-79-0	-11.87±0.52
524067-25-2	-12.72±0.95	233607-72-2	-12.19±0.62	858950-48-8	-11.87±0.39
133056-07-2	-12.71±0.50	83481-23-6	-12.17±0.33	129033-05-2	-11.86±1.13
65773-98-0	-12.70±0.11	123048-14-6	-12.16±0.25	84773-08-0	-11.85±0.88
80375-18-4	-12.65±0.54	88840-01-1	-12.16±0.36	141266-06-0	-11.84±0.41
1008752-06-4	-12.64±0.69	123000-07-7	-12.14±0.24	140429-37-4	-11.83±0.32
823808-55-5	-12.63±0.19	171674-92-3	-12.14±0.83	150050-13-8	-11.83±0.54
123303-94-6	-12.62±0.29	88840-02-2	-12.14±0.24	168569-18-4	-11.82±0.33
123303-95-7	-12.62±0.30	88903-69-9	-12.14±0.24	185801-42-7	-11.81±0.51
50-76-0	-12.62±1.02	64726-84-7	-12.12±0.55	104759-19-5	-11.80±0.30
64421-20-1	-12.62±0.61	148371-08-8	-12.10±0.44	105305-54-2	-11.80±0.60
128229-64-1	-12.61±0.34	155645-51-5	-12.10±0.43	159813-67-9	-11.80±0.34

144587-57-5	-12.58±0.47	158734-27-1	-12.10±0.42	191212-36-9	-11.80±0.24
154466-37-2	-12.57±1.07	124689-65-2	-12.09±0.60	87164-33-8	-11.80±0.28
107900-75-4	-12.56±0.91	147362-39-8	-12.09±0.30	168482-38-0	-11.79±0.14
212502-87-9	-12.56±0.56	186593-83-9	-12.08±0.48	211239-56-4	-11.79±0.37
6377-18-0	-12.55±0.24	706784-72-7	-12.08±0.33	114820-26-7	-11.78±0.38
127687-08-5	-12.47±0.23	708255-98-5	-12.08±0.87	156953-85-4	-11.78±0.53
240480-95-9	-12.47±0.45	35906-51-5	-12.07±0.26	87532-26-1	-11.78±0.13
168569-15-1	-12.46±0.60	5035-30-3	-12.07±0.21	82915-89-7	-11.77±0.51
858950-44-4	-11.77±0.58	149764-34-1	-11.59±0.45	81275-81-2	-11.48±0.32
108195-61-5	-11.76±0.26	134887-25-5	-11.58±0.37	866403-74-9	-11.48±0.34
149764-33-0	-11.76±0.50	140447-22-9	-11.58±0.41	272118-06-6	-11.47±1.02
329050-20-6	-11.75±0.23	140715-87-3	-11.58±0.33	66536-82-1	-11.47±0.57
78798-08-0	-11.75±0.52	151606-24-5	-11.58±0.26	73538-57-5	-11.47±0.25
80860-53-3	-11.75±0.29	151606-43-8	-11.58±1.18	866403-73-8	-11.47±0.36
116229-58-4	-11.74±1.21	191212-37-0	-11.57±0.22	87532-32-9	-11.47±0.33
80928-52-5	-11.73±1.11	329050-23-9	-11.57±0.49	121250-35-9	-11.46±0.52
172854-78-3	-11.72±0.54	865369-07-9	-11.57±0.55	136024-80-1	-11.46±0.85
209169-58-4	-11.72±0.58	93474-14-7	-11.57±0.12	184679-29-6	-11.46±0.89
749216-47-5	-11.72±0.27	112455-84-2	-11.56±0.47	2061-64-5	-11.46±0.19
149444-92-8	-11.70±0.49	145163-96-8	-11.56±0.34	329050-22-8	-11.45±0.42
156310-18-8	-11.70±0.77	263764-04-1	-11.56±0.26	472-70-8	-11.45±0.37
81720-10-7	-11.70±0.22	27065-95-8	-11.55±0.26	64907-26-2	-11.45±0.52
852872-92-5	-11.70±0.44	51744-55-9	-11.55±0.41	7488-99-5	-11.45±0.46
159334-35-7	-11.69±0.49	640734-87-8	-11.55±0.33	78798-30-8	-11.45±0.73
175702-36-0	-11.69±0.34	123853-69-0	-11.54±0.48	84582-62-7	-11.45±0.26
199600-36-7	-11.68±0.37	220503-29-7	-11.54±0.23	114836-87-2	-11.44±0.61
452934-96-2	-11.68±0.55	224577-34-8	-11.54±0.50	134981-78-5	-11.43±0.37
4936-10-1	-11.68±0.28	171784-09-1	-11.53±0.36	518-06-9	-11.43±0.28
50676-98-7	-11.68±0.27	17608-76-3	-11.53±0.31	78173-92-9	-11.43±0.69
61897-90-3	-11.68±0.23	211358-70-2	-11.53±0.35	78340-85-9	-11.43±0.34
65556-58-3	-11.68±0.33	432-68-8	-11.53±0.44	79849-37-9	-11.43±0.23
91097-16-4	-11.68±0.63	52238-31-0	-11.53±0.27	870095-34-4	-11.43±0.26
2122-98-7	-11.67±0.46	53402-18-9	-11.53±0.26	96391-64-9	-11.43±0.12
78111-14-5	-11.67±0.23	74984-66-0	-11.53±0.14	105118-23-8	-11.42±0.41
94705-97-2	-11.67±0.67	84323-27-3	-11.53±0.22	114995-72-1	-11.42±0.62
140715-85-1	-11.66±0.34	85199-91-3	-11.53±0.49	123941-58-2	-11.42±0.27
147716-81-2	-11.66±1.64	863548-84-9	-11.53±0.39	17991-67-2	-11.42±0.41
890041-68-6	-11.66±0.42	126622-63-7	-11.52±0.38	208927-15-5	-11.42±0.43
104758-18-1	-11.64±0.52	159518-76-0	-11.52±0.56	77617-71-1	-11.42±0.23
152110-09-3	-11.64±0.28	16910-32-0	-11.52±0.42	77739-71-0	-11.42±0.69
2465-11-4	-11.64±0.35	445430-63-7	-11.52±0.56	79781-66-1	-11.42±0.24
88840-00-0	-11.64±0.51	514-78-3	-11.52±0.16	115268-43-4	-11.41±0.32
140852-71-7	-11.63±0.36	676339-97-2	-11.52±0.17	122540-31-2	-11.41±0.21
152213-67-7	-11.63±0.20	139933-46-3	-11.51±0.50	1406-16-2	-11.41±0.40
6673-66-1	-11.63±0.19	14984-66-8	-11.51±0.42	224577-31-5	-11.41±0.57
865308-60-7	-11.63±0.28	171370-52-8	-11.51±0.24	160472-96-8	-11.40±1.67
1403-36-7	-11.62±0.91	174630-11-6	-11.51±0.68	176328-50-0	-11.40±0.55
152186-77-1	-11.62±0.40	224577-33-7	-11.51±0.56	210761-24-3	-11.40±0.41
159934-15-3	-11.62±0.36	116237-60-6	-11.50±0.55	874383-70-7	-11.40±0.39
19698-66-9	-11.62±0.26	151247-69-7	-11.50±0.25	88095-77-6	-11.40±0.64
199600-37-8	-11.62±0.41	172854-77-2	-11.50±0.48	88357-79-3	-11.40±0.39

4626-00-0	-11.62±0.62	61897-89-0	-11.50±0.32	95189-16-5	-11.40±0.17
141968-27-6	-11.60±0.66	78306-12-4	-11.50±0.48	122970-13-2	-11.39±0.26
174063-81-1	-11.60±0.40	84821-12-5	-11.50±0.37	12794-84-2	-11.39±0.43
188111-69-5	-11.60±0.55	858950-46-6	-11.50±0.60	154563-79-8	-11.39±0.34
23518-98-1	-11.60±0.26	150050-12-7	-11.49±0.64	28380-31-6	-11.39±0.34
37976-88-8	-11.60±0.23	217449-19-9	-11.49±0.34	103614-76-2	-11.38±0.98
452934-97-3	-11.60±0.36	153212-84-1	-11.48±0.75	113831-00-8	-11.38±0.33
487016-98-8	-11.60±0.32	210761-25-4	-11.48±0.44	123641-95-2	-11.38±0.38
52212-86-9	-11.60±0.61	244066-05-5	-11.48±0.32	135213-04-6	-11.38±0.40
53755-09-2	-11.60±0.47	306971-11-9	-11.48±0.27	156953-93-4	-11.38±0.96
70214-98-1	-11.60±0.78	66212-51-9	-11.48±0.31	209167-28-2	-11.38±0.32
393828-32-5	-11.38±0.40	25747-44-8	-11.29±0.41	50364-22-2	-11.23±0.48
63693-18-5	-11.38±0.31	101527-89-3	-11.28±0.45	51446-63-0	-11.23±0.32
77658-95-8	-11.38±0.42	132438-14-3	-11.28±0.41	52423-28-6	-11.23±0.73
133805-03-5	-11.37±0.37	149764-31-8	-11.28±0.48	874193-61-0	-11.23±0.46
14050-62-5	-11.37±0.35	151232-83-6	-11.28±0.32	122540-32-3	-11.22±0.20
37933-92-9	-11.37±0.38	219832-37-8	-11.28±0.63	184885-90-3	-11.22±0.65
452934-95-1	-11.37±0.46	487016-96-6	-11.28±0.78	208708-22-9	-11.22±0.23
66648-53-1	-11.37±0.35	674819-46-6	-11.28±0.23	222960-90-9	-11.22±0.53
74185-04-9	-11.37±0.26	69081-88-5	-11.28±0.34	2497-74-7	-11.22±0.49
80680-43-9	-11.37±0.46	80442-84-8	-11.28±0.18	432-70-2	-11.22±0.96
103005-20-5	-11.36±0.73	823815-00-5	-11.28±0.42	102778-12-1	-11.21±0.41
134887-27-7	-11.36±0.56	85733-69-3	-11.28±0.25	120881-21-2	-11.21±0.64
219832-35-6	-11.36±0.36	858950-34-2	-11.28±0.43	144686-42-0	-11.21±0.22
22453-06-1	-11.36±0.31	858950-40-0	-11.28±0.28	174024-97-6	-11.21±0.23
24041-68-7	-11.36±0.26	858950-55-7	-11.28±0.31	193816-71-6	-11.21±0.40
78518-73-7	-11.35±0.36	86363-50-0	-11.28±0.33	23637-31-2	-11.21±0.33
79234-80-3	-11.35±0.71	108943-00-6	-11.27±0.47	110012-18-5	-11.20±0.16
823815-01-6	-11.35±0.45	122795-54-4	-11.27±0.35	163136-05-8	-11.20±0.33
852872-93-6	-11.35±0.52	137415-10-2	-11.27±0.40	272458-31-8	-11.20±0.17
114550-75-3	-11.34±0.37	149444-91-7	-11.27±0.99	356566-82-0	-11.20±0.27
127-22-0	-11.34±0.30	151484-78-5	-11.27±0.17	454476-89-2	-11.20±0.39
163136-04-7	-11.34±0.44	153723-34-3	-11.27±0.83	50364-21-1	-11.20±0.18
290353-68-3	-11.34±0.19	2130-17-8	-11.27±0.69	54369-11-8	-11.20±0.40
127687-07-4	-11.33±0.34	23983-43-9	-11.27±0.28	73538-56-4	-11.20±0.22
134887-26-6	-11.33±0.52	306971-07-3	-11.27±0.33	79-63-0	-11.20±0.41
144-68-3	-11.33±0.32	78370-84-0	-11.27±0.25	85733-68-2	-11.20±0.23
159934-14-2	-11.33±0.35	84323-29-5	-11.27±0.36	101509-61-9	-11.19±0.41
174232-38-3	-11.33±0.26	96157-95-8	-11.27±0.23	110012-19-6	-11.19±0.25
263764-05-2	-11.33±0.20	105377-92-2	-11.26±0.26	124417-91-0	-11.19±0.11
31272-50-1	-11.33±0.29	120853-13-6	-11.26±0.30	147641-71-2	-11.19±0.31
364344-14-9	-11.33±0.73	134887-31-3	-11.26±0.59	148839-03-6	-11.19±0.43
524067-26-3	-11.33±0.59	139083-13-9	-11.26±0.41	199600-38-9	-11.19±0.52
84711-17-1	-11.33±0.34	159518-77-1	-11.26±0.32	283176-13-6	-11.19±0.32
858950-52-4	-11.33±0.60	184885-07-2	-11.26±0.34	107503-09-3	-11.18±0.42
154563-80-1	-11.32±0.30	194856-36-5	-11.26±0.22	133161-20-3	-11.18±0.36
188558-50-1	-11.32±0.16	205750-25-0	-11.26±0.52	156953-89-8	-11.18±0.99
35115-60-7	-11.32±0.53	208708-24-1	-11.26±0.19	260062-88-2	-11.18±0.24
471-53-4	-11.32±0.24	481-17-4	-11.25±0.39	40071-60-1	-11.18±0.33
74185-11-8	-11.32±0.60	571176-94-8	-11.25±0.23	40600-59-7	-11.18±0.28
116237-58-2	-11.31±0.69	61949-67-5	-11.25±0.39	7542-45-2	-11.18±0.28

194427-76-4	-11.31±0.41	63121-06-2	-11.25±0.39	782491-78-5	-11.18±0.15
106009-90-9	-11.30±0.46	70214-92-5	-11.25±0.60	78285-84-4	-11.18±0.41
124508-52-7	-11.30±0.40	85249-28-1	-11.25±0.35	78285-85-5	-11.18±0.23
179730-36-0	-11.30±0.31	118964-36-6	-11.24±0.32	80925-08-2	-11.18±0.41
209167-27-1	-11.30±0.32	148439-45-6	-11.24±0.65	82660-61-5	-11.18±0.30
256505-53-0	-11.30±0.42	26605-16-3	-11.24±0.39	862200-49-5	-11.18±0.19
37976-89-9	-11.30±0.28	114820-27-8	-11.23±0.34	11052-32-7	-11.17±0.95
445430-65-9	-11.30±0.36	115982-22-4	-11.23±0.31	12626-18-5	-11.17±0.42
852469-37-5	-11.30±0.40	124417-90-9	-11.23±0.29	126-29-4	-11.17±0.32
90077-71-7	-11.30±0.51	176704-07-7	-11.23±0.35	127709-45-9	-11.17±0.47
125329-09-1	-11.29±0.46	195063-95-7	-11.23±0.42	135824-74-7	-11.17±0.22
164081-03-2	-11.29±0.33	20780-37-4	-11.23±0.39	137443-15-3	-11.17±0.42
169564-96-9	-11.29±0.50	233607-69-7	-11.23±0.45	143621-75-4	-11.17±0.39
175170-89-5	-11.29±0.32	40772-12-1	-11.23±0.18	144686-41-9	-11.17±0.50
184885-89-0	-11.17±0.65	112570-87-3	-11.10±0.33	85798-19-2	-11.07±0.45
20829-55-4	-11.17±0.24	115178-50-2	-11.10±0.28	88191-06-4	-11.07±0.45
481-18-5	-11.17±0.48	116179-22-7	-11.10±0.32	94935-97-4	-11.07±0.06
63109-17-1	-11.17±0.34	118574-73-5	-11.10±0.18	95043-18-8	-11.07±0.15
648883-30-1	-11.17±0.18	121379-48-4	-11.10±0.55	112693-24-0	-11.06±0.15
78183-29-6	-11.17±0.26	125640-31-5	-11.10±0.41	114571-91-4	-11.06±0.34
85249-29-2	-11.17±0.48	157459-24-0	-11.10±0.38	116972-93-1	-11.06±0.27
85798-13-6	-11.17±0.29	184885-92-5	-11.10±0.35	123158-95-2	-11.06±0.25
866403-71-6	-11.17±0.35	218916-92-8	-11.10±0.50	137529-91-0	-11.06±0.53
86748-29-0	-11.17±0.30	264618-21-5	-11.10±0.31	154071-70-2	-11.06±0.42
870095-33-3	-11.17±0.50	325690-71-9	-11.10±0.73	166038-28-4	-11.06±0.36
96363-06-3	-11.17±0.23	52043-01-3	-11.10±0.21	173681-52-2	-11.06±0.44
114820-29-0	-11.16±0.32	706784-74-9	-11.10±0.52	173693-48-6	-11.06±0.52
153585-64-9	-11.16±0.37	757976-67-3	-11.10±0.36	516-85-8	-11.05±0.34
351198-08-8	-11.15±0.46	77680-78-5	-11.10±0.45	62335-06-2	-11.05±0.31
57539-70-5	-11.15±0.55	866025-62-9	-11.10±0.14	6673-68-3	-11.05±0.38
65527-04-0	-11.15±0.21	92355-85-6	-11.10±0.52	79217-17-7	-11.05±1.00
65527-05-1	-11.15±0.21	96157-96-9	-11.10±0.26	80442-78-0	-11.05±0.36
75246-76-3	-11.15±0.26	103190-14-3	-11.09±0.21	80442-79-1	-11.05±0.36
782491-79-6	-11.15±0.22	122970-14-3	-11.09±0.49	81296-42-6	-11.05±0.56
84323-28-4	-11.15±0.12	139220-18-1	-11.09±0.35	82915-90-0	-11.05±0.52
85733-79-5	-11.15±0.26	147395-97-9	-11.09±0.31	126060-09-1	-11.04±0.30
858950-38-6	-11.15±0.24	157207-88-0	-11.09±0.25	131467-00-0	-11.04±0.34
147641-74-5	-11.14±0.37	164991-65-5	-11.09±0.37	131565-70-3	-11.04±0.25
151345-08-3	-11.14±0.31	171784-10-4	-11.09±0.19	14270-73-6	-11.04±0.75
151890-81-2	-11.14±0.27	199600-39-0	-11.09±0.62	145163-97-9	-11.04±0.22
2034-72-2	-11.14±0.32	244303-77-3	-11.09±0.54	151890-80-1	-11.04±0.42
129239-13-0	-11.13±1.14	105239-70-1	-11.08±0.27	199165-88-3	-11.04±0.34
157078-48-3	-11.13±1.90	131487-01-9	-11.08±0.36	89955-50-0	-11.04±0.41
174630-08-1	-11.13±0.75	145038-56-8	-11.08±0.23	128129-56-6	-11.03±0.44
17757-07-2	-11.13±0.38	145940-74-5	-11.08±0.44	132410-40-3	-11.03±0.40
200942-18-3	-11.13±0.39	148472-99-5	-11.08±0.64	137415-08-8	-11.03±0.41
26047-31-4	-11.13±0.32	184584-40-5	-11.08±0.41	145680-52-0	-11.03±0.35
2643-02-9	-11.13±0.34	306971-06-2	-11.08±0.19	158758-41-9	-11.03±0.36
315209-08-6	-11.13±0.31	33886-74-7	-11.08±0.12	28949-66-8	-11.03±0.37
508193-31-5	-11.13±0.38	400710-54-5	-11.08±0.40	385815-30-5	-11.03±0.33
50909-86-9	-11.13±0.44	489446-57-3	-11.08±0.79	60492-18-4	-11.03±0.36

70214-99-2	-11.13±0.76	52043-00-2	-11.08±0.38	71103-05-4	-11.03±0.44
75179-58-7	-11.13±0.34	6891-35-6	-11.08±0.23	851610-86-1	-11.03±0.50
79664-61-2	-11.13±0.23	74185-09-4	-11.08±0.18	85735-14-4	-11.03±0.19
81855-46-1	-11.13±0.77	84897-09-6	-11.08±0.40	866403-75-0	-11.03±0.38
82660-62-6	-11.13±0.33	86047-14-5	-11.08±0.22	874221-89-3	-11.03±0.39
851606-66-1	-11.13±0.20	106463-75-6	-11.07±0.19	94806-02-7	-11.03±0.06
119147-12-5	-11.12±0.29	116331-46-5	-11.07±0.49	94806-03-8	-11.03±0.40
211358-65-5	-11.12±0.43	14325-03-2	-11.07±0.28	94806-05-0	-11.03±0.23
306971-12-0	-11.12±0.32	153723-35-4	-11.07±0.71	96253-60-0	-11.03±0.32
38-26-6	-11.12±0.62	225662-09-9	-11.07±0.35	102679-93-6	-11.02±0.45
474779-75-4	-11.12±0.51	308098-61-5	-11.07±0.34	117857-72-4	-11.02±0.47
74185-13-0	-11.12±0.45	356566-84-2	-11.07±0.27	124392-06-9	-11.02±0.65
85733-78-4	-11.12±0.30	47922-48-5	-11.07±0.22	132410-38-9	-11.02±0.13
85798-12-5	-11.12±0.24	61897-88-9	-11.07±0.45	144436-06-6	-11.02±0.49
12771-72-1	-11.11±0.39	74185-10-7	-11.07±0.62	151890-89-0	-11.02±0.57
219774-74-0	-11.11±0.52	81575-78-2	-11.07±0.27	174024-98-7	-11.02±0.23
256377-68-1	-11.11±0.52	847613-59-6	-11.07±0.42	306967-75-9	-11.02±0.63
465-95-2	-11.02±0.39	16250-61-6	-11.01±0.37	32450-26-3	-11.00±0.19
474417-54-4	-11.02±0.28	169626-35-1	-11.01±0.39	326794-17-6	-11.00±0.39
494862-71-4	-11.02±0.41	201213-05-0	-11.01±0.27	37926-43-5	-11.00±0.32
564-16-9	-11.02±0.16	201800-59-1	-11.01±0.34	4030-92-6	-11.00±0.30
631913-68-3	-11.02±0.25	20780-41-0	-11.01±0.36	4657-58-3	-11.00±0.14
64825-79-2	-11.02±0.52	244157-96-8	-11.01±0.58	474-70-4	-11.00±0.47
69081-87-4	-11.02±0.19	100667-75-2	-11.00±0.29	58497-29-3	-11.00±0.28
69819-82-5	-11.02±0.25	105645-77-0	-11.00±0.34	58514-32-2	-11.00±0.47
82471-15-6	-11.02±0.79	120416-68-4	-11.00±0.44	62014-96-4	-11.00±0.42
86783-84-8	-11.02±0.25	122540-27-6	-11.00±0.26	745828-23-3	-11.00±0.34
125282-11-3	-11.01±0.40	134887-29-9	-11.00±0.66	81306-61-8	-11.00±0.32
13963-13-8	-11.01±0.25	145038-57-9	-11.00±0.24	851610-88-3	-11.00±0.24
145163-99-1	-11.01±0.48	148149-82-0	-11.00±0.44	86748-30-3	-11.00±0.19
14660-91-4	-11.01±0.40	211486-15-6	-11.00±0.40	95189-17-6	-11.00±0.26
156953-95-6	-11.01±0.73	306971-10-8	-11.00±0.22		

Supplementary Table S2: Molecular docking analysis for potential inhibitor compounds (candidate molecules) at the **rapamycin binding site** of **mTor** kinase selected among **Super Natural II database** [2].

Super Natural II	ΔG, mean±S.D.	Super Natural II	ΔG, mean±S.D.	Super Natural II	ΔG, mean±S.D.
SN00128384	-15.98±1.07	SN00169417	-14.46±0.44	SN00167982	-14.02±0.8
SN00169366	-15.97±0.74	SN00136698	-14.44±1.24	SN00286831	-14.02±0.71
SN00130058	-15.57±0.89	SN00167934	-14.43±0.28	SN00371872	-14.02±0.55
SN00169369	-15.57±0.53	SN00393875	-14.43±0.5	SN00111395	-14.01±0.4
SN00367917	-15.42±0.36	SN00081740	-14.42±0.4	SN00323840	-14.01±0.73
SN00169470	-15.38±0.2	SN00169362	-14.4±0.51	SN00136695	-14±0.8
SN00387839	-15.34±0.81	SN00169411	-14.4±0.39	SN00169439	-13.99±0.84
SN00130898	-15.17±0.37	SN00169416	-14.4±0.38	SN00321411	-13.98±0.28
SN00168000	-15.13±0.52	SN00113632	-14.38±0.47	SN00113542	-13.97±0.52
SN00335277	-15.12±0.36	SN00170378	-14.38±0.72	SN00169459	-13.97±0.46
SN00164826	-15.1±0.43	SN00395571	-14.38±0.3	SN00169460	-13.97±0.35
SN00383686	-15.07±0.92	SN00130963	-14.34±0.92	SN00275124	-13.97±0.53

SN00074793	-15.02±0.94	SN00274542	-14.34±0.65	SN00239003	-13.96±0.66
SN00115843	-14.99±0.64	SN00248218	-14.33±0.37	SN00386116	-13.96±0.4
SN00169375	-14.99±0.49	SN00343570	-14.33±0.35	SN00254348	-13.94±0.52
SN00376086	-14.97±0.24	SN00354912	-14.33±0.46	SN00260096	-13.94±0.61
SN00304654	-14.96±1	SN00167953	-14.32±0.37	SN00288290	-13.94±0.4
SN00330635	-14.96±0.96	SN00169428	-14.3±0.71	SN00059271	-13.93±0.55
SN00169424	-14.92±0.45	SN00348164	-14.27±0.45	SN00080936	-13.93±0.42
SN00290152	-14.9±1.07	SN00111460	-14.24±0.76	SN00169443	-13.93±0.47
SN00169633	-14.88±1.07	SN00347694	-14.24±0.27	SN00169469	-13.93±1.21
SN00232174	-14.88±0.31	SN00089681	-14.23±0.32	SN00243072	-13.93±0.45
SN00082747	-14.87±0.71	SN00169452	-14.23±0.68	SN00269626	-13.93±0.59
SN00330261	-14.86±0.99	SN00238877	-14.23±0.49	SN00344654	-13.93±0.55
SN00106774	-14.8±0.32	SN00307850	-14.22±0.61	SN00130239	-13.92±0.8
SN00169373	-14.79±0.84	SN00129404	-14.21±0.45	SN00169368	-13.92±0.61
SN00169438	-14.79±0.46	SN00169361	-14.21±0.71	SN00172800	-13.92±0.6
SN00172785	-14.79±0.83	SN00277812	-14.2±0.32	SN00081043	-13.91±0.72
SN00167918	-14.74±0.86	SN00167950	-14.19±0.59	SN00113230	-13.91±0.54
SN00264474	-14.73±0.94	SN00167970	-14.19±0.76	SN00129111	-13.91±0.42
SN00169425	-14.7±0.7	SN00111397	-14.18±0.49	SN00111564	-13.9±0.48
SN00037382	-14.69±0.8	SN00268217	-14.18±0.52	SN00130855	-13.9±0.36
SN00125316	-14.69±0.37	SN00288697	-14.18±1.1	SN00169941	-13.9±0.34
SN00119553	-14.67±0.25	SN00097689	-14.17±0.48	SN00341689	-13.9±1.05
SN00167887	-14.64±0.84	SN00169387	-14.17±0.74	SN00167811	-13.89±0.16
SN00394378	-14.64±0.33	SN00263404	-14.17±0.79	SN00169405	-13.89±0.52
SN00169448	-14.63±0.38	SN00111571	-14.16±0.58	SN00253506	-13.89±0.54
SN00137074	-14.62±1.4	SN00169406	-14.14±0.57	SN00322991	-13.89±0.5
SN00376674	-14.61±0.54	SN00323189	-14.14±0.75	SN00169384	-13.88±0.47
SN00169451	-14.6±0.79	SN00137008	-14.13±0.61	SN00267944	-13.88±0.54
SN00066079	-14.59±0.79	SN00169461	-14.13±0.52	SN00337878	-13.88±0.63
SN00238129	-14.59±0.51	SN00111415	-14.12±0.28	SN00082502	-13.87±0.99
SN00169370	-14.58±0.44	SN00169635	-14.09±1.14	SN00322705	-13.87±0.74
SN00282638	-14.57±0.3	SN00297098	-14.09±0.63	SN00097229	-13.86±0.25
SN00172758	-14.52±0.76	SN00307022	-14.09±0.35	SN00167973	-13.86±0.64
SN00239538	-14.52±0.41	SN00111437	-14.08±0.37	SN00169367	-13.86±0.84
SN00237128	-14.51±0.4	SN00379780	-14.07±0.79	SN00172770	-13.86±0.5
SN00123829	-14.49±0.61	SN00167990	-14.06±0.95	SN00173925	-13.86±0.38
SN00228019	-14.49±0.69	SN00253708	-14.06±0.6	SN00264249	-13.86±1.67
SN00169371	-14.48±0.22	SN00167965	-14.04±0.68	SN00350405	-13.86±0.49
SN00169617	-14.48±0.82	SN00340494	-14.04±0.14	SN00397406	-13.86±0.21
SN00301026	-14.48±0.18	SN00169383	-14.03±0.66	SN00089693	-13.84±0.24
SN00081734	-14.47±0.29	SN00174849	-14.03±0.61	SN00169363	-13.84±0.97
SN00169427	-14.47±0.32	SN00378076	-14.03±0.69	SN00169429	-13.84±0.26
SN00297928	-13.84±0.57	SN00237456	-13.64±0.56	SN00101954	-13.51±0.33
SN00169942	-13.83±0.29	SN00384384	-13.64±0.37	SN00170384	-13.51±0.35
SN00167979	-13.82±0.49	SN00100399	-13.63±0.87	SN00240611	-13.51±0.36
SN00214272	-13.82±0.56	SN00125315	-13.63±0.23	SN00241069	-13.51±0.89
SN00391214	-13.82±0.39	SN00136711	-13.63±0.47	SN00242230	-13.51±0.48
SN00397355	-13.82±0.85	SN00169381	-13.63±1.12	SN00247481	-13.51±0.18
SN00128376	-13.81±0.36	SN00236890	-13.63±0.66	SN00260522	-13.51±0.66
SN00318192	-13.81±0.54	SN00137143	-13.62±0.74	SN00352768	-13.51±0.33
SN00128796	-13.8±0.54	SN00141055	-13.62±0.38	SN00369562	-13.51±0.51

SN00214050	-13.8±0.46	SN00169437	-13.62±0.37	SN00380580	-13.51±0.38
SN00359062	-13.8±0.8	SN00170401	-13.62±0.38	SN00106727	-13.5±0.85
SN00114308	-13.79±0.28	SN00174908	-13.62±0.59	SN00114309	-13.5±0.25
SN00135616	-13.79±0.36	SN00081767	-13.61±0.53	SN00142234	-13.5±0.53
SN00227072	-13.79±0.5	SN00164496	-13.61±0.66	SN00300506	-13.5±0.47
SN00270743	-13.79±0.35	SN00169440	-13.61±0.55	SN00323388	-13.5±0.37
SN00081067	-13.78±0.67	SN00268935	-13.61±1.2	SN00326320	-13.5±1.02
SN00294655	-13.78±0.59	SN00383086	-13.61±0.28	SN00380662	-13.5±0.6
SN00078114	-13.77±0.48	SN00097956	-13.6±0.43	SN00389675	-13.5±0.29
SN00116397	-13.77±0.6	SN00169430	-13.6±0.73	SN00139953	-13.49±0.79
SN00081079	-13.76±0.74	SN00169938	-13.6±0.39	SN00169374	-13.49±0.26
SN00169357	-13.76±0.76	SN00170390	-13.6±0.58	SN00176546	-13.49±0.65
SN00239757	-13.76±0.34	SN00172791	-13.6±0.49	SN00226829	-13.49±0.69
SN00097206	-13.74±0.36	SN00296952	-13.6±0.33	SN00354803	-13.49±0.33
SN00169457	-13.74±0.61	SN00310110	-13.6±0.52	SN00111420	-13.48±0.32
SN00316971	-13.74±0.72	SN00081634	-13.59±0.35	SN00113250	-13.48±0.34
SN00344013	-13.74±0.63	SN00169419	-13.59±0.45	SN00169939	-13.48±0.59
SN00091350	-13.73±0.51	SN00233069	-13.59±0.26	SN00172771	-13.48±0.36
SN00078117	-13.72±0.61	SN00097418	-13.58±0.32	SN00172824	-13.48±0.44
SN00272690	-13.72±0.19	SN00226941	-13.58±0.4	SN00175908	-13.48±0.91
SN00327455	-13.72±1.26	SN00349017	-13.58±0.34	SN00384061	-13.48±0.49
SN00113232	-13.71±0.83	SN00057684	-13.57±0.55	SN00133377	-13.47±0.56
SN00246695	-13.71±0.67	SN00176544	-13.57±0.45	SN00164139	-13.47±0.64
SN00364759	-13.71±0.44	SN00296040	-13.57±0.57	SN00170398	-13.47±0.44
SN00379189	-13.71±0.38	SN00058537	-13.56±0.48	SN00170692	-13.47±0.46
SN00098044	-13.7±0.4	SN00084674	-13.56±0.31	SN00233772	-13.47±0.36
SN00118874	-13.7±0.46	SN00139965	-13.56±0.37	SN00111703	-13.46±0.4
SN00168374	-13.7±0.39	SN00171845	-13.56±0.27	SN00167708	-13.46±0.62
SN00169376	-13.7±0.6	SN00173958	-13.56±0.53	SN00236102	-13.46±0.35
SN00256709	-13.7±0.69	SN00226062	-13.56±0.66	SN00297135	-13.46±0.33
SN00173918	-13.69±0.44	SN00392830	-13.56±0.7	SN00128374	-13.44±0.54
SN00296190	-13.69±0.46	SN00427143	-13.56±0.35	SN00167932	-13.44±0.59
SN00368224	-13.69±0.37	SN00097225	-13.54±0.43	SN00229183	-13.44±0.37
SN00078115	-13.68±0.63	SN00119196	-13.54±0.33	SN00249152	-13.44±0.34
SN00281885	-13.68±0.64	SN00246166	-13.54±1.12	SN00261527	-13.44±0.93
SN00337460	-13.68±0.32	SN00250332	-13.54±0.26	SN00293304	-13.44±0.83
SN00170394	-13.67±0.44	SN00352431	-13.54±0.27	SN00323402	-13.44±0.68
SN00281856	-13.67±1.06	SN00123863	-13.53±0.5	SN00364809	-13.44±0.33
SN00288395	-13.67±0.29	SN00278770	-13.53±0.42	SN00097411	-13.43±0.32
SN00261089	-13.66±0.66	SN00365186	-13.53±0.32	SN00106730	-13.43±0.97
SN00401354	-13.66±0.55	SN00172789	-13.52±0.47	SN00121863	-13.43±0.36
SN00167810	-13.64±0.39	SN00173913	-13.52±0.42	SN00159020	-13.43±0.62
SN00169372	-13.64±0.66	SN00259728	-13.52±0.43	SN00169385	-13.43±0.81
SN00169426	-13.64±0.46	SN00284649	-13.52±0.19	SN00169412	-13.43±0.75
SN00225970	-13.64±0.64	SN00304457	-13.52±0.53	SN00173175	-13.43±0.26
SN00319849	-13.43±0.52	SN00168388	-13.36±0.51	SN00239708	-13.3±0.46
SN00113059	-13.42±0.88	SN00265184	-13.36±0.55	SN00245140	-13.3±0.22
SN00113541	-13.42±0.49	SN00290515	-13.36±0.37	SN00151101	-13.29±0.52
SN00380133	-13.42±0.26	SN00136713	-13.34±0.49	SN00168356	-13.29±0.45
SN00113761	-13.41±0.48	SN00139973	-13.34±0.27	SN00169409	-13.29±0.3
SN00120909	-13.41±0.33	SN00141013	-13.34±0.74	SN00169953	-13.29±0.36

SN00167456	-13.41±0.33	SN00170395	-13.34±0.5	SN00172578	-13.29±0.39
SN00167948	-13.41±0.54	SN00172352	-13.34±0.45	SN00173029	-13.29±0.29
SN00169396	-13.41±1.1	SN00173144	-13.34±1.32	SN00257182	-13.29±0.71
SN00169410	-13.41±0.92	SN00174834	-13.34±0.69	SN00298985	-13.29±0.51
SN00172830	-13.41±0.44	SN00270457	-13.34±0.4	SN00371902	-13.29±0.68
SN00173921	-13.41±0.61	SN00295429	-13.34±0.51	SN00399954	-13.29±0.34
SN00245953	-13.41±0.47	SN00105976	-13.33±0.69	SN00078665	-13.28±0.36
SN00261587	-13.41±1.22	SN00106779	-13.33±0.4	SN00098043	-13.28±0.91
SN00295246	-13.41±0.48	SN00169433	-13.33±0.67	SN00108027	-13.28±0.16
SN00337526	-13.41±0.78	SN00313656	-13.33±0.34	SN00126281	-13.28±0.66
SN00341502	-13.41±0.24	SN00337147	-13.33±0.43	SN00139966	-13.28±0.57
SN00084682	-13.4±0.63	SN00381153	-13.33±0.42	SN00168359	-13.28±0.64
SN00097687	-13.4±0.34	SN00381225	-13.33±0.27	SN00169364	-13.28±0.9
SN00113539	-13.4±0.51	SN00097227	-13.32±0.69	SN00171132	-13.28±0.54
SN00139951	-13.4±0.73	SN00107651	-13.32±0.37	SN00173907	-13.28±0.5
SN00167956	-13.4±0.89	SN00113631	-13.32±0.78	SN00249525	-13.28±0.42
SN00172575	-13.4±0.41	SN00132224	-13.32±0.26	SN00262135	-13.28±0.4
SN00172786	-13.4±0.55	SN00134172	-13.32±0.68	SN00080974	-13.27±0.42
SN00173928	-13.4±0.74	SN00162890	-13.32±0.22	SN00151510	-13.27±1.07
SN00173971	-13.4±0.49	SN00167812	-13.32±0.3	SN00172798	-13.27±0.59
SN00176973	-13.4±0.37	SN00171706	-13.32±1.05	SN00172807	-13.27±0.49
SN00258951	-13.4±0.42	SN00172273	-13.32±0.59	SN00173960	-13.27±0.65
SN00082617	-13.39±1.14	SN00174876	-13.32±0.41	SN00174895	-13.27±0.45
SN00126280	-13.39±0.56	SN00224043	-13.32±0.32	SN00176984	-13.27±0.53
SN00169386	-13.39±0.77	SN00224821	-13.32±0.35	SN00270246	-13.27±0.63
SN00170255	-13.39±0.72	SN00236253	-13.32±0.36	SN00277065	-13.27±0.92
SN00172793	-13.39±1.11	SN00290973	-13.32±0.65	SN00393370	-13.27±0.29
SN00172855	-13.39±0.5	SN00295732	-13.32±0.4	SN00004671	-13.26±0.31
SN00243155	-13.39±0.47	SN00129447	-13.31±0.49	SN00058256	-13.26±0.61
SN00261583	-13.39±0.45	SN00130378	-13.31±0.44	SN00078968	-13.26±0.24
SN00051604	-13.38±0.51	SN00135095	-13.31±0.93	SN00128800	-13.26±0.29
SN00059871	-13.38±0.44	SN00167809	-13.31±0.38	SN00167706	-13.26±0.98
SN00111609	-13.38±0.34	SN00169358	-13.31±0.39	SN00169651	-13.26±0.55
SN00111702	-13.38±0.41	SN00170382	-13.31±0.63	SN00172689	-13.26±0.31
SN00169360	-13.38±0.83	SN00173892	-13.31±0.72	SN00174921	-13.26±0.47
SN00169467	-13.38±0.68	SN00261952	-13.31±0.43	SN00257818	-13.26±0.42
SN00169652	-13.38±0.9	SN00371690	-13.31±0.93	SN00259282	-13.26±1.25
SN00170403	-13.38±0.41	SN00383457	-13.31±0.45	SN00358372	-13.26±0.48
SN00170408	-13.38±0.3	SN00074337	-13.3±0.87	SN00385666	-13.26±0.68
SN00012590	-13.37±0.33	SN00108001	-13.3±0.59	SN00051582	-13.24±0.42
SN00143594	-13.37±0.59	SN00129403	-13.3±0.49	SN00056789	-13.24±0.71
SN00167111	-13.37±0.69	SN00136704	-13.3±0.42	SN00111674	-13.24±0.39
SN00263628	-13.37±0.36	SN00146412	-13.3±0.51	SN00111676	-13.24±0.33
SN00277690	-13.37±0.49	SN00164493	-13.3±0.54	SN00170396	-13.24±0.52
SN00336143	-13.37±0.59	SN00168380	-13.3±0.4	SN00174886	-13.24±0.27
SN00355930	-13.37±0.22	SN00169394	-13.3±0.64	SN00276332	-13.24±0.3
SN00381519	-13.37±0.7	SN00225740	-13.3±0.33	SN00366405	-13.24±0.38
SN00142239	-13.36±0.47	SN00228121	-13.3±0.88	SN00080896	-13.23±0.17
SN00084702	-13.23±0.31	SN00008387	-13.19±0.36	SN00176547	-13.14±0.7
SN00106729	-13.23±0.64	SN00059442	-13.19±0.53	SN00237320	-13.14±0.38
SN00121871	-13.23±0.19	SN00111576	-13.19±0.2	SN00318097	-13.14±0.58

SN00128073	-13.23±0.24	SN00111663	-13.19±0.25	SN00322232	-13.14±0.49
SN00132837	-13.23±0.41	SN00114250	-13.19±0.49	SN00346826	-13.14±0.34
SN00139770	-13.23±0.49	SN00168445	-13.19±0.46	SN00362478	-13.14±0.84
SN00170400	-13.23±0.77	SN00173909	-13.19±0.25	SN00391697	-13.14±0.57
SN00173927	-13.23±0.41	SN00236462	-13.19±0.37	SN00111675	-13.13±0.4
SN00177126	-13.23±0.57	SN00396821	-13.19±0.64	SN00114045	-13.13±0.34
SN00239403	-13.23±0.26	SN00015562	-13.18±0.48	SN00114929	-13.13±0.6
SN00260416	-13.23±0.19	SN00082651	-13.18±0.36	SN00121449	-13.13±0.34
SN00312143	-13.23±0.33	SN00109705	-13.18±0.72	SN00128075	-13.13±0.13
SN00390239	-13.23±0.35	SN00111711	-13.18±0.37	SN00132230	-13.13±0.56
SN00081539	-13.22±0.47	SN00128385	-13.18±0.74	SN00141410	-13.13±0.42
SN00106712	-13.22±0.88	SN00169407	-13.18±0.48	SN00169379	-13.13±0.57
SN00106728	-13.22±0.72	SN00173926	-13.18±0.58	SN00169401	-13.13±0.78
SN00107343	-13.22±0.47	SN00282816	-13.18±0.58	SN00172576	-13.13±0.89
SN00113504	-13.22±0.75	SN00360740	-13.18±0.26	SN00173575	-13.13±0.62
SN00126645	-13.22±0.69	SN00362382	-13.18±0.36	SN00252317	-13.13±0.3
SN00168033	-13.22±0.58	SN00366650	-13.18±0.28	SN00275296	-13.13±0.52
SN00168449	-13.22±0.5	SN00379911	-13.18±0.48	SN00051581	-13.12±0.41
SN00171123	-13.22±0.49	SN00059388	-13.17±0.65	SN00080933	-13.12±0.32
SN00171712	-13.22±0.53	SN00080939	-13.17±0.28	SN00099712	-13.12±0.67
SN00172586	-13.22±0.89	SN00108028	-13.17±0.33	SN00099739	-13.12±0.57
SN00172805	-13.22±0.67	SN00111586	-13.17±0.56	SN00113247	-13.12±0.36
SN00239126	-13.22±0.85	SN00113249	-13.17±0.66	SN00113629	-13.12±0.52
SN00245060	-13.22±0.76	SN00126282	-13.17±0.74	SN00114348	-13.12±0.37
SN00327269	-13.22±0.38	SN00167479	-13.17±0.6	SN00123349	-13.12±0.29
SN00111652	-13.21±0.44	SN00172134	-13.17±0.98	SN00128855	-13.12±0.52
SN00113218	-13.21±0.44	SN00173917	-13.17±0.55	SN00131673	-13.12±0.56
SN00114971	-13.21±0.54	SN00296536	-13.17±0.44	SN00141017	-13.12±0.37
SN00141018	-13.21±0.36	SN00300438	-13.17±0.53	SN00161478	-13.12±0.25
SN00157973	-13.21±0.97	SN00351762	-13.17±0.37	SN00258210	-13.12±0.38
SN00172772	-13.21±0.23	SN00005498	-13.16±0.63	SN00266522	-13.12±0.81
SN00253892	-13.21±0.83	SN00082503	-13.16±0.29	SN00273506	-13.12±0.5
SN00266324	-13.21±0.34	SN00122833	-13.16±0.54	SN00331477	-13.12±0.36
SN00298123	-13.21±0.74	SN00126328	-13.16±0.42	SN00347599	-13.12±0.37
SN00346715	-13.21±0.57	SN00133950	-13.16±0.93	SN00352443	-13.12±0.49
SN00062444	-13.2±0.4	SN00139969	-13.16±0.48	SN00012599	-13.11±0.78
SN00080932	-13.2±0.48	SN00172291	-13.16±0.35	SN00020195	-13.11±0.24
SN00081619	-13.2±0.47	SN00331859	-13.16±0.41	SN00037381	-13.11±0.28
SN00083037	-13.2±0.44	SN00368459	-13.16±1.37	SN00098042	-13.11±0.37
SN00097226	-13.2±0.6	SN00391702	-13.16±0.66	SN00108118	-13.11±0.7
SN00097526	-13.2±0.36	SN00427137	-13.16±0.46	SN00108496	-13.11±0.44
SN00111701	-13.2±0.55	SN00052444	-13.14±0.32	SN00168436	-13.11±0.69
SN00129322	-13.2±0.55	SN00106930	-13.14±0.43	SN00178201	-13.11±0.41
SN00142238	-13.2±0.5	SN00121862	-13.14±0.36	SN00298711	-13.11±0.3
SN00167993	-13.2±1.14	SN00128072	-13.14±0.33	SN00310178	-13.11±0.41
SN00169365	-13.2±0.64	SN00136712	-13.14±0.25	SN00356104	-13.11±0.92
SN00169441	-13.2±0.38	SN00170253	-13.14±0.4	SN00357461	-13.11±0.58
SN00171707	-13.2±0.32	SN00172787	-13.14±0.52	SN00358384	-13.11±0.33
SN00176530	-13.2±0.86	SN00172818	-13.14±0.37	SN00397632	-13.11±0.66
SN00299625	-13.2±0.39	SN00173914	-13.14±0.67	SN00083218	-13.1±0.42
SN00345686	-13.2±0.51	SN00173916	-13.14±0.78	SN00084750	-13.1±0.82

SN00106967	-13.1±0.46	SN00141487	-13.07±0.29	SN00111617	-13.03±0.47
SN00107978	-13.1±0.34	SN00168463	-13.07±0.51	SN00111712	-13.03±0.42
SN00109757	-13.1±0.94	SN00172784	-13.07±0.45	SN00157950	-13.03±1.02
SN00114567	-13.1±0.58	SN00231454	-13.07±0.45	SN00162428	-13.03±0.97
SN00115698	-13.1±0.44	SN00238169	-13.07±0.4	SN00169445	-13.03±0.46
SN00118873	-13.1±0.45	SN00246480	-13.07±0.28	SN00169450	-13.03±0.51
SN00118966	-13.1±0.46	SN00302481	-13.07±0.69	SN00169465	-13.03±0.4
SN00128422	-13.1±0.35	SN00305691	-13.07±0.86	SN00172587	-13.03±0.72
SN00129448	-13.1±0.56	SN00352327	-13.07±0.34	SN00246352	-13.03±0.36
SN00167927	-13.1±0.5	SN00051583	-13.06±0.63	SN00264331	-13.03±0.52
SN00167983	-13.1±1.06	SN00077583	-13.06±0.8	SN00341572	-13.03±0.42
SN00169456	-13.1±0.32	SN00081635	-13.06±0.5	SN00350731	-13.03±0.28
SN00169466	-13.1±0.56	SN00111618	-13.06±0.42	SN00350939	-13.03±0.31
SN00171097	-13.1±0.5	SN00111625	-13.06±0.43	SN00380271	-13.03±0.51
SN00172799	-13.1±0.49	SN00113229	-13.06±0.38	SN00427145	-13.03±0.78
SN00173922	-13.1±0.67	SN00115081	-13.06±0.43	SN00091501	-13.02±0.36
SN00174844	-13.1±0.43	SN00129492	-13.06±0.6	SN00107402	-13.02±0.41
SN00174852	-13.1±0.32	SN00133970	-13.06±0.32	SN00111673	-13.02±0.63
SN00311638	-13.1±0.97	SN00136710	-13.06±0.3	SN00119083	-13.02±0.39
SN00334224	-13.1±0.44	SN00167494	-13.06±0.88	SN00123510	-13.02±0.37
SN00008385	-13.09±0.3	SN00171122	-13.06±0.34	SN00154784	-13.02±0.34
SN00012601	-13.09±0.83	SN00172354	-13.06±0.42	SN00155161	-13.02±0.59
SN00089691	-13.09±0.36	SN00172612	-13.06±0.5	SN00166919	-13.02±0.33
SN00097688	-13.09±0.53	SN00245319	-13.06±0.25	SN00167949	-13.02±0.87
SN00099738	-13.09±0.55	SN00247179	-13.06±0.62	SN00169454	-13.02±0.64
SN00131056	-13.09±0.63	SN00289906	-13.06±0.49	SN00170399	-13.02±0.54
SN00133706	-13.09±0.24	SN00297321	-13.06±0.43	SN00172802	-13.02±0.58
SN00158885	-13.09±0.7	SN00304569	-13.06±0.5	SN00173042	-13.02±0.48
SN00173298	-13.09±0.48	SN00325070	-13.06±0.59	SN00244833	-13.02±0.62
SN00281717	-13.09±0.53	SN00338156	-13.06±0.38	SN00265480	-13.02±0.22
SN00307932	-13.09±0.51	SN00343402	-13.06±0.45	SN00276132	-13.02±0.66
SN00314913	-13.09±0.49	SN00366096	-13.06±0.69	SN00281219	-13.02±1
SN00080956	-13.08±0.2	SN00084678	-13.04±0.6	SN00329349	-13.02±0.59
SN00111466	-13.08±0.52	SN00096066	-13.04±0.34	SN00350549	-13.02±0.6
SN00111704	-13.08±0.44	SN00101586	-13.04±0.42	SN00052010	-13.01±0.45
SN00128074	-13.08±0.24	SN00105422	-13.04±0.59	SN00084069	-13.01±0.47
SN00133957	-13.08±0.4	SN00136682	-13.04±0.63	SN00091326	-13.01±0.33
SN00139931	-13.08±0.25	SN00139955	-13.04±0.32	SN00101331	-13.01±0.51
SN00150533	-13.08±0.35	SN00139974	-13.04±0.25	SN00107344	-13.01±0.23
SN00167116	-13.08±0.34	SN00167952	-13.04±0.67	SN00113839	-13.01±0.36
SN00167486	-13.08±0.38	SN00170407	-13.04±0.53	SN00114306	-13.01±0.33
SN00167575	-13.08±0.35	SN00171701	-13.04±0.55	SN00119195	-13.01±0.28
SN00174857	-13.08±0.55	SN00172788	-13.04±0.3	SN00169944	-13.01±0.28
SN00234930	-13.08±0.39	SN00283611	-13.04±0.64	SN00170380	-13.01±0.58
SN00259653	-13.08±0.41	SN00297425	-13.04±0.6	SN00252020	-13.01±0.79
SN00338890	-13.08±0.45	SN00345704	-13.04±0.58	SN00275452	-13.01±0.37
SN00355392	-13.08±0.27	SN00348816	-13.04±0.38	SN00319706	-13.01±0.34
SN00362462	-13.08±0.59	SN00387973	-13.04±0.27	SN00381395	-13.01±0.29
SN00369721	-13.08±0.59	SN00007650	-13.03±0.36	SN00015950	-13±0.45
SN00051603	-13.07±0.52	SN00035917	-13.03±0.42	SN00059694	-13±0.47
SN00081562	-13.07±0.3	SN00038957	-13.03±0.68	SN00111665	-13±0.29

SN00108034	-13.07±0.56	SN00083606	-13.03±0.42	SN00113215	-13±0.54
SN00111540	-13.07±0.28	SN00091357	-13.03±0.42	SN00113217	-13±0.72
SN00132319	-13.07±0.51	SN00109120	-13.03±0.31	SN00121452	-13±0.23
SN00166673	-13±0.41	SN00173915	-13±0.75	SN00280962	-13±0.72
SN00169398	-13±0.36	SN00238003	-13±0.35	SN00292426	-13±0.75
SN00350231	-13±0.5	SN00352197	-13±0.31	SN00390671	-13±0.58
SN00427228	-13±0.61				

Supplementary Table S3: Molecular docking analysis for potential inhibitor compounds (candidate molecules) at the **rapamycin binding site** of **mTor** kinase selected among **ZINC database natural products** [3].

ZINC database	ΔG, mean±S.D.	ZINC database	ΔG, mean±S.D.	ZINC database	ΔG, mean±S.D.
ZINC20807902	-13.04±0.47	ZINC04085602	-11.89±0.52	ZINC30819228	-11.48±0.18
ZINC02141237	-12.88±0.27	ZINC06624334	-11.89±0.49	ZINC09682151	-11.48±0.4
ZINC20807905	-12.84±0.24	ZINC09481951	-11.89±0.51	ZINC16671995	-11.47±0.22
ZINC08335520	-12.68±0.31	ZINC09792243	-11.88±0.29	ZINC30819218	-11.47±0.42
ZINC16284487	-12.61±0.34	ZINC14458042	-11.87±0.28	ZINC15468981	-11.44±0.49
ZINC10012991	-12.59±0.45	ZINC05235687	-11.86±0.31	ZINC02095105	-11.44±0.4
ZINC13120611	-12.59±0.37	ZINC08367675	-11.86±0.42	ZINC02103315	-11.44±0.65
ZINC16349120	-12.54±0.3	ZINC15725220	-11.84±0.57	ZINC24504925	-11.44±0.34
ZINC30819219	-12.44±0.81	ZINC09941299	-11.84±0.49	ZINC03331795	-11.44±0.34
ZINC00754174	-12.43±0.35	ZINC15725460	-11.82±0.81	ZINC04262734	-11.43±0.59
ZINC02928797	-12.39±0.76	ZINC09408505	-11.82±0.4	ZINC30819216	-11.38±0.28
ZINC27534122	-12.38±0.31	ZINC12949356	-11.81±0.53	ZINC06624464	-11.38±0.53
ZINC08918463	-12.37±0.25	ZINC09992471	-11.81±0.31	ZINC15725194	-11.37±0.47
ZINC14458178	-12.36±0.27	ZINC13642830	-11.8±0.59	ZINC20970245	-11.36±0.31
ZINC20938915	-12.34±0.47	ZINC15733799	-11.8±0.51	ZINC09530812	-11.33±0.43
ZINC09580205	-12.34±0.41	ZINC09577184	-11.8±0.51	ZINC20563426	-11.31±0.48
ZINC02928802	-12.33±0.56	ZINC09992380	-11.8±0.53	ZINC20563632	-11.27±0.39
ZINC09531209	-12.32±0.51	ZINC12583319	-11.79±0.34	ZINC13595929	-11.26±0.35
ZINC08367670	-12.3±0.37	ZINC16284359	-11.79±0.37	ZINC05151985	-11.23±0.29
ZINC12882862	-12.29±0.46	ZINC06624637	-11.77±0.4	ZINC08764854	-11.23±0.86
ZINC19879437	-12.28±0.36	ZINC13623590	-11.76±0.59	ZINC09076927	-11.23±0.39
ZINC01405393	-12.26±0.18	ZINC16269305	-11.76±0.49	ZINC00980500	-11.23±0.44
ZINC09940957	-12.23±0.42	ZINC30819248	-11.76±0.44	ZINC12440825	-11.22±0.34
ZINC15725210	-12.21±0.63	ZINC06232955	-11.76±0.6	ZINC06623908	-11.22±0.71
ZINC09992418	-12.19±0.39	ZINC09408500	-11.76±0.42	ZINC04936227	-11.19±0.41
ZINC00626315	-12.18±0.23	ZINC16349822	-11.73±0.25	ZINC12892460	-11.17±0.49
ZINC09224560	-12.18±0.48	ZINC16362158	-11.73±0.47	ZINC19660588	-11.14±0.36
ZINC15725066	-12.17±0.67	ZINC08966188	-11.73±0.5	ZINC12188773	-11.11±0.39
ZINC06232954	-12.11±0.33	ZINC11865630	-11.72±0.22	ZINC09408007	-11.08±0.36
ZINC00754173	-12.1±0.46	ZINC30819226	-11.71±0.37	ZINC04074031	-11.07±0.27
ZINC15725068	-12.09±0.28	ZINC12182014	-11.7±0.43	ZINC09043059	-11.06±0.59
ZINC09792247	-12.07±0.42	ZINC12682031	-11.7±0.3	ZINC06624359	-11.03±0.64
ZINC01223299	-12.06±0.27	ZINC15725231	-11.69±0.62	ZINC12753676	-11.02±0.33
ZINC11867425	-12.04±0.29	ZINC02093144	-11.69±0.28	ZINC05044064	-11±0.41
ZINC02107387	-12.02±0.37	ZINC20691520	-11.68±0.25	ZINC02094748	-11.89±0.25
ZINC11867433	-12±0.16	ZINC02092906	-11.68±0.35	ZINC03358607	-11.89±0.21
ZINC20938919	-11.99±0.36	ZINC01223300	-11.67±0.4	ZINC02148960	-11.51±0.17

ZINC16363024	-11.98±0.35	ZINC02098205	-11.67±0.34	ZINC02093614	-11.5±0.46
ZINC17093033	-11.98±0.27	ZINC13226339	-11.66±0.26		
ZINC22530327	-11.97±0.51	ZINC04744105	-11.64±0.33		
ZINC30819257	-11.97±0.42	ZINC01223288	-11.63±0.46		
ZINC12896272	-11.94±0.58	ZINC08857160	-11.63±0.38		
ZINC04744033	-11.94±0.31	ZINC30819259	-11.62±0.26		
ZINC08765269	-11.93±0.4	ZINC06623842	-11.61±0.42		
ZINC09940952	-11.93±0.36	ZINC08440247	-11.61±0.2		
ZINC06624400	-11.92±0.45	ZINC08490711	-11.56±0.41		
ZINC15725282	-11.91±0.56	ZINC13642449	-11.54±0.3		
ZINC12899220	-11.9±0.34	ZINC14458006	-11.54±0.36		
ZINC20990084	-11.9±0.42	ZINC04085605	-11.53±0.36		
ZINC23333375	-11.9±0.5	ZINC08765217	-11.53±0.38		
ZINC05235703	-11.9±0.33	ZINC09059583	-11.53±0.71		
ZINC11936239	-11.89±0.44	ZINC13691831	-11.51±0.51		

Supplementary Table S4: Molecular docking analysis for potential inhibitor compounds (candidate molecules) at the **ATP binding site** of **mTor kinase** selected among **Marine Natural Products** [1].

Marine natural products	ΔG, mean±S.D.	Marine natural products	ΔG, mean±S.D.	Marine natural products	ΔG, mean±S.D.
101383-39-5	-12.20±0.30	133625-26-0	-11.58±0.85	156953-93-4	-11.66±0.56
112088-56-9	-11.18±1.19	133805-03-5	-11.38±0.27	159736-39-7	-12.18±0.90
114571-91-4	-11.50±0.43	135340-01-1	-12.06±0.88	162465-80-7	-12.50±0.73
115982-22-4	-11.80±0.70	135824-74-7	-11.02±0.44	117694-96-9	-11.06±0.39
120154-96-3	-12.16±0.87	140429-37-4	-12.44±1.01	191-24-2	-12.26±0.27
121071-11-2	-11.02±0.38	103614-76-2	-11.04±0.29	206535-31-1	-11.02±0.39
122780-90-9	-11.54±0.49	152845-74-4	-11.08±0.39	214899-21-5	-11.06±0.67
128229-64-1	-11.94±0.53	156953-87-6	-11.02±0.79		

Supplementary Table S5: Molecular docking analysis for potential inhibitor compounds (candidate molecules) at the **ATP binding site** of **mTor** kinase selected among **Super Natural II database** [2].

Super Natural II	ΔG, mean±S.D.	Super Natural II	ΔG, mean±S.D.	Super Natural II	ΔG, mean±S.D.
SN00003459	-11.62±0.54	SN00126328	-11.40±0.90	SN00167993	-11.34±0.65
SN00005498	-11.42±0.69	SN00128374	-11.60±0.81	SN00168000	-11.66±0.25
SN00008379	-11.02±0.90	SN00128376	-11.40±0.75	SN00168004	-11.28±0.46
SN00008447	-11.00±1.01	SN00128384	-12.92±0.92	SN00111460	-11.46±0.72
SN00008773	-11.02±0.68	SN00128385	-11.82±0.98	SN00111466	-11.14±0.68
SN00017897	-11.10±0.83	SN00128827	-11.00±1.20	SN00111540	-11.00±0.45
SN00051580	-11.04±1.03	SN00129403	-11.38±0.66	SN00111542	-11.52±1.00
SN00051581	-11.12±0.84	SN00129404	-11.00±0.54	SN00111571	-11.26±0.95
SN00051582	-11.18±0.66	SN00129405	-12.60±1.56	SN00115210	-11.00±0.26
SN00051602	-11.04±0.81	SN00129448	-11.04±0.68	SN00116397	-11.34±0.27
SN00051604	-11.18±0.69	SN00130058	-12.98±0.41	SN00120425	-12.02±0.41
SN00052444	-11.10±1.10	SN00130855	-12.00±0.52	SN00123829	-11.82±1.09
SN00052930	-11.26±1.18	SN00130898	-12.62±0.69	SN00124057	-11.28±0.79
SN00058602	-11.42±0.90	SN00130963	-12.28±1.27	SN00125316	-11.60±0.51
SN00059432	-11.36±0.98	SN00131034	-11.28±1.66	SN00167932	-12.04±0.48
SN00061688	-11.56±1.13	SN00131673	-11.20±0.85	SN00167942	-11.32±0.67

SN00062324	-11.02±0.67	SN00132839	-11.14±1.04	SN00167952	-11.88±0.40
SN00062444	-11.30±1.12	SN00132840	-11.48±1.04	SN00167960	-11.14±0.77
SN00066079	-12.06±0.61	SN00133377	-11.52±0.24	SN00167965	-12.80±0.25
SN00074337	-11.30±0.80	SN00136688	-11.20±0.39	SN00167970	-12.08±0.41
SN00074793	-11.98±0.93	SN00136695	-11.34±0.38	SN00167976	-11.36±0.83
SN00076726	-11.10±0.73	SN00136914	-11.16±0.40	SN00167979	-12.04±0.21
SN00080936	-11.36±0.73	SN00137005	-11.06±0.74	SN00167982	-11.84±1.41
SN00080939	-11.16±0.72	SN00137007	-11.10±0.35	SN00167989	-11.28±0.91
SN00081043	-11.42±0.68	SN00137008	-11.14±0.66	SN00167990	-12.04±0.33
SN00081067	-11.34±0.69	SN00137009	-11.08±0.11	SN00111420	-11.12±0.31
SN00081079	-11.42±0.66	SN00137074	-11.84±0.48	SN00111437	-11.28±0.79
SN00081767	-11.36±0.84	SN00137076	-11.22±0.13	SN00167912	-11.56±0.53
SN00084682	-11.76±0.55	SN00142234	-11.82±0.93	SN00167918	-11.88±1.56
SN00086012	-11.06±0.77	SN00142238	-11.46±1.18		
SN00097206	-11.46±0.60	SN00151101	-11.42±0.66		
SN00097225	-11.06±0.65	SN00156840	-11.06±0.53		
SN00097229	-11.52±0.59	SN00162099	-11.06±0.93		
SN00106712	-11.68±0.24	SN00164826	-11.94±0.63		
SN00106774	-12.18±0.46	SN00166271	-11.14±0.34		
SN00108027	-11.78±0.89	SN00167115	-11.48±0.58		
SN00108028	-11.82±0.73	SN00167116	-11.12±0.52		
SN00108060	-11.44±0.94	SN00167892	-11.52±0.48		
SN00111397	-11.24±0.90	SN00167901	-11.38±0.54		

Supplementary Table S6: Physicochemical and toxicological parameters calculated for the selected compounds against the ATP binding site of mTOR based on molecular docking analysis. Each cluster groups compounds with structures with up to 70% structural similarity. Compounds whose name is CAS format belong to the Marine Natural Products database [1], while those compounds whose name begins with SN belong to the Super Natural II database [2].

Compound	Cluster 70%[a]	[a]Total Surface Area, Å^2	[a]TPSA, Å^2	cLogS[a]	MW[a]	cLogP[a]	HBA[a]	HBD[a]	Ro5 violations[a]	Druglikeness[a]	DrugScore[a]	RAT (LD50, mol/kg)[b]	Caco-2 Permeability (LogPapp, cm/s)[b]	TPT (pIGC50, µg/L)[b]	FT (pLC50, mg/L)[b]
114571-91-4	1	383.9	107.5	-7.281	595.3	2.965	8	2	1	0.405	0.07853	2.631	0.375	0.700	1.023
135824-74-7	1	383.9	107.5	-7.281	595.3	2.965	8	2	1	0.405	0.07853	2.631	0.375	0.700	1.023
117694-96-9	2	265.3	71.5	-6.325	385.4	3.277	6	1	0	5.488	0.50169	2.801	1.532	0.362	1.373
133805-03-5	3	237.0	118.2	-5.447	357.3	2.762	7	5	0	1.524	0.55632	2.597	0.231	0.153	1.289
135340-01-1	4	295.1	79.8	-6.055	427.5	2.877	6	2	0	1.910	0.47490	2.447	1.255	0.457	2.280
140429-37-4	5	305.1	99.5	-6.743	441.5	3.159	7	4	0	5.165	0.44521	2.529	0.358	0.393	1.398
SN00230471	5	298.2	126.0	-6.379	469.5	3.376	9	4	0	3.943	0.43415	2.460	0.296	0.476	1.369
SN00005498	6	379.7	74.7	-5.727	501.6	5.017	6	1	2	5.821	0.37008	2.614	0.551	0.349	1.542
SN00111397	6	410.6	93.1	-3.646	550.7	4.249	7	0	1	3.575	0.50267	2.773	0.865	0.565	0.616
SN00111420	6	385.8	93.1	-2.998	522.6	3.470	7	0	1	4.179	0.60912	2.797	0.852	0.464	0.608
SN00111437	6	399.6	93.1	-3.268	536.6	3.924	7	0	1	3.105	0.54974	2.741	0.801	0.515	0.595
SN00111571	6	400.1	93.1	-3.522	536.6	3.809	7	0	1	3.894	0.55219	2.776	0.765	0.523	0.609
SN00008379	7	340.7	69.7	-5.724	497.4	4.111	6	1	0	-0.857	0.09979	2.372	0.842	0.568	1.499
SN00008447	7	353.8	81.8	-5.993	500.5	3.851	7	2	1	-5.039	0.07583	2.357	0.826	0.542	1.640
SN00008773	7	356.0	93.5	-6.183	504.5	3.846	7	1	1	-5.138	0.07311	2.468	0.910	0.615	1.510
SN00017897	8	370.5	143.2	-6.904	498.6	3.385	9	2	0	-2.767	0.20633	2.473	1.010	0.573	1.371
SN00058602	9	300.9	79.8	-8.336	417.4	5.889	6	2	1	1.766	0.26763	2.550	1.174	0.800	1.147
SN00066079	10	344.0	95.9	-8.876	494.5	4.970	7	1	0	2.751	0.10182	2.414	1.065	0.489	1.307
SN00074793	11	330.0	117.4	-6.428	470.4	3.216	8	1	0	2.407	0.20231	2.569	1.040	0.554	1.029
SN00076726	12	274.5	53.4	-8.491	390.4	5.649	5	0	1	1.289	0.22060	2.543	1.562	0.334	1.264
SN00080936	13	358.6	58.4	-7.463	486.6	5.899	6	0	1	6.806	0.12842	2.578	1.105	0.347	1.249
SN00080939	13	334.1	58.4	-6.775	458.5	5.211	6	0	1	6.920	0.26864	2.390	0.997	0.359	1.394
SN00081043	13	349.5	58.4	-7.511	493.0	5.817	6	0	1	6.906	0.21352	2.396	0.882	0.642	1.262

SN00081067	13	340.4	58.4	-7.089	476.5	5.312	6	0	1	5.580	0.24749	2.432	0.951	0.626	1.147
SN00081079	13	340.4	58.4	-7.089	476.5	5.312	6	0	1	5.580	0.24749	2.427	0.802	0.525	1.246
SN00081767	13	341.5	88.7	-6.064	476.5	4.405	8	0	0	6.606	0.32421	2.254	0.707	0.259	1.403
SN00097206	13	374.3	58.4	-6.77	498.6	5.895	6	0	1	8.099	0.22226	2.500	0.698	0.394	1.241
SN00097225	13	347.8	58.4	-6.253	472.5	5.134	6	0	1	7.810	0.28342	2.367	0.635	0.261	1.548
SN00097229	13	368.1	76.9	-6.964	516.6	5.246	8	0	2	7.543	0.23433	2.212	0.728	0.280	1.238
SN00111460	13	363.5	58.4	-7.553	526.5	6.060	6	0	2	-0.270	0.13727	2.545	0.878	0.603	1.249
SN00111540	13	412.6	95.4	-7	576.6	5.106	10	0	2	7.487	0.21189	2.322	1.013	0.368	1.041
SN00106774	14	390.6	105.0	-4.431	537.6	3.764	7	1	1	2.570	0.48702	2.602	1.259	0.541	0.857
SN00115210	15	396.3	102.9	-3.093	531.6	3.437	9	1	1	3.193	0.58891	2.439	0.796	0.415	1.075
SN00128374	16	306.7	97.2	-7.291	442.4	4.691	6	1	0	-0.534	0.05304	2.754	1.225	0.809	0.662
SN00131673	17	265.1	70.1	-8.247	374.4	3.937	4	0	0	-0.838	0.07709	2.363	1.453	0.523	1.022
SN00169461	18	416.2	72.7	-6.904	574.9	5.997	4	4	2	1.528	0.21055	2.736	0.857	0.371	1.760
SN00172488	19	440.1	98.0	-7.035	628.9	6.073	5	4	2	-0.001	0.07404	3.020	0.985	0.897	0.778
SN00229558	19	391.1	113.0	-5.487	546.6	3.637	8	1	1	-0.318	0.24032	2.675	0.675	0.430	0.935
SN00290468	19	381.1	103.8	-5.425	530.6	3.849	7	1	1	-0.551	0.29535	2.638	1.111	0.418	1.064
SN00295457	19	381.1	103.8	-5.425	530.6	3.849	7	1	1	-0.551	0.29535	2.638	1.111	0.418	1.064
SN00173955	20	457.1	180.3	-6.816	637.7	2.417	11	3	2	-6.958	0.07667	2.692	0.642	0.759	0.826
SN00318021	20	373.3	116.2	-4.898	510.6	1.914	8	2	1	3.904	0.54231	2.663	0.611	0.436	1.216
SN00239987	21	354.5	49.4	-5.852	460.6	5.739	4	0	1	-1.562	0.20369	2.537	1.116	0.905	0.581
SN00246729	21	354.5	49.4	-5.852	460.6	5.852	4	0	1	1.712	0.31481	2.657	1.063	0.714	0.623
SN00245953	22	411.7	77.5	-6.026	578.7	5.110	8	1	2	1.983	0.22382	2.643	1.180	0.699	1.276
SN00352393	22	383.7	97.6	-6.867	548.6	5.531	8	2	2	-1.773	0.12085	2.598	0.627	0.691	1.433
SN00384061	22	383.7	97.6	-6.867	548.6	5.531	8	2	2	-1.773	0.12085	2.598	0.627	0.691	1.433
SN00352063	23	343.5	49.4	-5.692	446.6	5.582	4	0	1	0.443	0.30148	2.575	1.000	0.640	0.895
SN00364616	24	345.7	161.7	-6.66	527.5	3.197	10	4	1	2.464	0.22028	2.587	0.782	0.605	1.125
SN00383086	25	382.3	59.1	-1.622	616.8	2.008	8	0	1	-1.202	0.35389	2.896	0.838	0.532	0.885
SN00404273	26	333.9	32.9	-8.144	505.3	5.320	4	0	2	-1.033	0.05322	2.619	0.780	0.923	0.681

Abbreviations: Topological polar surface area (TPSA); molecular weight (MW); the calculated logarithm (base 10) of the solubility measured in mol/liter (cLogS); calculated logarithm of partition coefficient between n-octanol and water (cLogP); number of hydrogen bond donors (HBD); number of hydrogen bond acceptors

(HBA); violation of Lipinski's rules (Ro5 violations), FT, Fish Toxicity; TPT, *Tetrahymena Pyriformis* Toxicity; RAT, Rat Acute Toxicity. LD50 is the amount of a compound, given all at once, which causes the death of 50% (one half) of a group of test rats.

[a] These parameters were calculated using the DATAWARRIOR software v4.7.2 [4].

[b] These parameters were calculated using the http://lmmd.ecust.edu.cn:8000/predict/ site [5].

Supplementary Table S7: Physicochemical and toxicological parameters calculated for the selected compounds against the rapamycin binding site of mTOR based on molecular docking analysis. Each cluster groups compounds with structures with up to 70% structural similarity. Compounds whose name is CAS format belong to the Marine Natural Products database [1], compounds whose name begins with SN belong to the Super Natural II database [2], and compounds whose name begins with ZINC belong to the ZINC natural products [3]. Text in **bold** only for compounds tested *in vitro* in these paper.

Compound	Cluster 70%[a]	[a]Total Surface Area, Å^2	[a]TPSA, Å^2	cLogS[a]	MW[a]	cLogP[a]	HBA[a]	HBD[a]	Ro5 violations[a]	Druglikeness[a]	DrugScore[a]	RAT (LD50, mol/kg)[b]	Caco-2 Permeability (LogPapp, cm/s)[b]	TPT (pIGC50, μg/L)[b]	FT (pLC50, mg/L)[b]
1008752-06-4	1	430.3	92.7	-5.365	580.8	5.397	7	3	2	-2.153	0.13911	2.891	0.595	0.363	1.377
107900-75-4	1	430.3	92.7	-5.365	580.8	5.397	7	3	2	-2.153	0.13911	2.891	0.595	0.363	1.377
116477-23-7	1	423.5	54.5	-5.170	552.8	6.395	5	3	2	-1.962	0.13214	2.818	0.627	0.377	1.513
117631-50-2	1	423.9	72.5	-5.661	564.8	5.743	6	2	2	-2.153	0.12867	2.956	0.958	0.338	1.346
154466-37-2	1	425.3	75.6	-5.485	564.8	6.198	6	3	2	-2.232	0.12269	2.885	0.689	0.361	1.347
162465-80-7	1	422.5	54.9	-5.963	550.8	6.477	5	2	2	-1.687	0.14789	2.834	0.700	0.537	1.470
148439-45-6	1	315.1	51.4	-4.398	456.6	2.838	6	1	0	2.906	0.39595	2.936	1.452	0.477	1.127
SN00383086	1	382.3	59.1	-1.622	616.8	2.008	8	0	1	-1.202	0.35389	2.896	0.838	0.532	0.885
112693-24-0	2	291.7	31.9	-4.575	347.5	5.340	3	1	1	-4.613	0.21355	2.429	0.612	0.713	1.152
113831-00-8	3	276.4	118.9	-5.509	423.4	2.025	7	1	0	-2.627	0.30705	2.566	0.819	0.555	1.412
151247-69-7	3	284.9	115.0	-5.238	425.5	1.457	7	1	0	-1.649	0.35257	2.580	0.836	0.446	1.317
114571-91-4	4	383.9	107.5	-7.281	595.3	2.965	8	2	1	0.405	0.07853	2.631	0.375	0.700	1.023
135824-74-7	4	383.9	107.5	-7.281	595.3	2.965	8	2	1	0.405	0.07853	2.631	0.375	0.700	1.023
120314-15-0	4	340.0	72.9	-4.920	493.6	3.634	6	0	0	1.365	0.46583	2.671	1.050	0.418	0.751
120409-36-1	4	340.0	72.9	-4.920	493.6	3.634	6	0	0	1.365	0.46583	2.671	1.050	0.418	0.751
123853-69-0	4	341.9	90.0	-4.660	509.6	3.273	7	0	1	1.028	0.22449	2.642	1.129	0.458	0.753
164991-65-5	4	318.9	79.4	-4.500	481.6	3.071	6	1	0	2.770	0.27517	2.797	0.952	0.455	0.911

93426-90-5	4	338.0	72.9	-5.008	495.7	3.981	6	0	0	0.691	0.19654	2.671	1.161	0.464	0.700
123641-95-2	4	397.7	139.8	-1.863	566.6	1.652	11	1	2	1.909	0.35392	2.825	0.771	0.428	1.124
631913-68-3	4	418.1	163.5	-2.176	591.6	0.720	12	1	2	-2.454	0.19530	2.694	0.787	0.518	0.981
79664-61-2	4	427.4	128.8	-2.078	594.7	1.984	11	0	2	0.216	0.27573	2.706	0.892	0.453	1.136
**SN00111586**	**4**	**384.8**	**75.7**	**-3.812**	**516.7**	**4.127**	**6**	**1**	**1**	**1.523**	**0.49715**	**2.775**	**1.148**	**0.482**	**0.554**
184348-39-8	4	365.9	103.8	-4.193	508.7	2.819	7	2	1	4.554	0.58861	2.723	0.398	0.292	1.478
SN00108034	4	402.2	83.9	-5.435	551.1	5.209	6	1	2	-0.734	0.18048	2.742	0.829	0.723	0.888
SN00108118	4	446.4	83.9	-5.944	601.2	6.260	6	1	2	-0.483	0.16585	2.811	0.841	0.801	0.813
114995-72-1	5	404.0	94.5	-4.869	514.7	4.080	7	2	1	-5.750	0.14400	2.928	0.251	0.664	0.732
115267-16-8	5	404.0	94.5	-4.869	514.7	4.080	7	2	1	-5.750	0.14400	2.928	0.251	0.664	0.732
115268-43-4	5	411.7	97.8	-4.869	514.7	4.135	7	2	1	-4.611	0.08640	2.625	0.834	0.745	1.151
173792-58-0	5	395.3	68.1	-5.569	507.7	5.931	5	1	2	1.638	0.29615	2.668	0.844	0.732	0.982
118574-73-5	6	326.6	40.5	-5.772	414.7	6.297	2	2	1	-1.174	0.21439	2.937	1.626	0.933	0.618
145163-99-1	6	326.6	40.5	-5.772	414.7	6.297	2	2	1	-1.174	0.21439	2.937	1.626	0.933	0.618
465-95-2	6	338.1	60.7	-5.747	458.7	5.562	3	3	1	-4.206	0.18522	2.377	1.218	1.057	0.985
17991-67-2	6	345.3	94.8	-5.330	486.7	4.533	5	3	0	-1.702	0.25439	2.377	1.154	0.951	0.699
471-53-4	6	341.0	74.6	-5.780	470.7	5.356	4	2	1	-2.356	0.20001	2.377	1.154	0.951	0.699
564-16-9	6	337.0	57.5	-6.128	456.7	6.064	3	2	1	-3.051	0.16620	2.390	1.544	0.959	0.815
74984-66-0	6	337.9	57.5	-6.111	456.7	6.002	3	2	1	-3.658	0.16499	2.390	1.544	0.959	0.815
119212-28-1	7	419.4	108.6	-6.048	556.7	4.654	7	2	1	-2.651	0.11378	2.869	1.117	0.596	0.669
345642-84-4	7	398.0	119.5	-5.028	530.7	4.139	7	4	1	0.505	0.36350	2.976	0.742	0.496	1.044
52645-09-7	7	398.0	119.5	-5.028	530.7	4.139	7	4	1	0.505	0.36350	2.976	0.742	0.496	1.044
55945-74-9	7	398.0	119.5	-5.028	530.7	4.139	7	4	1	1.039	0.38925	2.976	0.742	0.496	1.044
65773-98-0	7	396.9	116.3	-5.079	528.6	4.108	7	3	1	-4.951	0.22532	2.976	0.742	0.496	1.044
122795-54-4	7	265.3	71.5	-6.325	385.4	3.277	6	1	0	5.488	0.50169	2.801	1.532	0.362	1.373
12626-18-5	7	420.4	102.7	-4.328	579.7	4.703	10	1	1	0.388	0.07198	2.962	1.117	0.634	0.639
12771-72-1	7	353.7	113.7	-3.464	511.6	2.606	10	2	1	0.324	0.10920	2.913	0.889	0.621	0.782
SN00239708	7	408.1	39.3	-6.639	614.8	4.937	6	0	1	0.278	0.12456	2.985	1.298	0.748	0.847
SN00250332	7	431.2	49.7	-7.608	616.8	6.110	6	1	2	0.986	0.17249	2.934	0.958	0.584	1.173
SN00257182	7	445.8	103.5	-6.383	670.8	2.785	9	2	1	4.064	0.15131	2.973	0.999	0.550	0.821

SN00278770	7	408.1	39.3	-6.639	614.8	4.937	6	0	1	0.278	0.12456	2.985	1.298	0.748	0.847
SN00288395	7	415.3	60.7	-7.480	602.8	3.829	6	2	1	1.984	0.27340	2.794	0.483	0.462	1.546
SN00348164	7	445.8	103.5	-6.383	670.8	2.785	9	2	1	4.064	0.15131	2.973	0.999	0.550	0.821
SN00352327	7	436.3	70.0	-7.209	632.8	5.088	7	2	2	0.001	0.17800	2.929	0.973	0.507	1.462
140715-85-1	7	303.8	114.3	-3.507	445.5	0.684	9	3	0	6.959	0.74762	2.453	0.150	0.409	1.413
140715-87-3	7	324.0	123.6	-3.318	475.5	0.701	10	3	0	7.536	0.71976	2.434	0.391	0.556	1.248
140852-71-7	7	303.8	114.3	-3.507	445.5	0.684	9	3	0	6.959	0.74762	2.453	0.150	0.409	1.413
159518-77-1	7	317.6	139.3	-5.454	492.6	3.889	7	3	0	5.697	0.46213	2.664	1.379	0.582	1.442
524067-24-1	7	372.9	126.4	-2.899	587.5	2.229	10	4	1	3.578	0.56994	2.658	0.863	0.719	1.458
524067-25-2	7	354.3	126.4	-2.065	508.6	1.504	10	4	1	5.368	0.70633	2.739	0.901	0.510	1.554
524067-26-3	7	351.7	119.6	-2.885	509.6	1.903	10	3	1	3.772	0.67337	2.631	1.103	0.539	1.615
61897-88-9	7	311.9	108.8	-4.005	460.4	0.597	10	0	0	0.340	0.19817	2.484	1.072	0.583	0.974
61897-89-0	7	273.0	91.3	-3.941	402.4	0.997	8	1	0	4.316	0.75625	2.489	0.867	0.378	1.148
61897-90-3	7	301.9	99.6	-3.861	444.4	1.307	9	0	0	3.727	0.42741	2.445	0.990	0.421	0.989
61949-67-5	7	278.2	102.8	-3.719	418.4	0.337	9	1	0	2.257	0.72972	2.538	0.860	0.522	1.178
66212-51-9	7	273.0	91.3	-3.941	402.4	0.997	8	1	0	4.316	0.75625	2.489	0.867	0.378	1.148
SN00037381	7	459.8	122.9	-6.472	664.8	2.633	10	4	1	5.511	0.31784	2.553	0.451	0.451	1.579
SN00037382	7	459.8	122.9	-6.472	664.8	2.633	10	4	1	5.511	0.31784	2.553	0.451	0.451	1.579
SN00091501	7	346.7	78.5	-5.549	471.6	3.448	6	2	0	-0.716	0.32793	2.358	0.610	0.517	1.274
SN00097411	7	345.8	82.1	-5.987	490.6	3.559	7	1	0	3.197	0.43069	2.638	1.183	0.643	1.231
**SN00097418**	**7**	**332.0**	**82.1**	**-5.717**	**476.5**	**3.105**	**7**	**1**	**0**	**2.776**	**0.47292**	**2.628**	**1.460**	**0.622**	**1.317**
SN00097526	7	354.3	91.3	-5.735	506.6	3.035	8	1	1	2.849	0.44733	2.512	1.512	0.655	0.907
SN00097687	7	356.5	82.1	-6.405	504.6	3.792	7	1	1	2.776	0.22923	2.602	1.540	0.678	1.227
SN00097688	7	356.5	82.1	-6.405	504.6	3.792	7	1	1	2.776	0.22923	2.602	1.540	0.678	1.227
SN00097689	7	356.5	82.1	-6.405	504.6	3.792	7	1	1	2.776	0.22923	2.602	1.540	0.678	1.227
SN00097956	7	354.3	91.3	-5.735	506.6	3.035	8	1	1	2.849	0.44733	2.540	1.390	0.598	0.951
SN00098042	7	356.5	82.1	-6.405	504.6	3.792	7	1	1	2.776	0.13754	2.602	1.540	0.678	1.227
SN00098043	7	356.5	82.1	-6.405	504.6	3.792	7	1	1	2.776	0.13754	2.602	1.540	0.678	1.227
SN00098044	7	356.5	82.1	-6.405	504.6	3.792	7	1	1	2.776	0.13754	2.602	1.540	0.678	1.227
SN00109120	7	362.1	97.0	-6.124	530.0	3.287	8	2	1	-2.718	0.17234	2.371	0.493	0.626	1.044

SN00109705	7	361.2	91.7	-7.410	517.6	3.395	7	2	1	5.690	0.21499	2.353	0.986	0.362	1.323
SN00109757	7	364.3	91.7	-7.802	538.0	3.657	7	2	1	5.735	0.19700	2.308	0.936	0.620	1.195
SN00111609	7	368.0	91.3	-6.005	520.6	3.489	8	1	1	3.257	0.40710	2.565	1.138	0.625	0.958
SN00111617	7	387.2	111.2	-6.329	547.6	3.263	9	2	1	3.942	0.08144	2.529	0.838	0.689	1.213
SN00111618	7	387.2	111.2	-6.329	547.6	3.263	9	2	1	3.942	0.08144	2.529	0.838	0.689	1.213
SN00111625	7	372.0	108.4	-6.017	534.6	3.091	9	1	1	2.706	0.24045	2.520	1.277	0.644	0.808
SN00111652	7	370.3	82.1	-6.675	518.6	4.247	7	1	1	3.197	0.20655	2.643	1.267	0.683	1.168
SN00111663	7	369.9	119.4	-6.000	534.6	0.981	9	2	1	3.154	0.43051	2.484	0.524	0.602	1.233
SN00111665	7	369.9	119.4	-6.000	534.6	0.981	9	2	1	3.154	0.43051	2.484	0.524	0.602	1.233
SN00111673	7	375.8	99.2	-6.671	532.6	3.430	8	1	1	3.037	0.21774	2.555	1.189	0.679	1.063
SN00111674	7	375.8	99.2	-6.671	532.6	3.430	8	1	1	3.037	0.21774	2.555	1.189	0.679	1.063
SN00111675	7	375.8	99.2	-6.671	532.6	3.430	8	1	1	3.037	0.21774	2.555	1.189	0.679	1.063
SN00111676	7	375.8	99.2	-6.671	532.6	3.430	8	1	1	3.037	0.21774	2.555	1.189	0.679	1.063
SN00111701	7	362.0	99.2	-6.401	518.6	2.976	8	1	1	2.622	0.23609	2.507	1.442	0.668	1.076
SN00111702	7	362.0	99.2	-6.401	518.6	2.976	8	1	1	2.622	0.23609	2.507	1.442	0.668	1.076
SN00111703	7	362.0	99.2	-6.401	518.6	2.976	8	1	1	2.622	0.23609	2.507	1.442	0.668	1.076
SN00111704	7	362.0	99.2	-6.401	518.6	2.976	8	1	1	2.622	0.23609	2.507	1.442	0.668	1.076
SN00111711	7	373.5	111.2	-6.059	533.6	2.808	9	2	1	3.553	0.08907	2.462	1.116	0.707	1.259
SN00111712	7	373.5	111.2	-6.059	533.6	2.808	9	2	1	3.553	0.08907	2.462	1.116	0.707	1.259
SN00226829	7	454.3	122.9	-6.777	684.7	2.802	10	4	1	-1.525	0.17598	2.667	0.558	0.691	1.400
SN00226941	7	472.1	122.9	-6.816	678.8	2.977	10	4	1	5.455	0.29582	2.563	0.509	0.493	1.622
SN00233772	7	467.9	122.9	-6.933	670.8	2.989	10	4	1	0.402	0.23603	2.593	0.254	0.467	1.483
SN00243155	7	472.1	122.9	-6.816	678.8	2.977	10	4	1	5.455	0.29582	2.563	0.509	0.493	1.622
SN00253708	7	466.2	122.9	-6.786	682.8	2.734	10	4	1	4.171	0.29753	2.568	0.313	0.687	1.365
SN00260522	7	459.8	122.9	-6.472	664.8	2.633	10	4	1	5.511	0.31784	2.553	0.451	0.451	1.579
SN00261089	7	472.1	122.9	-6.816	678.8	2.977	10	4	1	5.455	0.17749	2.645	0.492	0.501	1.649
SN00261527	7	484.4	122.9	-7.160	692.8	3.321	10	4	1	5.455	0.16587	2.666	0.469	0.517	1.617
SN00267944	7	454.7	124.6	-6.691	630.7	3.465	10	4	1	5.435	0.30801	2.573	0.182	0.514	1.448
SN00290152	7	394.5	55.9	-7.264	614.8	5.315	6	1	2	3.065	0.08223	2.563	1.329	0.690	1.193
SN00297098	7	466.2	122.9	-6.786	682.8	2.734	10	4	1	4.171	0.29753	2.568	0.313	0.687	1.365

SN00304654	7	466.2	122.9	-6.786	682.8	2.734	10	4	1	4.171	0.29753	2.568	0.313	0.687	1.365
SN00305691	7	394.5	55.9	-7.264	614.8	5.315	6	1	2	3.065	0.13706	2.563	1.329	0.690	1.193
SN00310110	7	484.4	122.9	-7.160	692.8	3.321	10	4	1	5.455	0.27645	2.572	0.495	0.519	1.589
SN00318192	7	472.1	122.9	-6.816	678.8	2.977	10	4	1	5.455	0.29582	2.611	0.558	0.502	1.544
SN00322991	7	472.1	122.9	-6.816	678.8	2.977	10	4	1	5.455	0.29582	2.563	0.509	0.493	1.622
SN00323189	7	482.0	105.3	-5.748	692.8	3.139	10	2	1	5.337	0.33501	2.738	0.596	0.530	1.492
SN00327455	7	482.0	105.3	-5.748	692.8	3.139	10	2	1	5.337	0.33501	2.738	0.596	0.530	1.492
SN00330261	7	466.2	122.9	-6.786	682.8	2.734	10	4	1	4.171	0.29753	2.568	0.313	0.687	1.365
SN00331859	7	455.8	151.1	-6.482	670.8	2.499	10	4	1	7.229	0.31746	2.442	0.518	0.498	1.601
SN00340494	7	437.4	67.3	-6.513	650.8	4.010	7	1	1	2.712	0.16918	3.037	0.995	0.455	0.952
SN00341689	7	484.4	122.9	-7.160	692.8	3.321	10	4	1	5.455	0.27645	2.572	0.495	0.519	1.589
SN00344654	7	484.4	122.9	-7.160	692.8	3.321	10	4	1	5.455	0.27645	2.627	0.551	0.527	1.509
SN00350405	7	466.2	122.9	-6.786	682.8	2.734	10	4	1	4.171	0.29753	2.568	0.313	0.687	1.365
SN00354912	7	472.5	122.9	-7.100	700.7	2.834	10	4	1	4.171	0.28254	2.568	0.313	0.687	1.365
SN00356104	7	472.5	122.9	-7.100	700.7	2.834	10	4	1	4.171	0.28254	2.568	0.313	0.687	1.365
SN00371872	7	466.2	122.9	-6.786	682.8	2.734	10	4	1	4.171	0.29753	2.568	0.313	0.687	1.365
SN00379189	7	475.3	122.9	-7.208	699.2	3.239	10	4	1	5.544	0.27544	2.484	0.392	0.743	1.307
SN00379780	7	475.3	122.9	-7.208	699.2	3.239	10	4	1	5.544	0.27544	2.484	0.392	0.743	1.307
SN00383686	7	472.5	122.9	-7.100	700.7	2.834	10	4	1	4.171	0.28254	2.568	0.313	0.687	1.365
SN00390671	7	424.6	122.9	-5.816	614.7	1.914	10	4	1	2.821	0.37313	2.619	0.588	0.541	1.310
SN00393875	7	472.5	122.9	-7.100	700.7	2.834	10	4	1	4.171	0.28254	2.568	0.313	0.687	1.365
169626-35-1	7	261.4	77.6	-3.702	358.4	1.846	6	2	0	6.152	0.80604	2.742	0.688	0.302	1.809
749216-47-5	7	261.9	85.5	-2.575	384.4	1.057	7	2	0	3.167	0.83799	2.948	1.359	0.667	1.225
SN00004671	7	375.1	108.0	-5.783	520.0	2.782	8	3	1	6.406	0.09708	2.336	0.483	0.583	1.241
SN00007650	7	342.3	78.5	-6.276	485.5	3.725	6	2	0	-3.519	0.07754	2.454	0.864	0.597	1.427
SN00008385	7	335.0	79.0	-4.726	459.5	2.838	7	1	0	4.798	0.21584	2.361	0.629	0.514	1.175
SN00008387	7	358.1	79.0	-5.190	509.5	3.585	7	1	1	-1.052	0.10926	2.437	0.705	0.565	1.184
SN00015950	7	331.2	78.5	-6.240	461.5	3.520	6	2	0	5.002	0.16079	2.338	0.812	0.546	1.441
SN00132224	7	326.1	80.8	-6.822	473.5	3.829	6	0	0	3.149	0.08388	2.299	1.330	0.563	0.592
SN00133950	7	351.3	80.8	-7.302	499.5	4.293	6	0	0	3.149	0.07232	2.299	1.330	0.563	0.592

SN00134172	7	351.3	80.8	-7.302	499.5	4.293	6	0	0	3.149	0.07232	2.299	1.330	0.563	0.592
SN00141410	7	289.7	57.7	-5.931	408.5	3.206	5	0	0	4.079	0.11164	2.244	1.607	0.627	0.896
SN00259728	7	364.8	75.6	-5.023	463.6	5.156	5	2	1	-1.969	0.24622	3.009	0.398	0.602	0.828
SN00319706	7	483.3	156.9	-4.934	627.7	2.851	11	5	2	5.612	0.42236	2.420	0.463	0.542	1.186
**ZINC08918463**	**7**	**272.4**	**34.1**	**-4.161**	**445.4**	**2.311**	**5**	**1**	**0**	**-1.751**	**0.39121**	**2.822**	**0.561**	**0.463**	**1.409**
ZINC13595929	7	305.5	38.3	-4.149	430.6	1.848	5	2	0	2.709	0.68469	3.003	0.990	0.407	1.113
ZINC00626315	7	326.2	34.0	-3.981	420.6	5.889	4	1	1	-0.420	0.33662	2.586	0.935	0.539	1.426
ZINC00754173	7	340.0	34.0	-4.251	434.6	6.231	4	1	1	-0.628	0.29247	2.569	0.977	0.542	1.359
ZINC00754174	7	340.0	34.0	-4.251	434.6	6.231	4	1	1	-0.628	0.29247	2.569	0.977	0.542	1.359
122540-32-3	8	350.5	83.8	-4.195	402.5	5.102	5	2	1	-8.705	0.27481	2.168	0.599	0.415	1.770
133805-03-5	9	237.0	118.2	-5.447	357.3	2.762	7	5	0	1.524	0.55632	2.597	0.231	0.153	1.289
135213-04-6	10	367.1	98.8	-3.428	488.6	1.889	8	2	0	7.247	0.68595	2.201	0.194	0.305	1.680
135340-00-0	11	296.5	79.3	-5.752	429.5	2.940	6	2	0	3.173	0.51873	2.621	0.744	0.645	1.484
SN00281717	11	359.2	117.1	-7.142	522.5	2.432	8	2	1	1.650	0.35101	2.380	0.525	0.823	0.930
139083-13-9	12	311.6	90.6	-7.574	471.3	4.608	6	4	0	2.392	0.32460	2.439	0.146	0.727	1.180
140429-37-4	12	305.1	99.5	-6.743	441.5	3.159	7	4	0	5.165	0.44521	2.529	0.358	0.393	1.398
139220-18-1	13	460.5	123.0	-3.964	555.8	6.141	7	1	2	1.381	0.19497	2.558	0.691	0.455	1.005
139594-87-9	14	481.4	139.6	-5.411	616.8	4.944	9	3	1	-4.121	0.16102	2.928	0.216	0.650	1.222
6758-71-0	14	334.4	55.8	-5.627	468.7	4.759	4	1	0	-4.133	0.12760	3.003	1.068	0.894	0.603
SN00162890	14	331.9	93.1	-4.934	496.6	2.795	6	2	0	2.689	0.52676	2.488	0.865	0.203	0.970
SN00344013	14	333.4	114.4	-4.471	526.6	0.753	9	0	1	-2.992	0.30107	2.609	1.085	0.609	0.507
148149-82-0	14	261.3	46.5	-3.862	318.5	5.247	3	1	1	-6.927	0.18013	2.399	1.376	0.472	1.012
152110-09-3	14	322.3	71.1	-5.386	460.6	4.051	6	0	0	-5.411	0.24523	2.291	0.891	1.118	0.656
152186-77-1	14	322.3	71.1	-5.386	460.6	4.051	6	0	0	-5.411	0.24523	2.291	0.891	1.118	0.656
79849-37-9	14	349.0	91.3	-5.577	516.7	3.229	7	1	1	-4.368	0.13884	2.988	0.825	1.135	0.965
858950-34-2	14	387.8	117.6	-5.588	574.7	2.862	9	1	1	-4.453	0.12579	2.988	0.825	1.135	0.965
SN00083037	14	326.4	43.4	-6.525	454.7	5.890	3	0	1	-3.184	0.16147	2.154	1.365	0.856	0.545
SN00352197	14	327.5	46.5	-6.406	456.7	5.774	3	1	1	-6.034	0.09630	2.106	1.319	0.918	1.474
199165-88-3	14	287.7	66.8	-4.419	402.6	3.580	4	2	0	-7.188	0.32673	2.303	0.714	0.902	0.814
32450-26-3	14	348.8	89.9	-4.826	472.6	3.643	6	1	0	1.980	0.51450	2.162	1.249	1.007	0.626

71103-05-4	14	307.0	63.6	-5.148	426.6	4.422	4	1	0	-5.599	0.25703	2.679	1.350	0.818	0.511
674819-46-6	14	306.2	29.5	-5.880	398.6	6.124	2	1	1	0.171	0.27502	2.196	1.495	1.052	0.514
SN00329349	14	356.9	77.3	-3.556	484.6	3.799	6	0	0	0.250	0.48306	3.036	1.602	0.710	0.573
141266-06-0	15	324.9	57.6	-4.614	435.6	4.780	4	1	0	1.649	0.48008	2.762	0.942	0.581	0.707
14270-73-6	16	351.3	88.1	-4.099	470.5	2.818	7	2	0	-1.719	0.23766	2.071	0.727	0.527	1.371
14325-03-2	17	435.2	127.1	-6.473	562.7	5.155	8	4	2	-2.052	0.15939	2.566	0.379	0.550	1.158
151484-78-5	17	364.1	89.8	-5.699	466.6	4.429	6	3	0	0.654	0.36172	2.556	0.716	0.603	1.421
147395-97-9	18	353.7	114.5	-4.055	488.5	2.491	11	1	1	5.147	0.63320	2.527	0.994	0.488	1.145
149764-31-8	19	468.0	153.2	-6.515	694.9	4.562	9	2	1	-5.327	0.12965	2.423	0.382	1.008	0.851
149764-33-0	19	429.4	101.4	-7.191	614.9	6.178	6	2	2	-3.517	0.10502	2.444	0.350	0.963	0.897
149764-34-1	20	487.4	144.0	-6.079	678.9	6.146	8	2	2	-5.888	0.10773	2.339	0.496	1.154	0.526
151890-81-2	21	404.5	79.2	-6.000	530.8	5.914	5	3	2	-5.397	0.14435	3.009	0.146	0.985	0.950
211358-65-5	21	408.7	99.4	-5.550	546.8	5.090	6	4	2	-4.456	0.17391	3.009	0.146	0.985	0.950
329050-20-6	21	361.1	66.8	-5.636	474.7	5.581	4	2	1	-2.383	0.19528	2.790	1.180	0.724	0.855
74185-11-8	21	404.5	79.2	-6.000	530.8	5.914	5	3	2	-5.397	0.14435	3.009	0.146	0.985	0.950
78518-73-7	21	362.2	69.9	-5.585	476.7	5.426	4	3	1	-1.348	0.22409	2.824	1.025	0.790	0.879
86748-29-0	21	362.2	69.9	-5.585	476.7	5.438	4	3	1	-1.201	0.22832	2.824	1.025	0.790	0.879
86748-30-3	21	365.7	87.0	-5.201	490.7	4.888	5	3	0	-1.002	0.26835	2.790	1.180	0.724	0.855
86783-84-8	21	362.2	69.9	-5.585	476.7	5.426	4	3	1	-1.348	0.22409	2.824	1.025	0.790	0.879
16250-61-6	21	326.9	53.0	-5.368	428.7	5.346	3	2	1	-0.784	0.16705	2.422	1.486	0.833	0.817
208708-22-9	21	275.3	66.8	-4.269	388.5	3.512	4	2	0	-1.655	0.39531	2.120	1.191	0.890	0.555
208708-24-1	21	271.3	49.7	-4.617	374.6	4.220	3	2	0	-2.866	0.32192	2.094	1.216	0.914	0.736
233607-69-7	21	358.7	65.0	-5.581	488.7	5.173	5	1	1	-2.045	0.21150	2.440	1.043	1.005	0.712
86363-50-0	21	322.8	38.7	-5.725	426.6	6.281	3	1	1	-2.047	0.11516	2.813	1.339	0.731	0.807
191212-37-0	21	370.7	76.0	-5.620	490.7	4.937	5	2	0	-3.905	0.19923	2.942	1.248	0.992	0.538
205750-25-0	21	359.6	61.8	-5.867	488.7	4.646	5	0	0	-3.580	0.20256	2.530	1.268	0.625	1.141
452934-96-2	21	370.7	76.0	-5.620	490.7	4.937	5	2	0	-3.641	0.20037	2.653	1.226	0.965	0.528
487016-98-8	21	360.7	65.0	-5.816	490.7	4.503	5	1	0	-2.378	0.21953	2.797	1.249	0.645	1.471
858950-55-7	21	318.6	76.0	-5.262	460.7	3.735	5	2	0	-1.393	0.18554	3.033	0.927	0.842	1.488
2061-64-5	21	324.4	38.7	-5.989	428.7	6.386	3	1	1	-2.464	0.10498	2.941	1.343	0.931	1.229

256377-68-1	21	323.1	38.7	-6.353	442.7	6.317	3	1	1	-2.926	0.09584	2.883	1.424	0.910	1.147
40071-60-1	21	324.4	38.7	-5.989	428.7	6.386	3	1	1	-2.464	0.10498	2.941	1.343	0.931	1.229
75179-58-7	21	299.6	38.7	-5.559	400.6	5.713	3	1	1	-0.530	0.16459	2.941	1.343	0.931	1.229
263764-04-1	21	331.9	70.1	-5.092	442.6	4.296	4	2	0	-0.157	0.29991	2.749	1.472	1.025	0.974
263764-05-2	21	333.0	70.1	-5.320	444.7	4.548	4	2	0	-6.898	0.19018	2.919	1.316	1.175	0.757
94806-03-8	21	326.8	49.8	-5.491	426.6	5.148	3	1	1	-1.438	0.20531	2.765	1.795	1.096	0.756
SN00231454	21	394.4	90.4	-5.186	563.7	4.484	7	1	1	-3.449	0.10274	3.007	1.213	0.994	0.656
SN00298985	21	394.4	90.4	-5.186	563.7	4.484	7	1	1	-3.449	0.10274	3.007	1.213	0.994	0.656
155645-51-5	22	487.8	120.0	-5.839	604.7	5.090	9	2	2	4.157	0.29143	2.616	0.380	0.473	1.086
186593-86-2	22	478.9	143.6	-5.500	627.1	4.154	10	3	1	1.973	0.19434	2.668	0.692	0.675	1.053
244157-96-8	22	430.4	145.3	-3.788	566.1	2.805	10	4	1	5.786	0.55061	2.631	0.480	0.592	1.203
157207-88-0	23	296.3	31.1	-3.932	351.5	5.019	3	2	1	-3.380	0.18748	2.445	0.347	0.622	1.346
158758-41-9	24	317.3	108.4	-6.574	430.5	3.104	6	3	0	-1.559	0.09817	2.463	1.210	0.491	1.507
159934-14-2	25	365.0	35.8	-5.154	466.8	6.006	3	1	1	3.202	0.36296	2.770	1.036	0.611	0.815
159934-15-3	25	371.6	61.7	-5.798	480.7	5.797	4	2	1	0.560	0.27362	2.746	0.690	0.463	0.989
454476-89-2	25	336.7	38.1	-6.184	426.7	5.895	2	2	1	-1.196	0.20928	2.570	0.795	0.698	0.864
640734-87-8	25	346.8	29.3	-5.594	438.7	5.873	2	1	1	-0.545	0.25131	2.759	0.971	0.560	0.655
175170-89-5	26	263.9	116.2	-6.899	366.4	2.813	7	4	0	1.292	0.43869	2.447	0.654	0.317	1.843
94935-97-4	26	290.2	93.5	-6.859	394.4	3.387	7	3	0	2.735	0.44898	2.596	1.214	0.389	1.529
188558-50-1	27	315.9	115.6	-4.378	404.5	3.191	8	4	0	-1.700	0.23369	2.469	0.945	0.410	1.546
20829-55-4	28	278.8	89.8	-4.140	372.4	1.917	6	4	0	4.423	0.75414	2.416	0.344	0.091	1.984
244066-05-5	28	337.9	78.9	-4.913	440.5	3.185	6	3	0	1.452	0.53518	2.834	0.452	0.461	1.334
2122-98-7	29	362.3	136.3	-6.620	496.6	5.280	9	2	1	-2.986	0.16488	2.652	0.687	0.896	0.808
2497-74-7	29	337.5	136.3	-6.190	468.6	4.901	9	2	0	-3.146	0.19497	2.665	0.604	0.824	0.873
2643-02-9	29	366.5	156.5	-6.170	512.6	4.457	10	3	1	-2.057	0.16436	2.669	0.589	0.822	0.849
6891-35-6	29	337.5	136.3	-6.190	468.6	4.901	9	2	0	-2.772	0.11896	2.665	0.604	0.824	0.873
220503-29-7	30	262.2	109.1	-4.806	389.4	0.196	8	1	0	4.726	0.68721	2.624	0.510	0.508	1.462
221367-90-4	31	447.0	114.9	-6.297	580.7	6.321	8	4	2	-1.327	0.14177	2.688	0.290	0.643	1.129
474779-75-4	32	284.3	40.0	-6.287	476.2	3.417	4	1	0	3.641	0.34513	2.893	0.822	0.917	1.328
50909-86-9	33	309.8	64.9	-6.010	407.5	4.415	5	2	0	4.183	0.45231	2.653	0.931	0.385	1.548

639512-19-9	34	396.0	104.2	-6.676	530.6	6.296	8	0	2	-4.603	0.07506	2.281	1.188	0.925	0.990
65527-04-0	35	339.4	57.5	-6.375	458.7	6.168	3	2	1	-2.039	0.16802	2.836	1.318	0.984	1.494
65527-05-1	35	339.4	57.5	-6.375	458.7	6.168	3	2	1	-2.039	0.16802	2.836	1.318	0.984	1.494
80442-78-0	36	319.7	79.2	-5.211	462.7	3.591	5	3	0	-2.102	0.17479	3.016	0.758	0.904	1.551
80442-79-1	36	319.7	79.2	-5.211	462.7	3.591	5	3	0	-2.102	0.17479	3.016	0.758	0.904	1.551
80442-84-8	36	387.1	91.3	-6.031	546.7	4.560	7	1	1	-2.106	0.11510	2.917	0.754	1.135	0.910
858950-46-6	36	339.0	83.5	-5.491	516.7	3.147	7	1	1	-2.553	0.14999	2.623	0.970	1.106	0.891
858950-48-8	36	339.0	83.5	-5.491	516.7	3.147	7	1	1	-2.553	0.14999	2.861	0.974	1.078	1.007
86047-14-5	37	220.5	63.2	-3.617	313.3	1.398	5	0	0	2.172	0.80186	2.331	1.503	0.210	1.746
866403-75-0	38	293.9	76.0	-4.812	426.6	3.782	5	2	0	0.304	0.27639	2.694	0.965	1.142	0.864
SN00005498	39	379.7	74.7	-5.727	501.6	5.017	6	1	2	5.821	0.37008	2.614	0.551	0.349	1.542
SN00012590	39	329.7	49.7	-5.164	427.6	5.474	4	0	1	-1.131	0.16053	2.719	0.873	0.544	1.720
SN00012599	39	341.2	61.8	-5.591	442.6	5.415	5	1	1	-0.424	0.27549	2.403	0.718	0.475	1.862
SN00012601	39	339.0	61.8	-5.562	454.5	5.179	5	1	1	4.075	0.40158	2.513	1.018	0.553	1.486
SN00105976	39	480.3	123.5	-5.465	632.8	5.414	10	2	2	2.577	0.17477	2.447	0.339	0.384	1.810
SN00052010	39	408.7	83.8	-6.623	523.7	4.525	7	1	1	2.228	0.32009	2.654	0.817	0.496	1.170
SN00078665	39	388.9	57.9	-5.136	526.1	4.647	6	0	1	5.174	0.41259	2.642	0.950	0.659	1.187
**SN00078968**	**39**	**375.1**	**57.9**	**-5.131**	**512.0**	**4.391**	**6**	**0**	**1**	**5.212**	**0.44120**	**2.631**	**0.938**	**0.656**	**1.154**
SN00015562	40	379.7	71.8	-5.624	478.6	3.875	6	2	0	1.776	0.25753	2.516	0.536	0.479	1.587
ZINC12753676	40	280.6	69.0	-4.028	423.5	1.863	8	2	0	-1.617	0.33768	2.592	0.922	0.570	1.402
SN00056789	41	333.1	51.5	-5.707	451.5	4.774	5	0	0	-1.075	0.09591	2.680	1.308	0.570	0.605
SN00057684	41	341.5	51.5	-5.375	461.5	4.664	5	0	0	2.723	0.15679	2.703	1.318	0.524	0.681
SN00059271	41	364.1	51.5	-5.950	487.6	5.028	5	0	1	2.810	0.12834	2.683	1.144	0.467	0.817
SN00059388	41	331.1	51.5	-5.251	447.5	4.224	5	0	0	2.570	0.17371	2.684	1.386	0.594	0.638
SN00059442	41	330.3	51.5	-5.597	451.5	4.702	5	0	0	1.283	0.14044	2.600	1.036	0.500	0.770
SN00058256	41	344.1	51.5	-5.867	465.6	5.044	5	0	1	-1.048	0.08761	2.682	1.233	0.570	0.642
SN00066079	42	344.0	95.9	-8.876	494.5	4.970	7	1	0	2.751	0.10182	2.414	1.065	0.489	1.307
SN00074793	43	330.0	117.4	-6.428	470.4	3.216	8	1	0	2.407	0.20231	2.569	1.040	0.554	1.029
SN00080932	44	375.2	67.7	-7.223	520.6	5.192	7	0	2	5.874	0.22899	2.574	0.909	0.478	1.114
SN00080933	44	375.2	67.7	-7.223	520.6	5.192	7	0	2	5.874	0.22899	2.574	0.909	0.478	1.114

SN00080974	44	368.8	67.7	-6.909	502.6	5.092	7	0	2	7.214	0.24880	2.538	1.021	0.368	1.203
SN00080956	44	359.4	73.2	-7.562	475.5	5.554	6	1	1	1.410	0.20749	2.369	0.618	0.177	1.485
SN00097226	44	370.1	99.2	-7.380	497.6	4.745	7	2	0	5.274	0.25494	2.791	0.573	0.486	1.365
SN00097227	44	370.1	99.2	-7.380	497.6	4.745	7	2	0	5.274	0.25494	2.791	0.573	0.486	1.365
SN00080936	45	358.6	58.4	-7.463	486.6	5.899	6	0	1	6.806	0.12842	2.578	1.105	0.347	1.249
SN00080939	45	334.1	58.4	-6.775	458.5	5.211	6	0	1	6.920	0.26864	2.390	0.997	0.359	1.394
SN00081043	45	349.5	58.4	-7.511	493.0	5.817	6	0	1	6.906	0.21352	2.396	0.882	0.642	1.262
SN00081067	45	340.4	58.4	-7.089	476.5	5.312	6	0	1	5.580	0.24749	2.432	0.951	0.626	1.147
SN00081079	45	340.4	58.4	-7.089	476.5	5.312	6	0	1	5.580	0.24749	2.427	0.802	0.525	1.246
SN00097206	45	374.3	58.4	-6.770	498.6	5.895	6	0	1	8.099	0.22226	2.500	0.698	0.394	1.241
SN00097225	45	347.8	58.4	-6.253	472.5	5.134	6	0	1	7.810	0.28342	2.367	0.635	0.261	1.548
SN00097229	45	368.1	76.9	-6.964	516.6	5.246	8	0	2	7.543	0.23433	2.212	0.728	0.280	1.238
SN00111460	45	363.5	58.4	-7.553	526.5	6.060	6	0	2	-0.270	0.13727	2.545	0.878	0.603	1.249
SN00111540	45	412.6	95.4	-7.000	576.6	5.106	10	0	2	7.487	0.21189	2.322	1.013	0.368	1.041
**SN00081767**	**45**	**341.5**	**88.7**	**-6.064**	**476.5**	**4.405**	**8**	**0**	**0**	**6.606**	**0.32421**	**2.254**	**0.707**	**0.259**	**1.403**
SN00081539	46	354.9	93.1	-2.607	472.5	2.567	7	0	0	4.907	0.72448	2.600	0.633	0.414	0.873
SN00081619	46	375.4	93.1	-2.874	508.6	3.029	7	0	1	4.504	0.65160	2.766	0.911	0.458	0.536
SN00081634	46	363.1	93.1	-2.813	488.6	3.434	7	0	0	3.522	0.65593	2.758	0.785	0.539	0.596
SN00081635	46	355.5	93.1	-2.644	470.5	2.302	7	0	0	3.212	0.72119	2.523	0.442	0.513	0.859
SN00081734	46	380.1	93.1	-3.439	514.6	3.825	7	0	1	-0.875	0.37958	2.768	0.688	0.493	0.591
SN00081740	46	366.4	93.1	-3.169	500.6	3.370	7	0	1	-0.847	0.41618	2.759	0.798	0.497	0.502
SN00089681	46	355.9	93.1	-3.045	486.6	2.930	7	0	0	-0.522	0.46789	2.770	0.756	0.483	0.528
SN00089691	46	352.6	93.1	-2.689	474.6	2.994	7	0	0	3.840	0.70030	2.758	0.785	0.539	0.596
SN00089693	46	369.7	93.1	-3.315	500.6	3.384	7	0	1	-0.551	0.43240	2.776	0.657	0.486	0.618
SN00091326	46	360.0	113.3	-2.826	486.5	2.377	8	1	0	4.716	0.70299	2.556	0.512	0.537	1.030
SN00101331	46	399.7	102.3	-3.200	528.6	2.861	8	0	1	3.746	0.36931	2.637	0.766	0.462	0.976
SN00111395	46	396.8	93.1	-3.376	536.6	3.794	7	0	1	4.651	0.56237	2.794	1.013	0.532	0.517
SN00111397	46	410.6	93.1	-3.646	550.7	4.249	7	0	1	3.575	0.50267	2.773	0.865	0.565	0.616
SN00111415	46	386.4	93.1	-3.252	522.6	3.354	7	0	1	4.971	0.60749	2.798	0.916	0.485	0.526
SN00111420	46	385.8	93.1	-2.998	522.6	3.470	7	0	1	4.179	0.60912	2.797	0.852	0.464	0.608

SN00111437	46	399.6	93.1	-3.268	536.6	3.924	7	0	1	3.105	0.54974	2.741	0.801	0.515	0.595
SN00111564	46	389.1	93.1	-3.144	522.6	3.484	7	0	1	3.429	0.59823	2.746	0.761	0.514	0.624
SN00111571	46	400.1	93.1	-3.522	536.6	3.809	7	0	1	3.894	0.55219	2.776	0.765	0.523	0.609
SN00111576	46	390.8	102.3	-3.617	532.6	3.285	8	0	1	-3.334	0.24130	2.675	0.708	0.659	0.752
SN00096066	46	332.7	46.6	-4.296	434.5	4.359	4	0	0	2.150	0.55207	2.731	1.296	0.467	0.915
**SN00082651**	**47**	**357.7**	**83.9**	**-3.794**	**486.6**	**3.323**	**6**	**1**	**0**	**1.613**	**0.57553**	**3.064**	**1.020**	**0.475**	**0.649**
SN00084678	47	408.4	83.9	-5.844	540.7	5.341	6	1	2	0.973	0.06315	2.988	0.900	0.580	0.794
ZINC06232954	47	300.1	79.6	-4.921	423.5	2.425	6	1	0	-9.721	0.18853	2.879	1.053	0.424	0.968
ZINC06232955	47	300.1	79.6	-4.921	423.5	2.425	6	1	0	-9.721	0.18853	2.879	1.053	0.424	0.968
SN00106774	48	390.6	105.0	-4.431	537.6	3.764	7	1	1	2.570	0.48702	2.602	1.259	0.541	0.857
SN00106779	49	459.2	125.3	-4.893	602.8	4.934	8	3	1	0.984	0.30645	3.026	0.844	0.628	1.026
SN00107651	50	368.4	129.6	-5.793	499.6	2.290	7	1	0	3.826	0.30194	2.562	0.906	0.619	1.170
SN00111466	51	391.6	78.2	-8.680	536.6	6.346	7	1	2	2.765	0.07514	2.323	0.981	0.312	1.237
SN00113059	52	457.6	99.5	-5.552	622.8	5.373	8	2	2	7.117	0.28495	2.899	0.966	0.495	1.313
SN00113247	53	408.4	119.3	-5.328	530.6	5.712	8	1	2	-7.121	0.16622	2.979	1.147	0.734	0.621
SN00113249	53	408.4	119.3	-5.328	530.6	5.712	8	1	2	-7.121	0.16622	2.979	1.147	0.734	0.621
**SN00113250**	**53**	**408.4**	**119.3**	**-5.328**	**530.6**	**5.712**	**8**	**1**	**2**	**-7.121**	**0.16622**	**2.979**	**1.147**	**0.734**	**0.621**
SN00113539	53	422.1	119.3	-5.838	556.6	6.357	8	1	2	-6.227	0.13126	2.855	1.083	0.654	0.694
SN00113541	53	422.1	119.3	-5.838	556.6	6.357	8	1	2	-6.227	0.13126	2.855	1.083	0.654	0.694
SN00113542	53	422.1	119.3	-5.838	556.6	6.357	8	1	2	-6.227	0.13126	2.855	1.083	0.654	0.694
SN00113629	53	399.0	110.2	-6.999	541.6	5.929	8	1	2	-6.172	0.12393	2.952	1.148	0.710	0.616
SN00113631	53	399.0	110.2	-6.999	541.6	5.929	8	1	2	-6.172	0.12393	2.952	1.148	0.710	0.616
SN00113632	53	399.0	110.2	-6.999	541.6	5.929	8	1	2	-6.172	0.12393	2.952	1.148	0.710	0.616
SN00115843	54	465.9	91.9	-5.653	621.7	5.451	9	0	2	7.268	0.27690	2.625	0.682	0.517	0.891
SN00119195	55	310.6	54.5	-6.167	443.5	4.259	4	0	0	2.541	0.08980	2.680	1.774	0.670	0.620
SN00119196	55	310.6	54.5	-6.167	443.5	4.259	4	0	0	2.541	0.08980	2.680	1.774	0.670	0.620
SN00121862	55	292.9	37.4	-6.245	415.5	4.857	3	0	0	3.062	0.08600	2.573	1.819	0.666	0.669
SN00121863	55	292.9	37.4	-6.245	415.5	4.857	3	0	0	3.062	0.08600	2.573	1.819	0.666	0.669
SN00121871	55	306.6	37.4	-6.515	429.5	5.312	3	0	1	2.614	0.07443	2.538	1.689	0.773	0.601
SN00141055	55	326.2	37.4	-7.356	500.4	5.591	3	0	2	-2.405	0.11959	2.213	1.674	1.096	0.541

SN00123510	56	348.9	77.3	-6.368	462.5	5.223	6	0	1	-1.770	0.20038	2.331	1.493	0.719	1.727
SN00128374	57	306.7	97.2	-7.291	442.4	4.691	6	1	0	-0.534	0.05304	2.754	1.225	0.809	0.662
SN00128422	58	325.3	45.2	-8.485	458.0	6.384	4	1	1	4.426	0.14961	2.842	0.846	0.718	1.199
SN00128855	59	412.6	112.9	-7.249	564.0	5.145	7	2	2	2.941	0.09264	2.610	0.743	0.823	0.724
SN00129492	60	447.7	123.5	-6.900	596.6	5.410	10	2	2	4.973	0.24397	2.069	0.778	0.676	0.951
SN00131673	61	265.1	70.1	-8.247	374.4	3.937	4	0	0	-0.838	0.07709	2.363	1.453	0.523	1.022
SN00133706	62	384.5	47.1	-5.351	514.6	4.131	6	0	1	4.249	0.15674	2.189	1.579	0.494	1.063
SN00139770	62	419.1	47.1	-6.957	564.7	5.325	6	0	2	4.249	0.05624	2.189	1.579	0.494	1.063
ZINC20563426	62	314.2	67.6	-5.227	429.5	3.398	7	1	0	3.814	0.55587	2.465	1.112	0.488	1.330
ZINC20563632	62	317.4	67.6	-4.711	441.5	2.978	7	1	0	0.579	0.50642	2.564	0.733	0.429	1.273
**SN00150533**	**63**	**352.8**	**40.5**	**-6.461**	**457.5**	**6.196**	**4**	**0**	**1**	**2.201**	**0.28254**	**2.664**	**1.292**	**0.746**	**0.556**
SN00154784	64	334.1	92.4	-3.671	446.5	3.255	6	2	0	1.668	0.63141	3.007	1.058	0.535	0.726
SN00166919	65	427.6	66.5	-6.340	592.7	5.386	8	0	2	1.987	0.19885	2.686	1.287	0.700	1.224
SN00245953	65	411.7	77.5	-6.026	578.7	5.110	8	1	2	1.983	0.22382	2.643	1.180	0.699	1.276
SN00261583	65	382.8	83.4	-8.243	534.6	6.481	7	3	2	4.848	0.17121	2.452	0.731	0.392	1.772
SN00275452	65	424.0	77.5	-5.833	592.7	5.304	8	1	2	1.983	0.21756	2.643	1.180	0.699	1.276
SN00384061	65	383.7	97.6	-6.867	548.6	5.531	8	2	2	-1.773	0.12085	2.598	0.627	0.691	1.433
SN00169398	66	464.2	84.8	-7.148	620.0	6.391	5	5	2	4.395	0.19609	2.949	0.947	0.384	1.544
SN00169407	66	477.4	84.8	-7.436	644.0	6.434	5	5	2	3.161	0.18158	2.923	1.039	0.484	1.362
SN00169411	66	446.8	93.0	-6.792	606.9	6.399	5	5	2	4.019	0.20645	2.846	0.627	0.531	1.438
SN00169419	66	460.0	93.0	-7.080	630.9	6.441	5	5	2	2.785	0.18828	2.809	0.670	0.594	1.246
SN00169448	66	426.7	72.7	-6.818	576.9	6.290	4	4	2	4.019	0.22051	2.841	0.946	0.421	1.657
SN00169461	66	416.2	72.7	-6.904	574.9	5.997	4	4	2	1.528	0.21055	2.736	0.857	0.371	1.760
SN00347694	66	446.1	45.2	-7.213	669.0	6.388	5	1	2	-2.782	0.05839	3.031	0.879	0.385	0.983
SN00169939	67	418.4	43.1	-8.938	592.9	6.337	3	2	2	-7.573	0.06144	2.697	1.395	0.907	0.519
SN00169941	67	416.5	43.1	-8.867	590.9	6.500	3	2	2	-7.107	0.06046	2.697	1.395	0.907	0.519
SN00169953	67	390.3	29.1	-8.053	539.8	5.950	3	2	2	-6.866	0.07400	2.689	1.295	0.786	0.673
SN00229183	68	360.3	49.0	-5.774	486.7	5.049	4	3	1	1.338	0.33755	2.709	0.703	0.657	1.354
SN00237128	69	482.3	61.1	-4.978	690.9	4.561	8	4	1	5.164	0.32946	2.872	1.055	0.701	1.245
SN00288697	69	482.3	61.1	-4.978	690.9	4.561	8	4	1	5.164	0.32946	2.872	1.055	0.701	1.245

SN00293304	69	482.3	61.1	-4.978	690.9	4.561	8	4	1	5.164	0.26357	2.926	1.013	0.660	1.385
SN00323840	69	482.3	61.1	-4.978	690.9	4.561	8	4	1	5.164	0.32946	2.872	1.055	0.701	1.245
SN00276332	70	429.7	77.5	-6.719	570.7	6.479	8	0	2	2.512	0.12816	2.486	1.108	0.440	0.929
SN00312143	70	429.7	77.5	-6.874	570.7	6.368	8	0	2	2.512	0.12790	2.454	1.013	0.423	0.978
SN00387973	70	403.2	77.5	-6.407	544.6	6.070	8	0	2	2.512	0.14849	2.491	1.000	0.468	0.952
SN00325070	71	377.2	161.3	-3.702	568.5	1.579	10	2	1	-0.035	0.25653	2.889	0.824	1.164	0.751
SN00338156	72	334.7	152.0	-5.072	546.5	2.960	10	4	1	1.574	0.09404	3.052	0.491	0.918	1.056
SN00352431	72	334.7	152.0	-5.072	546.5	2.960	10	4	1	1.574	0.09404	3.052	0.491	0.918	1.056
SN00380580	73	442.0	69.7	-5.734	606.7	5.307	8	0	2	5.407	0.13743	3.039	1.623	0.707	0.530
ZINC10012991	74	322.2	34.1	-8.131	442.5	5.612	4	0	1	-2.835	0.15476	2.479	1.129	0.563	1.134
ZINC02092906	75	291.6	56.5	-6.296	403.5	5.846	5	1	1	-5.535	0.10473	2.442	0.757	0.400	0.680
ZINC02093144	75	279.8	56.5	-6.236	411.4	5.360	5	1	1	-6.875	0.11313	2.538	0.706	0.548	0.606
ZINC02094748	75	291.6	56.5	-6.296	403.5	5.846	5	1	1	-5.535	0.10473	2.442	0.757	0.400	0.680
ZINC02148960	75	293.1	56.5	-6.111	403.5	5.918	5	1	1	-5.699	0.10627	2.452	0.832	0.438	0.703
ZINC04744033	75	285.7	56.5	-5.812	407.4	4.858	5	1	0	-6.841	0.13206	2.683	0.823	0.573	0.612
ZINC04744105	75	273.5	56.5	-5.468	393.4	4.514	5	1	0	-6.841	0.15005	2.683	0.823	0.573	0.612
ZINC05235687	75	301.0	56.5	-5.850	421.5	5.399	5	1	1	-6.875	0.11757	2.607	0.745	0.585	0.818
ZINC05235703	75	291.6	56.5	-5.842	403.5	5.101	5	1	1	-5.501	0.12723	2.534	0.797	0.348	0.674
ZINC06624400	75	271.9	73.6	-5.771	418.4	4.135	7	2	0	-5.501	0.14581	2.359	0.239	0.363	0.896
ZINC06624464	75	246.6	56.5	-6.276	401.4	4.075	7	2	0	-5.501	0.13897	2.639	0.028	0.514	1.137
ZINC08490711	75	259.6	73.6	-5.427	404.4	3.791	7	2	0	-5.501	0.16352	2.359	0.239	0.363	0.896
ZINC08764854	75	263.5	56.5	-5.863	400.4	2.765	6	2	0	-5.388	0.16434	2.491	0.558	0.465	0.780
ZINC09059583	75	258.9	56.5	-6.620	415.4	4.419	7	2	0	-5.501	0.12486	2.639	0.028	0.514	1.137
ZINC09481951	75	286.7	69.4	-6.044	412.4	5.020	6	1	1	-5.275	0.12364	2.380	0.839	0.451	0.691
ZINC11867425	75	314.6	56.5	-6.896	437.5	5.729	5	1	1	-3.023	0.09875	2.560	0.709	0.311	0.696
ZINC11867433	75	294.8	56.5	-6.234	423.9	5.363	5	1	1	-5.360	0.11158	2.650	0.865	0.700	0.584
ZINC12892460	75	298.3	84.8	-7.083	444.5	4.121	6	1	0	-2.487	0.12711	2.515	0.553	0.664	1.060
ZINC12899220	75	285.7	73.6	-6.041	432.5	4.589	7	2	0	-5.535	0.12863	2.501	0.299	0.343	1.088
ZINC13691831	75	296.8	84.8	-7.157	444.5	4.011	6	1	0	-2.447	0.12829	2.492	0.635	0.746	0.890
ZINC04085602	75	290.0	56.5	-5.354	425.5	4.228	6	2	0	-5.050	0.15346	3.008	0.262	0.602	1.078

ZINC12882862	75	302.2	56.5	-5.698	439.6	4.572	6	2	0	-5.050	0.13511	3.008	0.262	0.602	1.078
ZINC02093614	75	319.4	56.5	-6.184	431.5	6.111	5	1	1	-6.278	0.09804	2.623	0.770	0.583	0.669
ZINC30819216	75	291.9	56.5	-5.495	403.5	5.154	5	1	1	-5.535	0.13359	2.523	0.700	0.327	0.795
ZINC30819218	75	291.9	56.5	-5.495	403.5	5.154	5	1	1	-5.535	0.13359	2.523	0.700	0.327	0.795
ZINC30819226	75	288.9	56.5	-5.945	395.5	5.347	5	1	1	-9.883	0.12068	2.710	0.639	0.522	0.695
ZINC30819257	75	301.2	56.5	-6.289	409.5	5.691	5	1	1	-9.883	0.10630	2.710	0.639	0.522	0.695
**ZINC09531209**	**75**	**313.3**	**74.3**	**-5.966**	**441.5**	**4.622**	**7**	**1**	**0**	**-1.621**	**0.14820**	**2.320**	**0.915**	**0.544**	**1.059**
ZINC11936239	76	335.0	50.8	-5.599	435.5	3.036	5	2	0	1.803	0.49740	2.629	0.692	0.251	1.429
ZINC12182014	77	291.1	77.4	-4.761	418.5	1.516	8	1	0	3.053	0.64827	2.216	1.034	0.348	1.474
ZINC12188773	78	313.3	68.9	-5.102	429.5	2.206	7	1	0	0.849	0.51730	2.768	0.646	0.486	1.420
ZINC01223288	79	279.8	81.3	-6.730	404.5	2.277	6	1	0	-1.202	0.30182	2.525	1.266	0.588	1.311
ZINC01223299	79	290.8	81.3	-7.108	418.5	2.602	6	1	0	-1.080	0.28736	2.526	1.429	0.648	1.159
ZINC01223300	79	290.8	81.3	-7.108	418.5	2.602	6	1	0	-1.080	0.28736	2.526	1.429	0.648	1.159
ZINC12440825	80	345.9	65.6	-4.229	450.5	3.061	7	0	0	-0.800	0.25491	2.621	1.269	0.576	1.282
ZINC12583319	81	330.6	70.4	-7.971	428.6	6.401	4	1	1	-0.474	0.11093	2.454	0.697	0.793	1.304
ZINC12682031	82	311.2	56.5	-6.105	414.5	4.234	6	0	0	2.588	0.44008	2.758	1.160	0.408	1.426
ZINC13120611	83	329.5	57.0	-5.991	440.5	5.690	6	1	1	-1.666	0.20523	2.562	1.536	0.492	1.140
ZINC13226339	84	312.9	78.0	-5.005	447.5	2.637	8	2	0	3.333	0.58229	2.541	0.866	0.428	1.353
ZINC08765217	85	277.5	72.3	-4.348	406.5	1.410	7	2	0	-4.206	0.21833	2.687	0.878	0.406	1.279
ZINC08765269	85	305.3	88.1	-4.618	420.5	3.842	7	2	0	-3.241	0.18812	2.815	0.760	0.416	1.315
**ZINC13623590**	**85**	**322.2**	**56.5**	**-5.050**	**444.5**	**3.000**	**6**	**0**	**0**	**-3.061**	**0.18200**	**2.747**	**1.201**	**0.603**	**1.018**
ZINC30819219	85	301.6	56.5	-5.411	415.5	5.230	5	0	1	-5.535	0.13172	2.546	0.912	0.401	0.919
ZINC02098205	85	311.6	102.6	-4.892	424.5	0.239	7	0	0	-7.577	0.19536	2.404	0.881	0.442	0.671
ZINC02107387	85	312.7	102.6	-5.436	438.5	2.190	7	1	0	-5.870	0.17063	2.568	0.888	0.372	0.747
ZINC01405393	86	311.6	94.2	-7.564	438.5	4.891	5	1	0	-3.837	0.06303	2.602	0.832	0.631	1.251
ZINC15468981	87	294.6	38.9	-6.121	396.5	0.589	5	1	0	0.501	0.44605	2.564	1.182	0.770	1.099
ZINC15733799	88	301.0	51.2	-5.023	437.6	3.303	5	1	0	-3.434	0.29836	2.539	0.243	0.461	1.469
ZINC20990084	88	294.6	51.2	-5.028	437.6	3.595	5	1	0	-0.574	0.38422	2.595	0.680	0.499	1.345
ZINC16269305	89	322.3	57.0	-6.467	436.5	5.284	6	1	1	2.165	0.09747	2.335	1.294	0.519	1.087
ZINC16284359	89	334.8	57.0	-6.650	450.5	5.784	6	1	1	1.903	0.08442	2.371	1.320	0.547	1.011

ZINC16349822	89	310.0	57.0	-6.123	422.5	4.941	6	1	0	2.165	0.11062	2.319	1.230	0.465	1.115
ZINC16362158	89	322.3	57.0	-6.467	436.5	5.284	6	1	1	2.165	0.07310	2.410	1.248	0.514	1.127
ZINC16363024	89	310.0	57.0	-6.123	422.5	4.941	6	1	0	2.165	0.08297	2.398	1.183	0.462	1.151
ZINC08335520	90	298.5	47.8	-6.687	406.5	5.710	5	1	1	2.213	0.09231	2.417	1.567	0.694	1.307
ZINC08367670	90	289.4	47.8	-6.735	412.9	5.628	5	1	1	2.274	0.09257	2.399	1.535	0.952	1.120
ZINC08367675	90	296.3	57.0	-6.017	408.5	4.952	6	1	0	2.240	0.11476	2.431	1.718	0.482	1.163
ZINC16284487	90	304.8	47.8	-7.471	447.3	6.234	5	1	1	2.251	0.07486	2.399	1.535	0.952	1.120
ZINC16349120	90	286.3	47.8	-6.343	392.5	5.366	5	1	1	2.213	0.10390	2.413	1.521	0.625	1.372
ZINC22530327	90	334.8	57.0	-6.747	450.5	6.031	6	1	1	1.768	0.07959	2.420	1.656	0.705	0.898
ZINC00980500	90	302.0	79.9	-6.544	437.5	5.204	6	2	1	2.575	0.09937	2.405	1.125	0.599	1.510
ZINC08440247	91	318.1	84.6	-6.948	421.5	4.324	6	1	0	3.655	0.11433	2.198	1.319	0.584	0.884
ZINC16671995	91	321.3	84.6	-7.340	441.9	4.586	6	1	0	3.697	0.10337	2.153	1.368	0.844	0.865
ZINC17093033	92	305.8	47.8	-5.477	422.5	5.110	5	1	1	1.337	0.31333	2.511	1.327	0.642	1.073
ZINC19660588	93	296.0	67.6	-5.578	419.5	3.213	7	1	0	1.874	0.50779	2.578	1.008	0.649	1.358
ZINC23333375	94	273.5	43.1	-6.719	404.4	4.926	4	1	0	-5.096	0.19351	2.523	1.387	0.726	1.050
ZINC27534122	95	293.2	71.5	-6.887	439.5	3.197	7	1	0	-0.631	0.29648	2.400	0.458	0.427	1.136
ZINC03358607	96	340.0	72.8	-6.743	449.6	5.733	5	0	1	-0.998	0.19883	2.551	0.016	0.523	1.356
ZINC04262734	97	310.5	77.3	-4.938	414.5	4.434	6	0	0	2.097	0.30555	2.480	0.876	0.887	0.682
ZINC04936227	98	317.5	34.9	-5.960	426.6	2.529	5	1	0	2.300	0.49984	2.608	0.991	0.573	1.302
ZINC05151985	98	331.2	47.8	-6.001	426.6	4.650	5	1	0	2.300	0.40995	2.640	0.870	0.642	1.294
ZINC09408007	98	331.3	34.9	-6.369	440.6	3.100	5	1	0	2.554	0.45284	2.665	0.898	0.669	1.224
ZINC09408500	98	331.0	34.9	-6.577	440.6	2.930	5	1	0	2.300	0.26450	2.640	0.870	0.642	1.294
ZINC09408505	98	343.5	47.8	-6.345	440.6	4.994	5	1	0	2.300	0.21672	2.640	0.870	0.642	1.294
ZINC05044064	99	320.8	73.2	-5.984	422.6	5.999	5	1	1	-1.102	0.17314	2.567	1.392	0.730	1.080
ZINC08857160	100	315.7	71.6	-7.840	449.3	6.391	6	0	1	3.181	0.25543	2.381	1.485	0.840	1.279
ZINC08966188	101	286.5	60.4	-5.624	443.5	3.778	7	2	0	-4.305	0.25185	2.498	0.860	0.538	1.212
ZINC09076927	101	290.9	87.0	-4.434	428.5	2.377	9	1	0	0.389	0.53742	2.625	1.318	0.511	1.300
ZINC09792243	101	293.2	60.4	-5.397	443.5	3.692	7	1	0	-2.425	0.28166	2.683	1.020	0.527	1.224
ZINC09792247	101	293.2	60.4	-5.397	443.5	3.692	7	1	0	-2.425	0.28166	2.683	1.020	0.527	1.224
ZINC09992380	101	300.0	60.4	-5.053	449.5	3.638	7	1	0	-5.508	0.27596	2.705	0.675	0.509	1.354

ZINC09992418	101	300.0	60.4	-5.053	449.5	3.638	7	1	0	-5.508	0.27596	2.705	0.675	0.509	1.354
ZINC09043059	102	286.1	85.4	-6.152	447.4	0.536	5	0	0	-1.527	0.30017	2.436	1.394	0.907	0.908
**ZINC09530812**	**103**	**265.2**	**30.3**	**-5.035**	**419.9**	**1.801**	**6**	**1**	**0**	**2.293**	**0.61268**	**2.811**	**0.592**	**0.724**	**1.257**
ZINC09580205	104	343.1	49.2	-6.000	445.5	5.627	5	0	1	1.033	0.18478	2.220	1.288	0.400	1.427
ZINC09682151	105	313.0	54.7	-5.395	436.5	4.443	6	1	0	3.625	0.47600	2.459	1.109	0.436	1.290
ZINC09940952	106	332.7	59.2	-7.283	441.9	5.003	5	0	1	4.098	0.33862	2.390	1.562	0.856	1.250
ZINC09940957	106	341.8	59.2	-7.235	435.5	5.085	5	0	1	4.050	0.33850	2.547	1.651	0.556	1.268
ZINC09941299	106	337.4	59.2	-7.020	439.5	4.914	5	0	0	2.535	0.34261	2.565	1.496	0.752	1.309

Abbreviations: Topological polar surface area (TPSA); molecular weight (MW); the calculated logarithm (base 10) of the solubility measured in mol/liter (cLogS); calculated logarithm of partition coefficient between n-octanol and water (cLogP); number of hydrogen bond donors (HBD); number of hydrogen bond acceptors (HBA); violation of Lipinski's rules (Ro5 violations), FT, Fish Toxicity; TPT, *Tetrahymena Pyriformis* Toxicity; RAT, Rat Acute Toxicity. LD50 is the amount of a compound, given all at once, which causes the death of 50% (one half) of a group of test rats.

[a] These parameters were calculated using the DATAWARRIOR software v4.7.2 [4].

[b] These parameters were calculated using the http://lmmd.ecust.edu.cn:8000/predict/ site [5].

Supplementary Figure S1. Detailed description of the molecular interactions between the inhibitor compound and the rapamycin binding site in mTOR for **Figure 5**. Structure visualization was by PyMol 2.0 software. The interactions have been detected with the FLIP algorithm [6].

Hydrophobic Interactions ····

Index	Residue	AA	Distance	Ligand Atom	Protein Atom
1	77A	PHE	3.55	1826	453
2	77A	PHE	3.68	1823	451
3	86A	VAL	3.60	1827	517
4	87A	ILE	3.69	1828	524
5	90A	TRP	3.65	1827	548
6	2031B	LEU	3.61	1807	1084
7	2032B	GLU	3.96	1806	1098
8	2039B	PHE	3.40	1816	1167
9	2101B	TRP	3.65	1814	1684
10	2101B	TRP	3.47	1808	1692
11	2105B	TYR	3.83	1813	1726
12	2108B	PHE	3.78	1807	1755

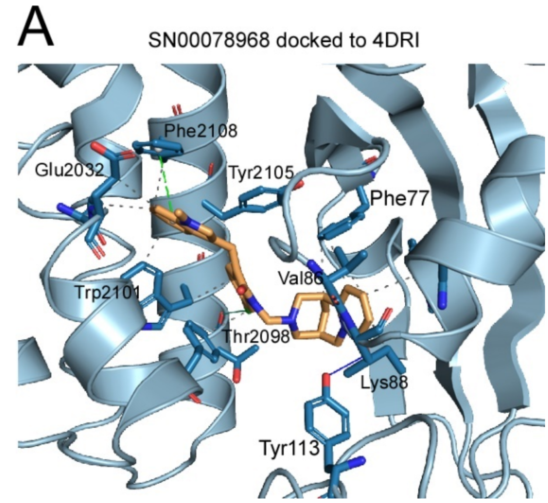

Hydrogen Bonds —

Index	Residue	AA	Distance H-A	Distance D-A	Donor Angle	Donor Atom	Acceptor Atom
1	113A	TYR	1.97	2.79	145.38	740 [O3]	1800 [O2]

π-Stacking ···· ····

Index	Residue	AA	Distance	Angle	Offset	Type	Ligand Atoms
1	2108B	PHE	4.30	27.57	1.40	P	1803, 1805, 1806, 1807, 1808, 1809

Halogen Bonds —

Index	Residue	AA	Distance	Donor Angle	Acceptor Angle	Donor Atom	Acceptor Atom
1	2098B	THR	3.95	137.39	121.33	1831 [Cl]	1662 [O2]

Hydrophobic Interactions ····

Index	Residue	AA	Distance	Ligand Atom	Protein Atom
1	57A	TYR	3.99	1798	289
2	57A	TYR	3.87	1795	290
3	77A	PHE	3.46	1795	453
4	90A	TRP	3.59	1798	552
5	121A	LYS	3.94	1827	785

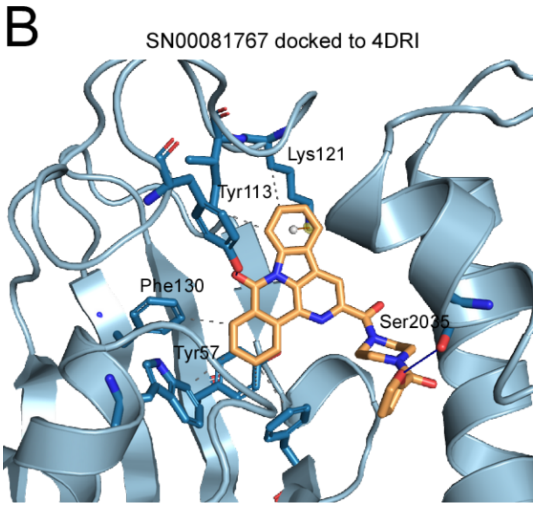

Index	Residue	AA	Distance	Ligand Atom	Protein Atom
6	122A	ILE	3.41	1828	797
7	130A	PHE	3.53	1801	866

Hydrogen Bonds ━━

Index	Residue	AA	Distance H-A	Distance D-A	Donor Angle	Donor Atom	Acceptor Atom
1	113A	TYR	2.68	3.52	151.05	740 [O3]	1806 [O2]
2	2035B	SER	2.45	3.22	136.39	1125 [O3]	1803 [O2]

π-Cation Interactions ····

Index	Residue	AA	Distance	Offset	Ligand Group	Ligand Atoms
1	121A	LYS	5.34	1.57	Aromatic	1824, 1825, 1826, 1827, 1828, 1829

Hydrophobic Interactions ····

Index	Residue	AA	Distance	Ligand Atom	Protein Atom
1	67A	PHE	3.86	1818	365
2	68A	ASP	3.66	1818	374
3	77A	PHE	3.59	1807	453
4	121A	LYS	3.45	1817	787
5	2031B	LEU	3.35	1827	1084
6	2101B	TRP	3.48	1795	1684
7	2101B	TRP	3.60	1828	1692
8	2105B	TYR	3.57	1804	1728
9	2108B	PHE	3.80	1830	1760
10	2108B	PHE	3.53	1828	1755

C

SN00082651 docked to 4DRI

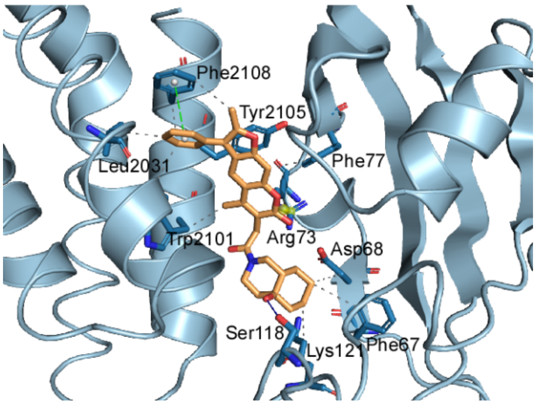

Hydrogen Bonds ━━

Index	Residue	AA	Distance H-A	Distance D-A	Donor Angle	Donor Atom	Acceptor Atom
1	118A	SER	2.56	2.93	102.21	765 [O3]	1803 [O3]

π-Stacking ···· ····

Index	Residue	AA	Distance	Angle	Offset	Type	Ligand Atoms
1	2108B	PHE	4.08	11.11	1.61	P	1824, 1825, 1826, 1827, 1828, 1829

Salt Bridges ····

Index	Residue	AA	Distance	Ligand Group	Ligand Atoms
1	73A	ARG	4.75	Carboxylate	1797, 1799

Hydrophobic Interactions ····

Index	Residue	AA	Distance	Ligand Atom	Protein Atom
1	77A	PHE	4.00	1829	453
2	86A	VAL	3.79	1825	516
3	87A	ILE	3.74	1826	524
4	90A	TRP	3.63	1827	552
5	130A	PHE	3.69	1827	866
6	2039B	PHE	3.79	1801	1161
7	2039B	PHE	3.63	1818	1167
8	2101B	TRP	3.64	1812	1689
9	2105B	TYR	3.62	1812	1726
10	2108B	PHE	3.44	1814	1758

D

SN00097418 docked to 4DRI

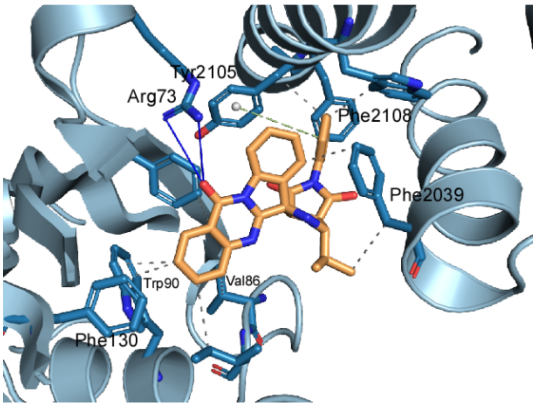

Hydrogen Bonds —

Index	Residue	AA	Distance H-A	Distance D-A	Donor Angle	Donor Atom	Acceptor Atom
1	73A	ARG	3.53	4.05	115.04	417 [Ng+]	1803 [O2]
2	73A	ARG	3.30	3.82	114.67	418 [Ng+]	1803 [O2]

π-Stacking ···· ····

Index	Residue	AA	Distance	Angle	Offset	Type	Ligand Atoms
1	2105B	TYR	5.16	66.02	1.24	T	1810, 1811, 1812, 1813, 1814, 1815

Hydrophobic Interactions ····

Index	Residue	AA	Distance (Å)	Ligand Atom	Protein Atom
1	77A	PHE	3.99	1814	453
2	85A	GLN	3.60	1831	508
3	86A	VAL	3.98	1816	517
4	113A	TYR	3.88	1823	737
5	2031B	LEU	3.89	1795	1084
6	2032B	GLU	3.70	1832	1098
7	2039B	PHE	3.42	1824	1161
8	2101B	TRP	3.35	1801	1689
9	2101B	TRP	3.63	1795	1692
10	2105B	TYR	3.63	1826	1730
11	2105B	TYR	3.97	1807	1726
12	2108B	PHE	3.72	1832	1760

E

SN00111586 docked to 4DRI

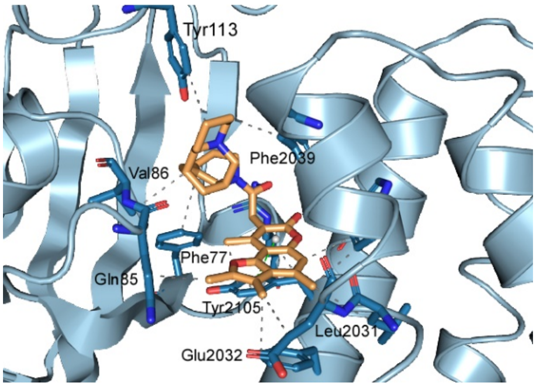

Hydrogen Bonds

Index	Residue	AA	Distance H-A	Distance D-A	Donor Angle	Donor Atom	Acceptor Atom
1	73A	ARG	2.78	3.19	105.66	418 [N+]	1802 [O2]

π-Stacking

Index	Residue	AA	Distance	Angle	Offset	Type	Ligand Atoms
1	2105B	TYR	4.05	16.78	1.55	P	1800, 1803, 1805, 1806, 1807, 1808

Hydrophobic Interactions

Index	Residue	AA	Distance (Å)	Ligand Atom	Protein Atom
1	77A	PHE	3.15	1795	453
2	86A	VAL	3.67	1795	517
3	2039B	PHE	3.53	1812	1167
4	2101B	TRP	3.75	1822	1692
5	2105B	TYR	3.41	1827	1730
6	2105B	TYR	3.97	1815	1726
7	2108B	PHE	3.47	1830	1760

F

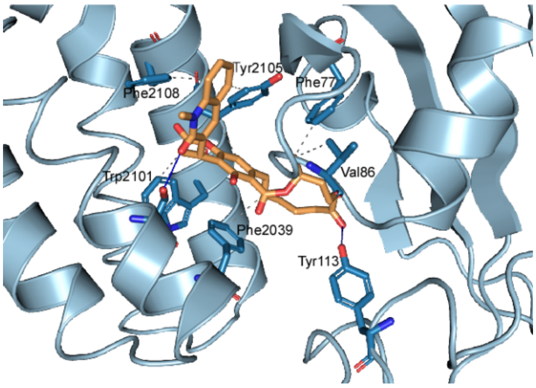

Hydrogen Bonds

Index	Residue	AA	Distance H-A	Distance D-A	Donor Angle	Donor Atom	Acceptor Atom
1	113A	TYR	3.23	3.87	126.64	740 [O3]	1797 [O2]
2	2035B	SER	2.40	3.18	136.25	1125 [O3]	1809 [O2]

Hydrophobic Interactions

Index	Residue	AA	Distance	Ligand Atom	Protein Atom
1	57A	TYR	4.00	1803	289
2	67A	PHE	3.59	1810	367
3	77A	PHE	3.76	1804	452
4	77A	PHE	3.60	1818	451
5	86A	VAL	3.74	1795	517
6	87A	ILE	3.71	1798	524
7	122A	ILE	3.59	1811	797
8	130A	PHE	3.59	1800	868
9	130A	PHE	3.92	1802	866
10	2031B	LEU	3.39	1827	1084
11	2101B	TRP	3.64	1826	1692

G

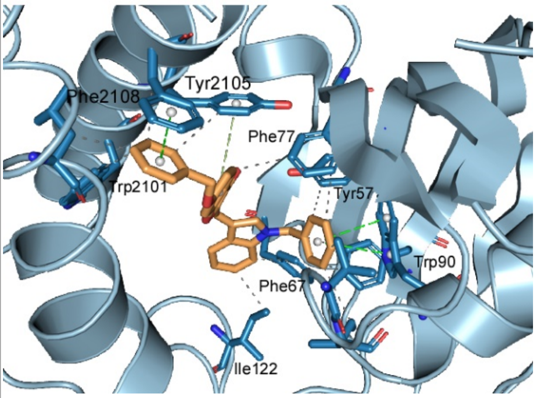

Index	Residue	AA	Distance	Ligand Atom	Protein Atom
12	2105B	TYR	3.79	1825	1728
13	2108B	PHE	3.68	1826	1755

π-Stacking

Index	Residue	AA	Distance	Angle	Offset	Type	Ligand Atoms
1	90A	TRP	4.31	21.46	1.64	P	1795, 1798, 1800, 1802, 1803, 1804
2	90A	TRP	4.25	23.09	1.45	P	1795, 1798, 1800, 1802, 1803, 1804
3	2105B	TYR	4.91	63.40	1.72	T	1817, 1818, 1819, 1820, 1821, 1822
4	2108B	PHE	4.02	11.38	1.46	P	1824, 1825, 1826, 1827, 1828, 1829

Hydrophobic Interactions

Index	Residue	AA	Distance	Ligand Atom	Protein Atom
1	77A	PHE	3.61	1795	453
2	86A	VAL	3.77	1795	517
3	2032B	GLU	3.30	1818	1098
4	2039B	PHE	3.65	1810	1162
5	2039B	PHE	3.48	1805	1167
6	2105B	TYR	3.54	1808	1729

H

ZINC8918463 docked to 4DRI

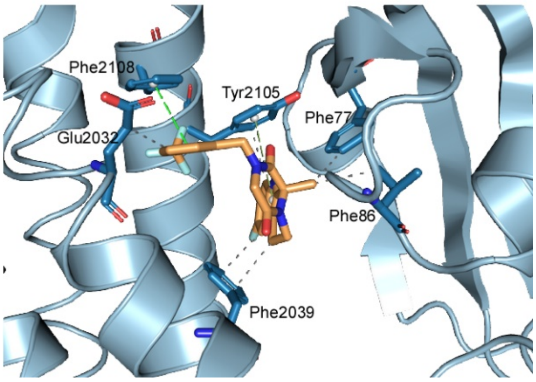

π-Stacking

Index	Residue	AA	Distance	Angle	Offset	Type	Ligand Atoms
1	2105B	TYR	5.10	63.07	1.03	T	1796, 1801, 1803, 1804, 1809
2	2108B	PHE	3.87	2.44	1.32	P	1816, 1817, 1818, 1819, 1820, 1821

Hydrophobic Interactions

Index	Residue	AA	Distance	Ligand Atom	Protein Atom
1	77A	PHE	3.77	1802	451
2	121A	LYS	3.89	1822	786
3	2032B	GLU	3.30	1810	1098

I

ZINC9530812 docked to 4DRI

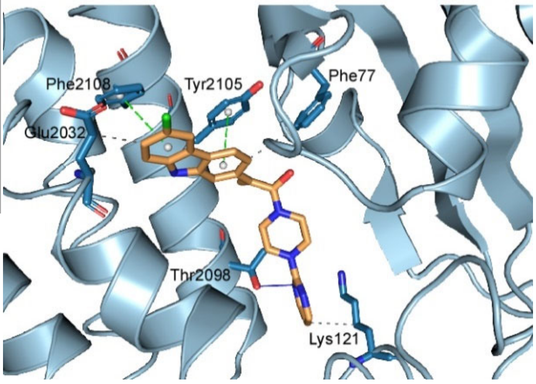

Hydrogen Bonds —

Index	Residue	AA	Distance H-A	Distance D-A	Donor Angle	Donor Atom	Acceptor Atom
1	2098B	THR	2.56	3.10	115.05	1665 [O3]	1805 [N2]

π-Stacking ···· ····

Index	Residue	AA	Distance	Angle	Offset	Type	Ligand Atoms
1	2105B	TYR	4.10	17.49	1.13	P	1800, 1802, 1804, 1806, 1813, 1814
2	2108B	PHE	4.31	24.22	1.82	P	1807, 1808, 1809, 1810, 1811, 1812

Hydrophobic Interactions ····

Index	Residue	AA	Distance	Ligand Atom	Protein Atom
1	57A	TYR	3.67	1825	289
2	77A	PHE	3.60	1826	451
3	130A	PHE	3.57	1825	866
4	2031B	LEU	3.58	1808	1084
5	2032B	GLU	3.89	1807	1098
6	2039B	PHE	3.55	1813	1161
7	2039B	PHE	3.71	1827	1164
8	2101B	TRP	3.79	1809	1692
9	2105B	TYR	3.45	1810	1728
10	2108B	PHE	3.64	1809	1755

J

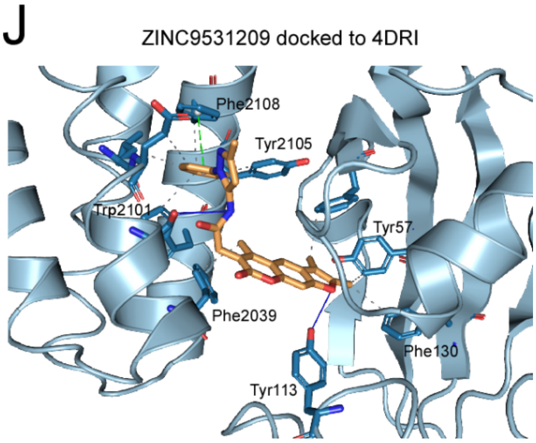

Hydrogen Bonds —

Index	Residue	AA	Distance H-A	Distance D-A	Donor Angle	Donor Atom	Acceptor Atom
1	113A	TYR	2.78	3.20	108.28	740 [O3]	1800 [O2]
2	2035B	SER	3.21	3.81	121.26	1125 [O3]	1802 [Nam]

π-Stacking ···· ····

Index	Residue	AA	Distance	Angle	Offset	Type	Ligand Atoms
1	2108B	PHE	3.96	3.76	1.45	P	1806, 1807, 1808, 1809, 1810, 1811

Hydrophobic Interactions ····

Index	Residue	AA	Distance	Ligand Atom	Protein Atom
1	77A	PHE	3.44	1807	453
2	2031B	LEU	3.47	1824	1084
3	2032B	GLU	3.77	1823	1098
4	2039B	PHE	3.85	1812	1165

K

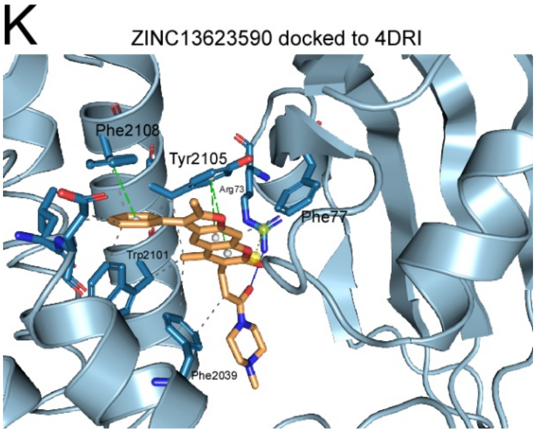

Index	Residue	AA	Distance	Ligand Atom	Protein Atom
5	2039B	PHE	3.95	1795	1167
6	2101B	TRP	3.37	1795	1684
7	2101B	TRP	3.77	1825	1692
8	2105B	TYR	3.87	1826	1728
9	2105B	TYR	3.97	1795	1726
10	2108B	PHE	3.57	1825	1755

Hydrogen Bonds

Index	Residue	AA	Distance H-A	Distance D-A	Donor Angle	Donor Atom	Acceptor Atom
1	73A	ARG	2.11	2.80	125.46	418 [Ng+]	1802 [O2]

π-Stacking

Index	Residue	AA	Distance	Angle	Offset	Type	Ligand Atoms
1	2105B	TYR	4.31	28.39	0.46	P	1801, 1803, 1805, 1806, 1807, 1808
2	2105B	TYR	4.88	28.72	0.92	P	1797, 1798, 1801, 1808, 1809, 1810
3	2108B	PHE	3.87	9.27	1.27	P	1821, 1822, 1823, 1824, 1825, 1826

Salt Bridges

Index	Residue	AA	Distance	Ligand Group	Ligand Atoms
1	73A	ARG	4.23	Carboxylate	1797, 1800

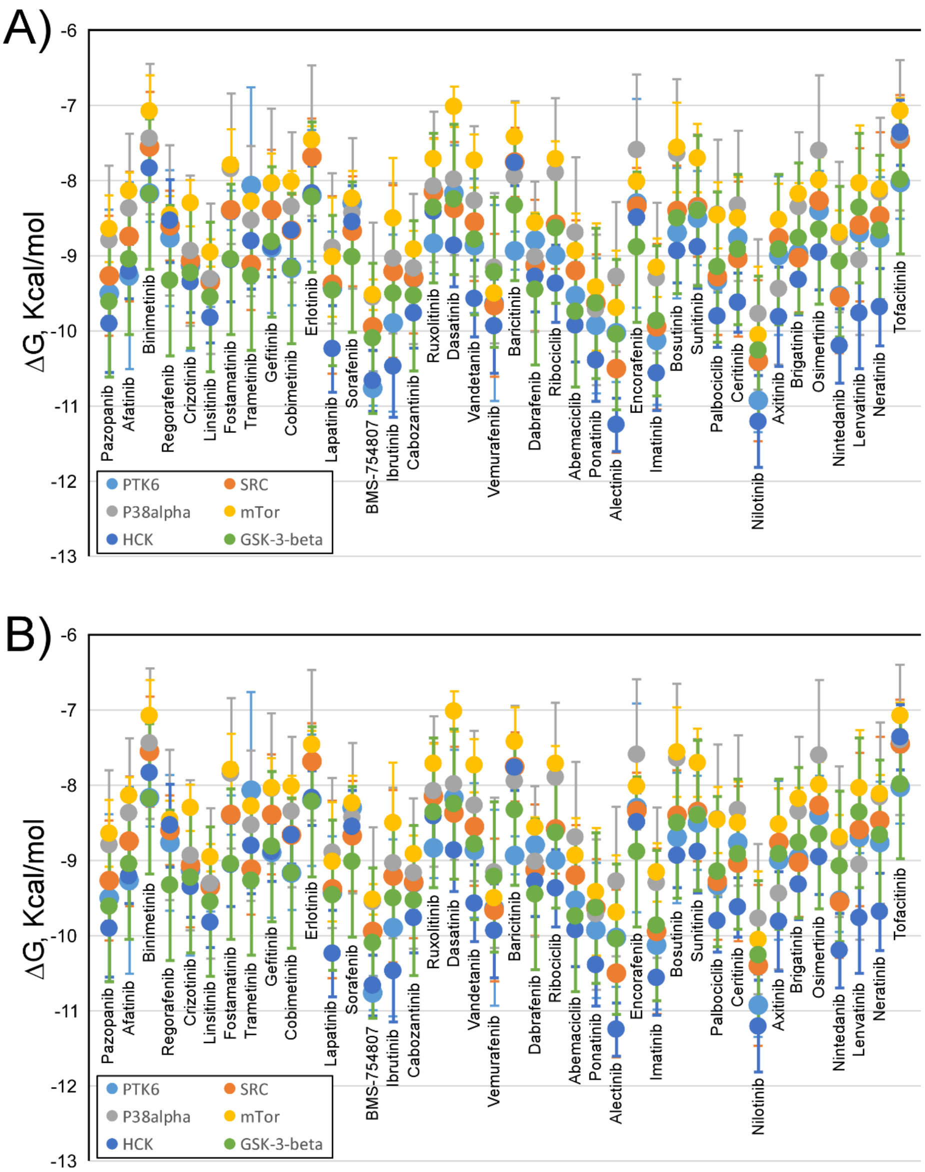

Supplementary Figure S2. Comparison of the Gibbs free energy variation (ΔG, Kcal/mol) for 40 compounds approved by the FDA for clinical use against various types of cancer, obtained by molecular docking experiments against the ATP binding site of 12 protein kinases (see below for both the UniProt and PDB code of each protein). The data has been distributed in two panels (**A** and **B**) to facilitate comparison.

List of PDB codes of the structures used in the molecular docking experiments for the different protein kinases analyzed. The results of ΔG included in **Supplementary Figure S2** result from averaging the values obtained in molecular docking experiments with all the structures listed in the next tables for each protein. For each PDB entry, the experimental method (X-ray diffraction data only), the resolution A, and the position of the amino acids of the resolved structure are included.

Protein: PTK6, **UniProt code**: Q13882

PDB entry	Method	Resolution (Å)	Positions
5D7V	X-ray	2.33	185-446
5DA3	X-ray	1.7	185-446
5H2U	X-ray	2.24	185-446

Protein: mTor, **UniProt code**: P42345

PDB entry	Method	Resolution (Å)	Positions
4JSN	X-ray	3.2	1376-2549
4JSP	X-ray	3.3	1376-2549
4JSV	X-ray	3.5	1376-2549
4JSX	X-ray	3.5	1376-2549
4JT5	X-ray	3.45	1376-2549
4JT6	X-ray	3.6	1376-2549
5WBU	X-ray	3.42	1376-2549
5WBY	X-ray	3.1	1376-2549

Protein: CHK2, **UniProt code**: O96017

PDB entry	Method	Resolution (Å)	Positions
2CN5	X-ray	2.25	210-531
2CN8	X-ray	2.7	210-531
2W0J	X-ray	2.05	210-531
2W7X	X-ray	2.07	210-531
2WTC	X-ray	3	210-531
2WTD	X-ray	2.75	210-531
2WTI	X-ray	2.5	210-531
2WTJ	X-ray	2.1	210-531
2XBJ	X-ray	2.3	210-531
2XK9	X-ray	2.35	210-531
2XM8	X-ray	3.4	210-531
2XM9	X-ray	2.5	210-531
2YCF	X-ray	1.77	210-530
2YCQ	X-ray	2.05	210-531
2YCR	X-ray	2.2	210-531
2YCS	X-ray	2.35	210-531
2YIQ	X-ray	1.89	210-531
2YIR	X-ray	2.1	210-531
2YIT	X-ray	2.2	210-531

Protein: SRC, **UniProt code**: P12931

PDB entry	Method	Resolution (Å)	Positions
2H8H	X-ray	2.2	2-536
1YOJ	X-ray	1.95	254-536
1YOL	X-ray	2.3	254-536
1YOM	X-ray	2.9	254-536
4MXO	X-ray	2.1	254-536
4MXX	X-ray	2.6	254-536
4MXY	X-ray	2.58	254-536
4MXZ	X-ray	2.58	254-536
2BDF	X-ray	2.1	258-536
2BDJ	X-ray	2.5	258-536
1YI6	X-ray	2	261-536
1FMK	X-ray	1.5	86-536
1KSW	X-ray	2.8	86-536
1Y57	X-ray	1.91	86-536
2SRC	X-ray	1.5	86-536
4K11	X-ray	2.3	87-534

Protein: HCK, **UniProt code**: P08631

PDB entry	Method	Resolution (Å)	Positions
5H0B	X-ray	1.65	81-526
5H0H	X-ray	1.72	81-526
5H0G	X-ray	1.8	81-526
5H09	X-ray	1.95	81-526
2HK5	X-ray	2	247-514
1QCF	X-ray	2	81-526
5H0E	X-ray	2.1	81-526
2C0T	X-ray	2.15	81-526
3VS3	X-ray	2.17	81-526
3VRZ	X-ray	2.22	81-526
2C0I	X-ray	2.3	81-526
3VS6	X-ray	2.37	81-526
3VS1	X-ray	2.46	81-526
3VRY	X-ray	2.48	81-526
1AD5	X-ray	2.6	79-526
3VS2	X-ray	2.61	81-526
3VS4	X-ray	2.75	81-526

3I6U	X-ray	3	84-502
3I6W	X-ray	3.25	70-512
4A9R	X-ray	2.85	210-531
4A9S	X-ray	2.66	210-531
4A9T	X-ray	2.7	210-531
4A9U	X-ray	2.48	210-531
4BDA	X-ray	2.6	210-531
4BDB	X-ray	2.5	210-531
4BDC	X-ray	3	210-531
4BDD	X-ray	2.67	210-531
4BDE	X-ray	2.55	210-531
4BDF	X-ray	2.7	210-531
4BDG	X-ray	2.84	210-531
4BDH	X-ray	2.7	210-531
4BDI	X-ray	2.32	210-531
4BDJ	X-ray	3.01	210-531
4BDK	X-ray	3.3	210-531

Protein: AKT2, **UniProt code**: P31751

PDB entry	Method	Resolution (Å)	Positions
1GZK	X-ray	2.3	146-460
1GZN	X-ray	2.5	146-480
1GZO	X-ray	2.75	146-460
1MRV	X-ray	2.8	143-481
1MRY	X-ray	2.8	143-481
1O6K	X-ray	1.7	146-481
1O6L	X-ray	1.6	146-467
2JDO	X-ray	1.8	146-467
2JDR	X-ray	2.3	146-467
2UW9	X-ray	2.1	146-467
2X39	X-ray	1.93	146-467
2XH5	X-ray	2.72	146-479
3D0E	X-ray	2	146-480
3E87	X-ray	2.3	146-480
3E88	X-ray	2.5	146-480
3E8D	X-ray	2.7	146-480

Protein: P38 alpha, **UniProt code**: Q16539

PDB entry	Method	Resolution (Å)	Positions
2FST	X-ray	1.45	2-360
3LFF	X-ray	1.5	2-360
3OEF	X-ray	1.6	1-360
5WJJ	X-ray	1.6	1-360
3ZS5	X-ray	1.6	2-360
4EHV	X-ray	1.6	2-360

2C0O	X-ray	2.85	81-526
3VS5	X-ray	2.85	81-526
4LUD	X-ray	2.85	81-526
3VS0	X-ray	2.93	81-526
2HCK	X-ray	3	79-526
3VS7	X-ray	3	81-526
4LUE	X-ray	3.04	81-526

Protein: FYN, **UniProt code**: P06241

PDB entry	Method	Resolution (Å)	Positions
2DQ7	X-ray	2.8	261-537

Protein: AKT1, **UniProt code**: P31749

PDB entry	Method	Resolution (Å)	Positions
6CCY	X-ray	2.18	144-466
3CQU	X-ray	2.2	144-480
3CQW	X-ray	2	144-480
3MV5	X-ray	2.47	144-480
3MVH	X-ray	2.01	144-480
3OCB	X-ray	2.7	144-480
3OW4	X-ray	2.6	144-480
3QKK	X-ray	2.3	144-480
3QKL	X-ray	1.9	144-480
3QKM	X-ray	2.2	144-480
4EKK	X-ray	2.8	144-480
4EKL	X-ray	2	144-480
4GV1	X-ray	1.49	144-480
3O96	X-ray	2.7	2-443
4EJN	X-ray	2.19	2-446
5KCV	X-ray	2.7	2-446

Protein: GSK-3 beta, **UniProt code**: P49841

PDB entry	Method	Resolution (Å)	Positions
1J1B	X-ray	1.8	1-420
1Q5K	X-ray	1.94	7-420
4AFJ	X-ray	1.98	27-393
4PTE	X-ray	2.03	1-420
4NM3	X-ray	2.1	1-383
1J1C	X-ray	2.1	1-420
1Q41	X-ray	2.1	2-420
3DU8	X-ray	2.2	1-420
1Q3D	X-ray	2.2	2-420
5K5N	X-ray	2.2	28-384
4ACC	X-ray	2.21	1-420
3SAY	X-ray	2.23	1-420

4GEO	X-ray	1.66	2-360
3FMK	X-ray	1.7	1-360
3ROC	X-ray	1.7	1-360
5XYY	X-ray	1.7	1-360
2FSL	X-ray	1.7	2-360
2QD9	X-ray	1.7	2-360
3K3I	X-ray	1.7	5-352
2RG6	X-ray	1.72	2-360
2GFS	X-ray	1.75	2-360
2ZAZ	X-ray	1.8	1-360
3FL4	X-ray	1.8	1-360
3FLY	X-ray	1.8	1-360
3GC7	X-ray	1.8	1-360
3KQ7	X-ray	1.8	1-360
5MTX	X-ray	1.8	1-360
1WBS	X-ray	1.8	2-360
2NPQ	X-ray	1.8	2-360
3HUC	X-ray	1.8	2-360
4AA0	X-ray	1.8	2-360
3S3I	X-ray	1.8	4-352
2FSO	X-ray	1.83	2-360
5N68	X-ray	1.85	1-360
4F9Y	X-ray	1.85	2-360
2FSM	X-ray	1.86	2-360
4E5A	X-ray	1.87	1-360
3HL7	X-ray	1.88	1-360
3NNW	X-ray	1.89	1-354
3MPT	X-ray	1.89	1-360
3ZSG	X-ray	1.89	2-360
3D83	X-ray	1.9	1-360
3FLN	X-ray	1.9	1-360
3FLQ	X-ray	1.9	1-360
3FMH	X-ray	1.9	1-360
3FMN	X-ray	1.9	1-360
3ZYA	X-ray	1.9	1-360
5ML5	X-ray	1.9	1-360
5N67	X-ray	1.9	1-360
4DLI	X-ray	1.91	2-360
2Y8O	X-ray	1.95	1-360
3HLL	X-ray	1.95	1-360
3HV6	X-ray	1.95	2-360
3CTQ	X-ray	1.95	5-352
4KIN	X-ray	1.97	2-360
1R3C	X-ray	2	1-360
1ZYJ	X-ray	2	1-360

1R0E	X-ray	2.25	35-420
4NM5	X-ray	2.3	13-383
4NM7	X-ray	2.3	13-383
1Q3W	X-ray	2.3	2-420
3I4B	X-ray	2.3	7-420
4J71	X-ray	2.31	1-420
2JLD	X-ray	2.35	1-420
4PTG	X-ray	2.36	1-420
3ZRK	X-ray	2.37	23-393
1PYX	X-ray	2.4	1-420
5KPK	X-ray	2.4	1-420
3GB2	X-ray	2.4	34-383
3F7Z	X-ray	2.4	35-383
1O9U	X-ray	2.4	35-384
5HLP	X-ray	2.45	1-420
3ZRL	X-ray	2.48	23-393
3ZRM	X-ray	2.49	23-393
4NM0	X-ray	2.5	1-383
5F94	X-ray	2.51	36-385
5F95	X-ray	2.52	36-385
4ACD	X-ray	2.6	1-420
4ACG	X-ray	2.6	1-420
4ACH	X-ray	2.6	1-420
5KPL	X-ray	2.6	1-420
6B8J	X-ray	2.6	1-420
1GNG	X-ray	2.6	27-393
4DIT	X-ray	2.6	27-393
3F88	X-ray	2.6	35-383
3ZDI	X-ray	2.64	35-384
5KPM	X-ray	2.69	1-420
1I09	X-ray	2.7	1-420
4J1R	X-ray	2.7	1-420
3Q3B	X-ray	2.7	2-420
3SD0	X-ray	2.7	35-384
4PTC	X-ray	2.71	1-420
1Q4L	X-ray	2.77	2-420
4B7T	X-ray	2.77	35-384
1UV5	X-ray	2.8	35-384
1H8F	X-ray	2.8	35-386
2OW3	X-ray	2.8	35-386
5T31	X-ray	2.85	1-420
3L1S	X-ray	2.9	7-420
3PUP	X-ray	2.99	1-420
5HLN	X-ray	3.1	1-420
4IQ6	X-ray	3.12	1-420

1ZZ2	X-ray	2	1-360
2I0H	X-ray	2	1-360
3E92	X-ray	2	1-360

3M1S	X-ray	3.13	1-420
5OY4	X-ray	3.2	1-420
2O5K	X-ray	3.2	29-393

Protein: IGF-1R, **UniProt code**: P08069

PDB entry	Method	Resolution (Å)	Positions
1P4O	X-ray	1.5	974-1294
3LW0	X-ray	1.79	983-1286
5FXS	X-ray	1.9	980-1286
2OJ9	X-ray	2	982-1286
3I81	X-ray	2.08	982-1286
1JQH	X-ray	2.1	979-1286
3O23	X-ray	2.1	982-1286
4D2R	X-ray	2.1	985-1286
1K3A	X-ray	2.1	988-1286
3NW7	X-ray	2.11	982-1286
3NW5	X-ray	2.14	982-1286
3NW6	X-ray	2.2	982-1286
5HZN	X-ray	2.2	983-1286
5FXQ	X-ray	2.3	980-1286
3D94	X-ray	2.3	986-1286
5FXR	X-ray	2.4	980-1286
2ZM3	X-ray	2.5	981-1286
1M7N	X-ray	2.7	974-1294
3F5P	X-ray	2.9	981-1286
3QQU	X-ray	2.9	988-1286
3LVP	X-ray	3	951-1286

Protein: IGF-1R, **UniProt code**: P08069

PDB entry	Method	Resolution (Å)	Positions
4CFE	X-ray	3.02	1-552
4CFF	X-ray	3.92	1-552
4ZHX	X-ray	2.99	2-552
5ISO	X-ray	2.63	1-552
6B1U	X-ray	2.77	2-552
6B2E	X-ray	3.8	2-552

References

1. Ruiz-Torres, V.; Encinar, J.A.; Herranz-Lopez, M.; Perez-Sanchez, A.; Galiano, V.; Barrajon-Catalan, E.; Micol, V. An updated review on marine anticancer compounds: The use of virtual screening for the discovery of small-molecule cancer drugs. *Molecules* **2017**, *22*.
2. Banerjee, P.; Erehman, J.; Gohlke, B.O.; Wilhelm, T.; Preissner, R.; Dunkel, M. Super natural ii--a database of natural products. *Nucleic Acids Res* **2015**, *43*, D935-939.
3. Sterling, T.; Irwin, J.J. Zinc 15--ligand discovery for everyone. *J Chem Inf Model* **2015**, *55*, 2324-2337.
4. Sander, T.; Freyss, J.; von Korff, M.; Rufener, C. Datawarrior: An open-source program for chemistry aware data visualization and analysis. *J Chem Inf Model* **2015**, *55*, 460-473.
5. Cheng, F.; Li, W.; Zhou, Y.; Shen, J.; Wu, Z.; Liu, G.; Lee, P.W.; Tang, Y. Admetsar: A comprehensive source and free tool for assessment of chemical admet properties. *J Chem Inf Model* **2012**, *52*, 3099-3105.
6. Salentin, S.; Schreiber, S.; Haupt, V.J.; Adasme, M.F.; Schroeder, M. Plip: Fully automated protein-ligand interaction profiler. *Nucleic Acids Res* **2015**, *43*, W443-447.
